# Hypomodified tRNA in evolutionarily distant yeasts can trigger rapid tRNA decay to activate the general amino acid control response, but with different consequences

**DOI:** 10.1371/journal.pgen.1008893

**Published:** 2020-08-25

**Authors:** Thareendra De Zoysa, Eric M. Phizicky

**Affiliations:** Department of Biochemistry and Biophysics, Center for RNA Biology, University of Rochester School of Medicine, Rochester, NY, United States of America; Ohio State University, UNITED STATES

## Abstract

All tRNAs are extensively modified, and modification deficiency often results in growth defects in the budding yeast *Saccharomyces cerevisiae* and neurological or other disorders in humans. In *S*. *cerevisiae*, lack of any of several tRNA body modifications results in rapid tRNA decay (RTD) of certain mature tRNAs by the 5’-3’ exonucleases Rat1 and Xrn1. As tRNA quality control decay mechanisms are not extensively studied in other eukaryotes, we studied *trm8Δ* mutants in the evolutionarily distant fission yeast *Schizosaccharomyces pombe*, which lack 7-methylguanosine at G_46_ (m^7^G_46_) of their tRNAs. We report here that *S*. *pombe trm8Δ* mutants are temperature sensitive primarily due to decay of tRNA^Tyr(GUA)^ and that spontaneous mutations in the *RAT1* ortholog *dhp1*^*+*^ restored temperature resistance and prevented tRNA decay, demonstrating conservation of the RTD pathway. We also report for the first time evidence linking the RTD and the general amino acid control (GAAC) pathways, which we show in both *S*. *pombe* and *S*. *cerevisiae*. In *S*. *pombe trm8Δ* mutants, spontaneous GAAC mutations restored temperature resistance and tRNA levels, and the *trm8Δ* temperature sensitivity was precisely linked to GAAC activation due to tRNA^Tyr(GUA)^ decay. Similarly, in the well-studied *S*. *cerevisiae trm8Δ trm4Δ* RTD mutant, temperature sensitivity was closely linked to GAAC activation due to tRNA^Val(AAC)^ decay; however, in *S*. *cerevisiae*, GAAC mutations increased tRNA loss and exacerbated temperature sensitivity. A similar exacerbated growth defect occurred upon GAAC mutation in *S*. *cerevisiae trm8Δ* and other single modification mutants that triggered RTD. Thus, these results demonstrate a conserved GAAC activation coincident with RTD in *S*. *pombe* and *S*. *cerevisiae*, but an opposite impact of the GAAC response in the two organisms. We speculate that the RTD pathway and its regulation of the GAAC pathway is widely conserved in eukaryotes, extending to other mutants affecting tRNA body modifications.

## Author summary

tRNA modifications are highly conserved and their lack frequently results in growth defects in the yeast *Saccharomyces cerevisiae* and neuorological disorders in humans. *S*. *cerevsiaie* has two tRNA quality control decay pathways that sense tRNAs lacking modifications in the main tRNA body. One of these, the rapid tRNA decay (RTD) pathway, targets mature tRNAs for 5'-3' exonucleolytic decay by Rat1 and Xrn1. It is unknown if RTD is conserved in eukaryotes, and if it might explain phenotypes associated with body modification defects. Here we focus on *trm8Δ* mutants, lacking m^7^G_46_, in the evolutionarily distant yeast *Schizosaccharomyces pombe*. Loss of m^7^G causes temperature sensitivity and RTD in *S*. *cerevisiae*, microcephalic primordial dwarfism in humans, and defective stem cell renewal in mice. We show that *S*. *pombe trm8Δ* mutants are temperature sensitive due to tY(GUA) decay by Rat1/Dhp1, implying conservation of RTD among divergent eukaryotes. We also show that the onset of RTD triggers activation of the general amino acid control (GAAC) pathway in both *S*. *pombe* and *S*. *cerevisiae*, resulting in further tRNA loss in *S*. *pombe* and reduced tRNA loss in *S*. *cerevisiae*. We speculate that RTD and its GAAC regulation will be widely conserved in eukaryotes including humans.

## Introduction

tRNAs are subject to extensive post-transcriptional modifications that often profoundly affect tRNA function, as lack of modifications often leads to growth defects in the budding yeast *Saccharomyces cerevisiae* and to neurological or mitochondrial disorders in humans [1–5]. Many tRNA modifications in the anticodon loop are important for decoding fidelity, reading frame maintenance, and sometimes charging efficiency [6–15]. By contrast, modifications in the tRNA body, the region outside the anticodon loop, are often important for folding and stability [16–18], resulting in substantial growth defects. In *S*. *cerevisiae*, deletion of *TRM6* or *TRM61* is lethal, associated with lack of 1-methyladenosine at A_58_ (m^1^A_58_) [19], whereas deletion of *TAN1*, *TRM1*, or *TRM8* (or *TRM82*) results in temperature sensitivity associated with lack of 4-acetylcytidine at C_12_ (ac^4^C_12_), N_2_,N_2_-dimethylguanosine at G_26_ (m^2,2^G_26_), or 7-methylguanosine at G_46_ (m^7^G_46_) respectively [20–22]. Similarly, human neurological disorders are linked to mutations in *TRMT10A*, associated with reduced 1-methylguanosine at G_9_ (m^1^G_9_) [23,24], *TRMT1* (m^2,2^G_26_) [25–28], *WDR4* (m^7^G_46_) [29–31] and *NSUN2*, associated with reduced 5-methylcytidine (m^5^C) at C_48-50_, as well as at C_34_ and C_40_ [32–34].

In *S*. *cerevisiae*, lack of any of several tRNA body modifications leads to decay of a subset of the corresponding hypomodified tRNAs, mediated by either of two tRNA quality control pathways, each acting on different hypomodified tRNAs and at different stages of tRNA biogenesis. First, the nuclear surveillance pathway targets pre-tRNA_i_^Met^ lacking m^1^A, acting through the TRAMP complex and the nuclear exosome to degrade the pre-tRNA from the 3' end [17,35–37]. The nuclear surveillance pathway also targets a large portion of wild type (WT) pre-tRNAs shortly after transcription, ascribed to errors in folding of the nascent tRNA or to mutations arising during transcription [38]. Second, the rapid tRNA decay (RTD) pathway targets a subset of the mature tRNAs lacking m^7^G_46_, m^2,2^G_26_, or ac^4^C_12_, using the 5'-3' exonucleases Rat1 in the nucleus and Xrn1 in the cytoplasm [18,21,22,39,40]. RTD is inhibited by a *met22Δ* mutation [22,39,41,42] due to accumulation of the Met22 substrate adenosine 3', 5' bisphosphate (pAp) [43,44], which binds the active site of Xrn1 and presumably Rat1 [45]. The RTD pathway also targets fully modified tRNAs with destabilizing mutations in the stems, particularly the acceptor and T-stem, which expose the 5' end [40–42]. The hypomodified tRNAs targeted by the RTD pathway also expose the 5' end, ascribed to destabilization of the tertiary fold [40].

There is limited evidence documenting tRNA quality control decay pathways that act on hypomodified tRNAs in other eukaryotes. A mouse embryonic stem cell line with a knockout of *METTL1* (ortholog of *S*. *cerevisiae TRM8*) had undetectable m^7^G in its tRNA substrates and reduced levels of several METTL1 substrate tRNAs [46]. Similarly, knockdown of *METTL1* and *NSUN2* (homolog of *S*. *cerevisiae TRM4*) in HeLa cells led to reduced levels of tRNA^Val(AAC)^ (abbreviated tV(AAC), as in the *Saccharomyces* genome database) at 43°C in the presence of 5-fluorouracil (5-FU) [47], a known inhibitor of pseudouridine synthases and 5-methyluridine methyltransferase [48–50]. However, in both of these cases, the underlying mechanism is not known. It was also shown that WT mature tRNAi^Met^ was subject to decay by Xrn1 and Rat1 after 43°C heat shock in HeLa cells, although there was no change in the modification pattern *in vivo* or in the stability of the tRNA *in vitro* caused by this temperature shift [51].

The goal of the work described here is to determine if and to what extent tRNA quality control decay pathways are linked to hypomodified tRNAs in eukaryotes other than *S*. *cerevisiae*. To address this issue, we have studied the biology of the tRNA m^7^G_46_ methyltransferase Trm8 in the fission yeast *Schizosaccharomyces pombe*, which diverged from *S*. *cerevisiae ~* 600 million years ago [52].

We chose to study *S*.*pombe* Trm8 because *S*. *cerevisiae trm8Δ* mutants were known to trigger decay by the RTD pathway. *S*. *cerevisiae* Trm8 forms a complex with Trm82 that is required for formation of m^7^G_46_ in eukaryotic tRNAs [53,54]. *S*. *cerevisiae trm8Δ* and *trm82Δ* mutants are each modestly temperature sensitive [20], and *trm8Δ* or *trm82Δ* mutants also lacking any of several other body modifications had enhanced temperature sensitivity [18]. Moreover, the temperature sensitivity of *trm8Δ* mutants was suppressed by a *met22Δ* mutation and was associated with decay of tV(AAC) [22], and the more severe temperature sensitivity of *trm8Δ trm4Δ* mutants (lacking both m^7^G and m^5^C) was shown explicitly to be due to RTD of tV(AAC) [18,39]. In addition, in mammalian cells, Trm8 biology has other dimensions of complexity. The human *TRM82* ortholog *WDR4* was associated with reduced tRNA m^7^G modification and a distinct form of microcephalic primordial dwarfism [29]; *METTL1* or *WDR4* knock out mouse embryonic stem cells showed defects in self renewal and differentiation [46]; and METTL1 was also responsible for m^7^G modification of mammalian miRNAs and mRNAs [55,56]. This evidence emphasizes that Trm8/Trm82 (METTL1/WDR4) and/or its m^7^G modification product is important in *S*. *cerevisiae* and mammals, although the reasons are not yet known beyond *S*. *cerevisiae*.

We find here that *S*. *pombe trm8Δ* mutants have a temperature sensitive growth defect due primarily to decay of tRNA^Tyr(GUA)^ (tY(GUA)) and to some extent tRNA^Pro(AGG)^ (tP(AGG)) by the Rat1 ortholog Dhp1, demonstrating that a major component of the RTD pathway is conserved between *S*. *pombe* and *S*. *cerevisiae*. We also find an unexpected connection between the RTD pathway and the general amino acid control (GAAC) pathway in both *S*. *pombe* and *S*. *cerevisiae*. In both *S*. *pombe trm8Δ* mutants and *S*. *cerevisiae trm8Δ trm4Δ* mutants, the temperature sensitivity coincides with the onset of tRNA decay, which in turn triggers the GAAC activation, presumably due to the increased stress from the tRNA decay. However, in *Sp trm8Δ* mutants, GAAC activation is deleterious to growth, as mutations in the GAAC pathway restore growth and tRNA levels, whereas in *S*. *cerevisiae trm8Δ trm4Δ* mutants, GAAC pathway activation is beneficial, as GAAC mutations exacerbate the growth defect and accelerate tRNA loss. Thus, our results demonstrate a conserved GAAC response associated with tRNA decay by the RTD pathway, but opposite effects on cell physiology in the two organisms. These findings suggest the widespread conservation of the RTD pathway in eukaryotes, and its linkage to the GAAC pathway.

## Results

### The *S*. *pombe trm8Δ* mutants lack m^7^G in tRNAs and are temperature sensitive

**Fig 1:**
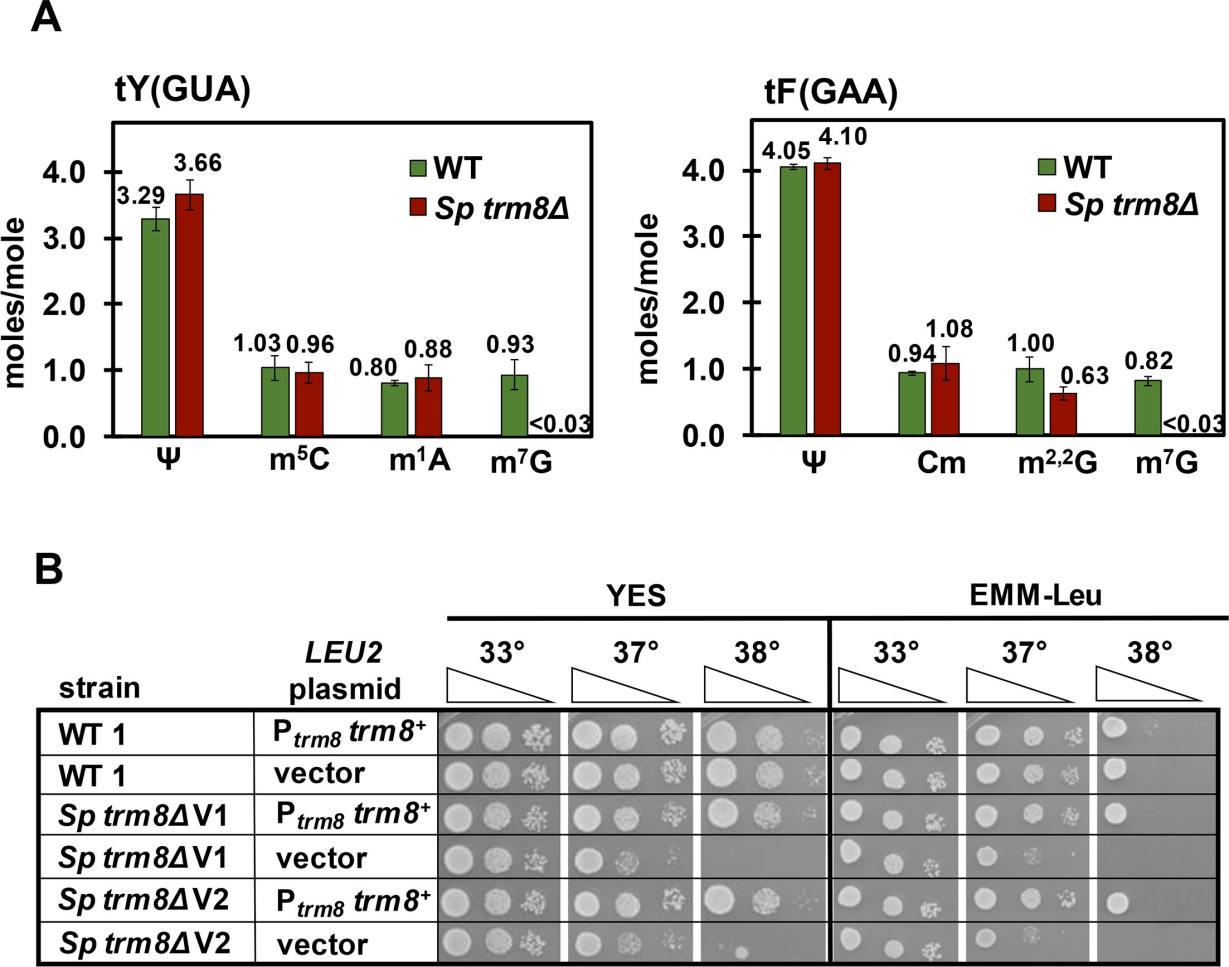
*S*. *pombe trm8Δ* mutants lack m^7^G and are temperature sensitive. *(A) trm8Δ* mutants have no detectable m^7^G in their tY(GUA) and tF(GAA). *S*. *pombe trm8Δ* mutants and WT cells were grown in biological triplicate in YES media at 30°C and tRNAs were purified, digested to nucleosides, and analyzed for modifications by HPLC as described in Materials and Methods. The bar chart depicts average moles/mol values of nucleosides with associated standard deviation; WT, green; *S*. *pombe* (*Sp*) *trm8Δ*, brown. ***(B) trm8Δ* mutants are temperature sensitive due to lack of *trm8***^***+***^. Strains with plasmids as indicated were grown overnight in EMM-Leu media at 30°C, diluted to OD_600_ ~ 0.5, serially diluted 10-fold in EMM-Leu, and 2 μL was spotted onto plates containing EMM-Leu or YES media and incubated at 33°C, 37°C, and 38°C. The two independent *trm8Δ* mutants were labeled as *Sp trm8Δ* V1 and V2.

As Trm8 is the catalytic subunit of the Trm8-Trm82 complex [20], we anticipated that tRNAs from *S*. *pombe trm8Δ* mutants would lack m^7^G. We purified tY(GUA) and tF(GAA), which had each been previously shown to have m^7^G_46_ [57,58], and then analyzed their nucleosides by HPLC analysis. Purified tY(GUA) from *S*. *pombe trm8Δ* mutants had no detectable m^7^G levels (less than 0.03 moles/mole), compared to near stoichiometric levels in tY(GUA) from WT cells (0.93 +/- 0.22 moles/mole), whereas levels of each of three other analyzed modifications (pseudouridine (Ψ), m^5^C, and m^1^A) were very similar in *trm8Δ* and WT cells (Fig 1A). Similarly, purified tF(GAA) from *trm8Δ* mutants had no detectable levels of m^7^G compared to near stoichiometric levels in WT cells, but otherwise WT levels of Ψ, 2'-O-methylcytidine (Cm) and m^2,2^G (Fig 1A). These results suggest strongly that *S*. *pombe trm8*^*+*^ is the methyltransferase responsible for m^7^G formation in cytoplasmic tRNAs.

To understand the biology of *S*. *pombe trm8Δ* mutants, we examined the growth phenotypes of two genetically independent *trm8Δ* mutants. *trm8Δ* mutants were temperature sensitive starting at 37°C on rich (YES) and minimal (EMM) media, and expression of P_*trm8*_ *trm8*^*+*^ on a plasmid restored WT growth in both media (Fig 1B). Thus, the temperature sensitivity of *trm8Δ* mutants was due to lack of *trm8*^*+*^.

### *S*. *pombe trm8Δ* mutants have reduced levels of tP(AGG) and tY(GUA) at high temperatures

To determine if the temperature sensitivity of *S*. *pombe trm8Δ* mutants was associated with tRNA decay, we analyzed tRNA levels of *trm8Δ* mutants after an 8 hour temperature shift in YES media from 30°C to 36.5°C, 37.5°C, and 38.5°C, which progressively inhibited growth (S1 Fig). We measured tRNA levels of all 21 tRNAs in the Genomic tRNA Database [59] that had a 5-nt variable loop with a central guanosine residue (S1 Table), which is the signature for m^7^G modification [60]. We quantified levels of each tRNA at each temperature relative to the levels of that tRNA in WT cells at 30°C, after normalization of each to the levels of the non-Trm8 substrate tG(GCC) at the corresponding temperature. We used tG(GCC) as the standard because, for unknown reasons, the usual standards 5S and 5.8S RNA each had temperature-dependent reduction in their levels in *trm8Δ* mutants (S2 Fig), as determined relative to input RNA levels. Note that with tG(GCC) as the standard, the levels of another non-Trm8 substrate, tL(UAA), were also unaffected.

**Fig 2:**
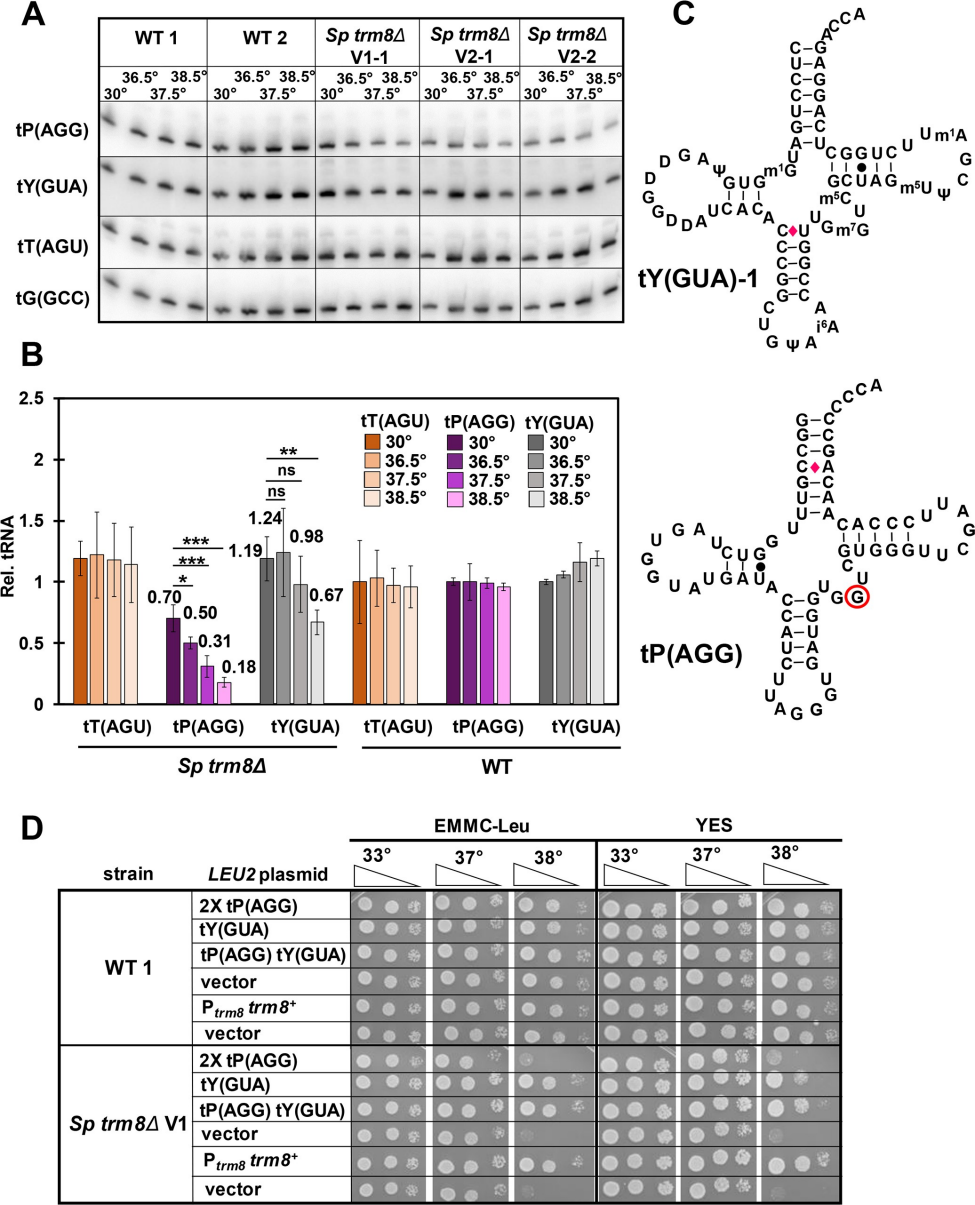
*S*. *pombe trm8Δ* mutants have reduced levels of tP(AGG) and tY(GUA) at elevated temperatures. ***(A)* Northern analysis of Trm8 substrates tP(AGG), tY(GUA), and tT(AGU) in *trm8Δ* and WT cells after shift from 30°C to 36.5°C, 37.5°C, and 38.5°C.** Strains were grown in YES media at 30°C, shifted to the indicated temperatures for 8 hours as described in Materials and Methods, and RNA was isolated and analyzed by northern blotting. n = 2 for WT cells, n = 3 for *S*. *pombe trm8Δ* mutants. ***(B)* Quantification of tP(AGG), tY(GUA), and tT(AGU) levels in WT and *trm8Δ* mutants at different temperatures.** The bar chart depicts relative levels of tRNA species at each temperature, relative to their levels in the WT strain at 30°C (each value itself first normalized to levels of the control non-Trm8 substrate tG(GCC)). For each tRNA, lighter shades indicate progressively higher temperatures (30°C, 36.5°C, 37.5°C to 38.5°C) for tT(AGU), brown; tP(AGG), purple; tY(GUA), gray. Standard deviations for each tRNA measurement are indicated. The statistical significance of tRNA levels was evaluated using a two-tailed Student’s t-test assuming equal variance. ns, not significant (p > 0.05); *, p < 0.05; **, p < 0.01; ***, p < 0.001. *(C)* Schematic of the secondary structure of tY(GUA)-1 and tP(AGG). Modifications of tY(GUA) are as annotated. WC base pairs, black lines; GU base pairs, black dots; mismatch C-A or C-U base pairs, red diamonds; presumed m^7^G_46_, red circle. *(D)* Overproduction of tY(GUA), but not tP(AGG), suppressed the temperature sensitive growth defect of *trm8Δ* mutants. Strains with plasmids as indicated were grown overnight in EMMC-Leu media at 30°C and analyzed for growth as in Fig 1B on the indicated plates.

Northern analysis showed that *S*. *pombe trm8Δ* mutants had significantly reduced levels of two of the 21 potential Trm8 substrate tRNAs as the temperature was increased. The levels of tP(AGG) were substantially reduced in *trm8Δ* mutants, from 70% of the levels in WT cells at 30°C, to 50%, 31%, and 18% after temperature shift to 36.5°C, 37.5°C, and 38.5°C respectively, whereas levels of tP(AGG) in WT cells remained constant as temperature increased (Fig 2A and 2B). As expected, tP(AGG) is indeed a substrate of Trm8, since purified tP(AGG) from *trm8Δ* mutants had undetectable levels of m^7^G, but WT levels of each of three other modifications (S3 Fig). The levels of tY(GUA) were also reduced in *trm8Δ* mutants as temperature increased, albeit to a lesser extent than tP(AGG) levels. Levels of tY(GUA) in *trm8Δ* mutants were about the same as those in WT cells at 30°C (119%), remained essentially unchanged at 36.5°C and 37.5°C (124%, and 98%), but were reduced to 67% at 38.5°C, whereas tY(GUA) levels in WT cells were relatively constant at all temperatures. In contrast, none of the 19 other predicted Trm8 substrate tRNAs showed a temperature-dependent reduction in levels in *trm8Δ* mutants (Figs 2A and 2B and S4 and S5). Levels of 15 tRNAs were approximately constant in *trm8Δ* mutants as temperature increased, although the initial levels varied somewhat, and levels of the other four tRNAs (tR(CCU), tM_e_(CAU), tV(CAC), and tK(UUU)) were modestly increased at 38.5°C. Thus, if the temperature sensitivity of *trm8Δ* mutants was due to loss of tRNAs, the likely candidates were tP(AGG) and tY(GUA).

### The growth defect of *S*. *pombe trm8Δ* mutants is primarily due to loss of tY(GUA)

To evaluate the cause of the temperature sensitivity of *S*. *pombe trm8Δ* mutants, we analyzed growth after overexpression of tP(AGG) and/or tY(GUA) on *leu2*^*+*^ plasmids (Fig 2C). Surprisingly, on both YES media and EMM complete (EMMC) media lacking leucine, *trm8Δ* mutants expressing tY(GUA) grew almost as well as the *trm8Δ* [P_*trm8*_ *trm8*^*+*^] strain or the WT strain at elevated temperatures, whereas *trm8Δ* mutants expressing two tP(AGG) genes on a plasmid had little effect on the temperature sensitivity (Fig 2D). As expected, northern analysis showed that *trm8Δ* [*leu2*^*+*^ tY(GUA)] strains had substantially more tY(GUA) than the *trm8Δ* [*leu2*^*+*^] vector control strain at 30°C and 38.5°C (3.2-fold and 6.8-fold more respectively) (S6 Fig). Similarly, *trm8Δ* mutants expressing two copies of tP(AGG) had more tP(AGG) at 30°C and 38.5°C than the vector control, and the levels of the control tT(AGU) were unchanged in all strains at both temperatures. We conclude that although levels of both tY(GUA) and tP(AGG) were reduced in *trm8Δ* mutants at elevated temperatures in both YES and EMMC media, tY(GUA) is the major physiologically important tRNA for these phenotypes.

Although tY(GUA) overexpression almost completely restored growth of *S*. *pombe trm8Δ* mutants in YES and EMMC media at 38°C and 39°C, expression of both tY(GUA) and tP(AGG) was required to completely suppress the growth defects in YES + glycerol media (S7 Fig). By contrast, overexpression of tY(GUA) and tP(AGG) had no effect on the known temperature sensitivity of *trm8Δ* mutants in YES media containing 5-FU [61,62] (S7 Fig), perhaps due to reduced levels of Ψ and 5-methyluridine modifications, which could trigger decay of other hypomodified tRNA species in *trm8Δ* mutants.

### *dhp1* mutations suppress the *S*. *pombe trm8Δ* growth defect and restore tY(GUA) and tP(AGG) levels

**Fig 3:**
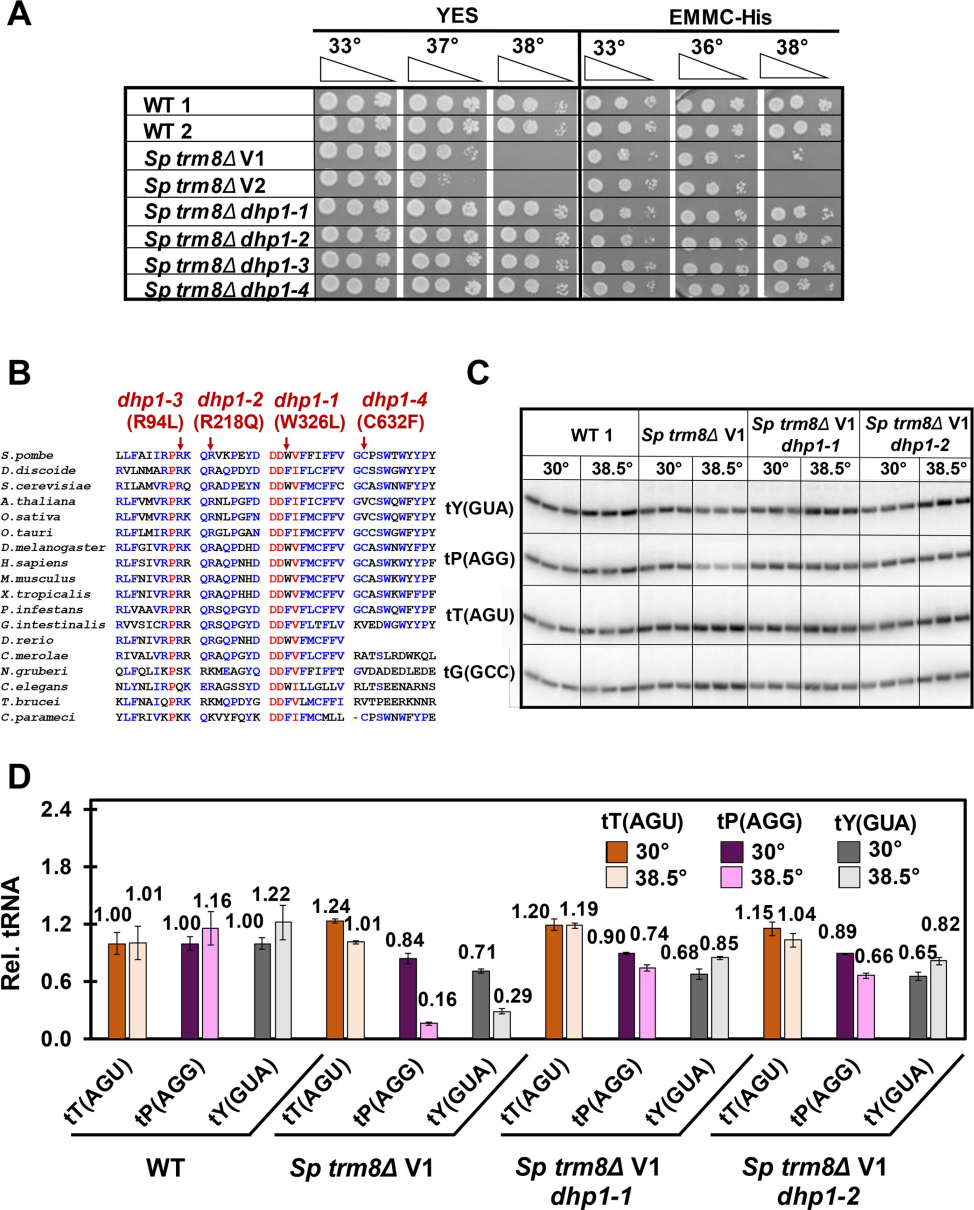
Mutations in *dhp1* suppress the temperature sensitivity of *S*. *pombe trm8Δ* mutants and restore tP(AGG) and tY(GUA) levels. **(*A*) *dhp1* mutations restored growth of *trm8Δ* mutants at high temperature.** Strains as indicated were grown overnight in YES media at 30°C and analyzed for growth as in Fig 1B. **(*B*) Mutations in *dhp1* that restored growth of *S*. *pombe trm8Δ* mutants reside in evolutionarily conserved residues.** The amino acid sequence of *Sp* Dhp1 was aligned with putative Rat1/Dhp1 orthologs from 17 evolutionarily distant eukaryotes, using MultAlin (http://multalin.toulouse.inra.fr/multalin/) [135]. red, > 90% conservation; blue, 50%-90% conservation. Alleles of *dhp1* mutations are indicated at the top. ***(C*) Each of two *trm8Δ dhp1* mutants had restored tRNA levels in YES media at 38.5°C.** Strains were grown in YES media at 30°C and shifted to 38.5°C for 8 hours, and RNA was isolated and analyzed by northern blotting as in Fig 2A. ***(D)* Quantification of tRNA levels of *trm8Δ dhp1* mutants shown in Fig 3C.** tRNA levels were quantified as in Fig 2B. tT(AGU), brown; tP(AGG), purple; tY(GUA), gray; dark shades, 30°C; light shades, 38.5°C.

To identify the mechanisms that restore growth to *S*. *pombe trm8Δ* mutants at elevated temperatures, we isolated and analyzed spontaneous suppressors of the temperature sensitivity. One major class of four *trm8Δ* suppressors were as temperature resistant as WT on YES and EMMC media, and nearly as resistant as WT on YES + 5-FU media (Figs 3A and S8). Genome sequencing revealed that these mutants each had distinct missense mutations in the *RAT1* ortholog *dhp1*^*+*^. The *dhp1* mutations each occurred in highly conserved residues, based on an alignment of 18 *RAT1*/*dhp1*^*+*^ eukaryotic homologs from multiple phyla (Fig 3B), and are presumably partial loss of function mutations as *S*. *pombe dhp1*^*+*^, like *S*. *cerevisiae RAT1*, is an essential gene [63,64].

Because we obtained four genetically independent *S*. *pombe trm8Δ dhp1* mutants and very few other mutations in the whole genome sequencing, it was highly likely that the *dhp1* mutations were responsible for the restoration of growth in *trm8Δ dhp1* mutants. Consistent with this, a plasmid expressing *dhp1*^+^ complemented the *S*. *pombe trm8Δ dhp1-1* suppressor, resulting in temperature sensitivity, but had no effect on WT or *trm8Δ* mutants (S9 Fig). Thus, we conclude that the *dhp1* mutations were responsible for the rescue of growth at high temperature.

As Dhp1 encodes a 5'-3' exonuclease [65], it seemed highly likely that the *S*. *pombe trm8Δ dhp1* mutants prevented decay of tY(GUA) and tP(AGG) at non-permissive temperature. Indeed, we found that for each of two *dhp1* suppressors, tY(GUA) levels were almost completely restored at 38.5°C, from 29% in the *trm8Δ* mutant to 85% and 82% in the *trm8Δ dhp1-1* and *trm8Δ dhp1-2* strains respectively (Fig 3C and 3D). Similarly, tP(AGG) levels were virtually completely restored at 38.5°C, from 16% in the *trm8Δ* mutant to 74% and 66% in the *trm8Δ dhp1* suppressors, and the levels of the control tRNA (tT(AGU)) was unaffected. Similar restoration of tY(GUA) and tP(AGG) levels was also observed in the two other *trm8Δ dhp1* suppressors at 38.5°C (S10 Fig). We conclude that, as for RTD in *S*. *cerevisiae* modification mutants [22,39,40], tRNA decay in *S*. *pombe trm8Δ* mutants occurs by 5'-3' exonucleolytic degradation of tRNA, providing strong evidence for conservation of the RTD pathway in *S*. *pombe*.

### Mutations in the GAAC pathway suppress the *S*. *pombe trm8Δ* growth defect and restore tRNA levels

**Fig 4:**
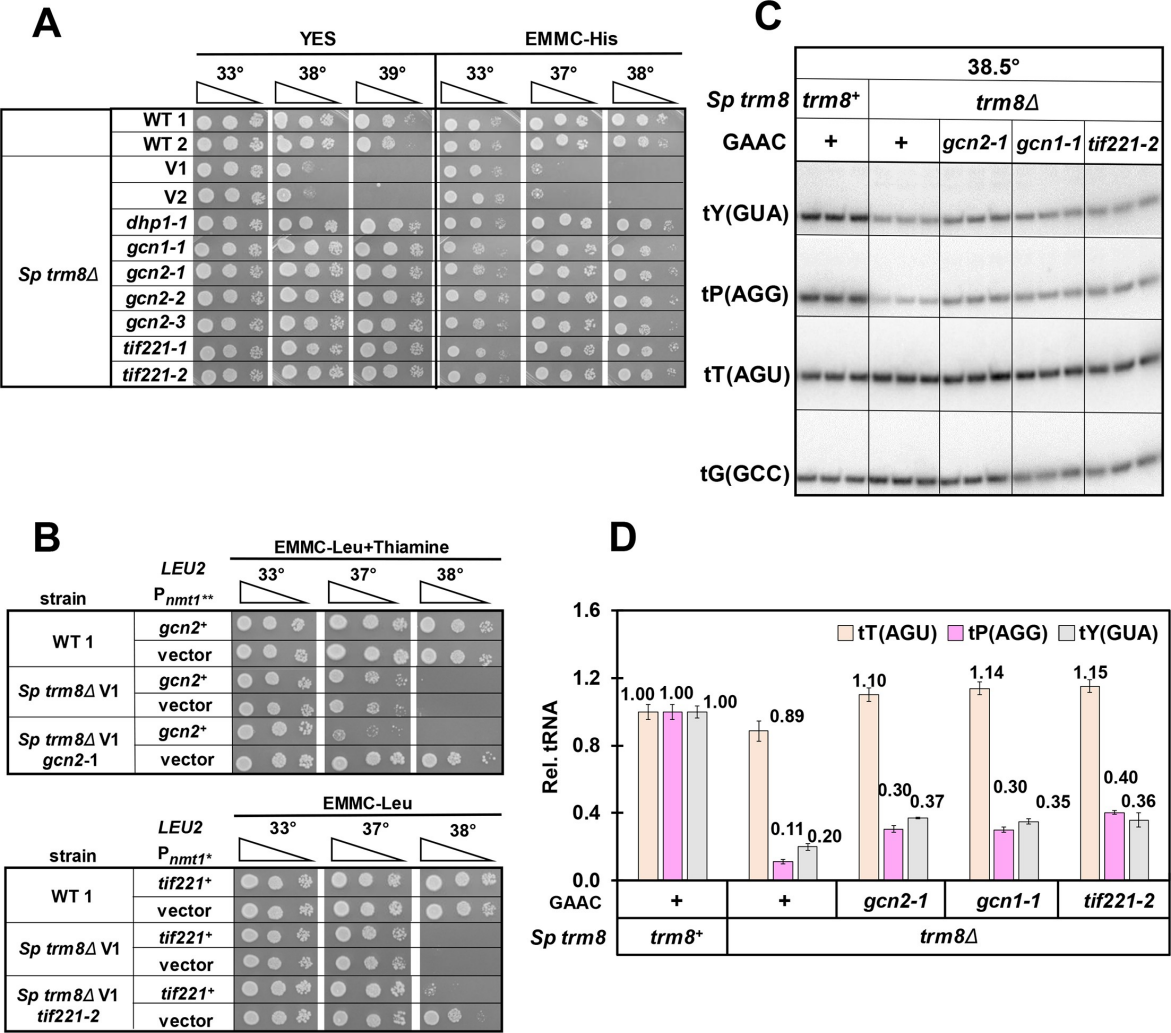
Mutations in the GAAC pathway suppress the temperature sensitivity of *S*. *pombe trm8Δ* mutants and restore tP(AGG) and tY(GUA) levels. **(*A*) *gcn1*, *gcn2*, and *tif221* mutations each restored growth of *trm8Δ* mutants at high temperature.** Strains as indicated were grown overnight in YES media at 30°C and analyzed for growth as in Fig 1B. ***(B*) Expression of *gcn2***^***+***^ **and *tif221***^***+***^ **complemented the suppression phenotype of *trm8Δ gcn2-1* and *trm8Δ tif221-2* mutants respectively.** *S*. *pombe trm8Δ gcn2-1* and *trm8Δ tif221-2* mutants expressing *gcn2*^*+*^ and *tif221*^*+*^ respectively, or a vector, were grown overnight in EMMC-Leu media at 30°C, and analyzed for growth as in Fig 1B. Note that expression of *gcn2*^*+*^ was kept to modest levels by adding thiamine to the media to partially suppress overexpression from the P_nmt1_** promoter. ***(C) gcn1*, *gcn2*, and *tif221* mutations each partially restored tY(GUA) and tP(AGG) levels of *trm8Δ* mutants**. Strains were grown in YES media at 30°C and shifted to 38.5°C for 8 hours as described in Materials and Methods, and RNA was isolated and analyzed by northern blotting. ***(D)* Quantification of tRNA levels of *trm8Δ* GAAC mutants shown in Fig 4C.** tRNA levels were quantified as in Fig 2B. tT(AGU), brown; tP(AGA), purple; tY(GUA), gray.

A second major group of six *S*. *pombe trm8Δ* suppressors was temperature resistant on YES and EMMC media, but sensitive on YES + 5-FU media, and genome sequencing showed that these suppressors each had distinct mutations in elements of the GAAC pathway (Figs 4A and S11). Among these, we found three *trm8Δ* suppressors with *gcn2* mutations, one with a *gcn1* mutation, and two with *tif221* mutations, encoding the translation initiation factor eIF2Bα (S2 Table). Each of these genes in *S*. *cerevisiae* is known to be critical for the GAAC pathway [66–68], which is widely conserved in eukaryotes, including *S*. *pombe* and mammals [69–74]. In this pathway, amino acid starvation leads to uncharged tRNAs that bind Gcn2 to activate its kinase domain, phosphorylation of eIF2α by Gcn2, global repression of translation, and derepression of translation of the transcription factor Gcn4, resulting in increased transcription of nearly one tenth of the *S*. *cerevisiae* genes [66,75–78]. A similar massive transcription program change occurs in *S*. *pombe* after amino acid starvation [79].

As expected, all *S*. *pombe trm8Δ* mutants with suppressing mutations in *gcn2*, *gcn1*, or *tif221*, grew poorly on media containing 3-Amino-1,2,4-triazole (3-AT) (S11 Fig), the classical inducer of the GAAC pathway [79–81]. Furthermore, each of two *S*. *pombe trm8Δ* GAAC suppressors tested (with *gcn2-1* and *tif221-2* mutations) was complemented by re-introduction of the WT gene (Fig 4B), and a re-constructed *trm8Δ gcn2Δ* strain was temperature resistant and sensitive to 3-AT (S12 Fig).

Consistent with their role as *S*. *pombe trm8Δ* suppressors, all six of the *trm8Δ* GAAC mutants had increased levels of tY(GUA) and tP(AGG) at high temperature. After growth in YES media at 38.5°C, all *trm8Δ* GAAC mutants showed a 1.7-fold to 2.1-fold increase in tY(GUA) levels compared to the parent *trm8Δ* mutant, and tP(AGG) levels were increased ~ 3 fold, whereas the controls tT(AGU) and tV(AAC) did not have increased levels (Figs 4C and 4D and S13). These results provided strong evidence that the effect of the GAAC mutants was to increase tRNA levels to restore growth.

To test if the temperature resistance of *S*.*pombe trm8Δ* GAAC mutants extended further downstream within the GAAC pathway, we deleted the GAAC transcription factor *fil1*^*+*^, the functional equivalent of *S*. *cerevisiae* Gcn4 [82]. We found that *trm8Δ filΔ* mutants were distinctly more temperature resistant than *trm8Δ* mutants in EMMC-His media, but not as temperature resistant as a *trm8Δ gcn2Δ* mutant (S14 Fig). We thus infer that suppression of the *trm8Δ* temperature sensitivity observed in *S*. *pombe trm8Δ* GAAC mutants is due in part to transcription activation by Fil1. We note that the *filΔ* mutation did not rescue the temperature sensitivity of *trm8Δ* mutants in YES media; this could be due in part to the temperature sensitivity of *fil1Δ* mutants in YES media.

### The temperature sensitivity of *S*. *pombe trm8Δ* mutants coincides precisely with the onset of GAAC activation and tY(GUA) decay

Since *S*. *pombe trm8Δ* suppressors in different components of the GAAC pathway all restored tY(GUA) and tP(AAG) levels, we inferred that *trm8Δ* mutants activated the GAAC pathway at non-permissive temperatures, and that this activation somehow promoted further loss of the tRNA. To establish the precise connection between growth, tRNA levels, and GAAC activation in *trm8Δ* mutants, we measured each parameter during liquid growth in rich media at a permissive temperature (30°C), and at three elevated temperatures: 36.5°C, 37.5°C, and 38.5°C. In this experiment, *trm8Δ* mutants grew virtually identically to WT control strains at 36.5°C and 37.5°C, and the growth defect was only obvious at 38.5°C (S15 Fig).

**Fig 5:**
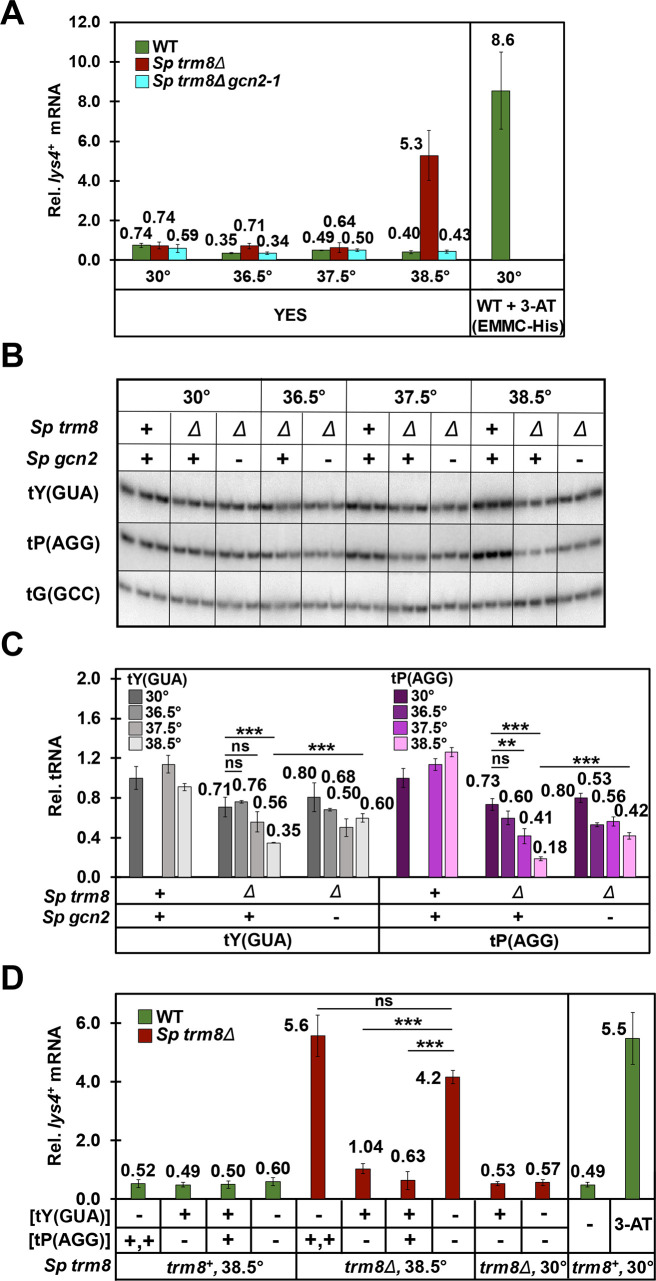
The temperature sensitivity of *S*. *pombe trm8Δ* mutants is associated with induction of the GAAC pathway due to tY(GUA) decay. ***(A) S*. *pombe trm8Δ* mutants induced *lys4***^***+***^ **mRNA expression at 38.5°C but not at 36.5°C or 37.5°C.** Strains as indicated were grown in YES media at 30°C and shifted to 36.5°C, 37.5°C, or 38.5°C for 8 hours (S15 Fig), and bulk RNA was isolated and analyzed by RT-qPCR as described in Materials and Methods. The mRNA levels of *lys4*^*+*^ were normalized to levels of *act1*^*+*^, a non-regulated control mRNA. WT, green; *Sp trm8Δ*, brown; *Sp trm8Δ gcn2-1*, light blue. Right side: GAAC induction of WT cells grown at 30°C in EMMC-His and treated with 10 mM 3-AT for 4 hours, evaluated in parallel. ***(B)* An *S*. *pombe trm8Δ gcn2-1* mutant had restored levels of tY(GUA) and tP(AGG) at 38.5°C**. Bulk RNA from the growth done for Fig 5A was used for the northern analysis. ***(C)* Quantification of tY(GUA) and tP(AGG) levels in WT, *trm8Δ*, and *trm8Δ gcn2-1* mutants at different temperatures.** tRNA levels were quantified as described in Fig 2B. ***(D)* tY(GUA) overproduction repressed the GAAC induction of *trm8Δ* mutants at 38.5°C.** Strains as indicated with plasmids expressing tY(GUA) and/or tP(AGG) were grown in EMMC-Leu media at 30°C and shifted to 38.5°C for 8 hours, and then RNA was isolated and *lys4*^*+*^ mRNA levels were analyzed by RT-qPCR as described in Fig 5A. Right side: GAAC induction of WT cells grown at 30°C in EMMC-His media, and treated with 10 mM of 3-AT for 4 hours, evaluated in parallel to other samples.

Strikingly, *S*. *pombe trm8Δ* mutants activated the GAAC pathway only at 38.5°C, the lowest temperature at which the growth defect was obvious. We measured GAAC activation by measuring mRNA levels of the known GAAC targets *lys4*^*+*^ and *aro8*^*+*^ (SPAC56E4.03) [79], which we had previously used [83]. At 38.5°C in *trm8Δ* mutants, we observed a 7.1-fold increase in *lys4*^*+*^ mRNA levels (relative to the standard *act1*^*+*^), compared to that from WT or *trm8Δ* mutants at 30°C (5.3 vs 0.74 and 0.74) (Fig 5A). By contrast, we found no measurable change in *lys4*^*+*^ mRNA levels in *trm8Δ* mutants grown at 36.5°C and 37.5°C, relative to that observed in *trm8Δ* mutants at 30°C, or in WT cells at any temperature. Moreover, the increase in relative *lys4*^*+*^ mRNA levels in *trm8Δ* mutants in YES media at 38.5°C was almost as high as that observed in WT cells induced with 3-AT, and was completely eliminated in *trm8Δ gcn2-1* mutants. Examination of relative *aro8*^*+*^ mRNA levels gave a similar result (S16 Fig): a substantial Gcn2-dependent increase in relative *aro8*^*+*^ mRNA levels at 38.5°C in *trm8Δ* mutants, relative to 37.5°C (1.5 vs 0.45), and no change in relative *aro8*^*+*^ mRNA levels at 37.5°C in *trm8Δ* mutants compared to WT (0.45 vs 0.44). Consistent with the appearance of the *S*. *pombe trm8Δ* growth defect and the GAAC activation only at 38.5°C, tY(GUA) decay was only significant in YES media at 38.5°C, and at that temperature the *gcn2-1* mutation significantly restored tY(GUA) levels (Fig 5B and 5C).

As anticipated, phosphorylation of eIF2α tracked with GAAC activation. We grew WT and *S*. *pombe trm8Δ* mutants at 30°C and 38.5°C, and measured both eIF2α phosphorylation levels and GAAC activation of *lys4*^*+*^ and *aro8*^*+*^ mRNA expression. We observed much more pronounced levels of eIF2α phosphorylation in *trm8Δ* mutants at 38.5°C, compared to modest phosphorylation levels in WT at 38.5°C, and much reduced phosphorylation in both *trm8Δ* mutants and WT at 30°C (S17A Fig). Analysis of the same samples by RT-qPCR showed a substantial increase of *lys4*^*+*^ and *aro8*^*+*^ mRNAs in *trm8Δ* mutants at 38.5°C, but not in WT at 38.5°C or in either *trm8Δ* or WT at 30°C (S17B Fig), just as we observed in Figs 5A and S16. These results provide evidence that the Gcn2 mediated GAAC activation of expression of *lys4*^*+*^ and *aro8*^*+*^ mRNAs occurs through eIF2α phophorylation in *trm8Δ* mutants at 38.5°C.

As tY(GUA) was the major physiologically relevant substrate in YES media (Fig 2D), we speculated that at 38.5°C, tY(GUA) decay might be driving the GAAC activation associated with the *trm8Δ* growth defect. Alternatively, GAAC activation could be a consequence of both tY(GUA) and tP(AAG) decay, reduced tRNA charging associated with *trm8Δ* mutants at high temperature, or partly as a consequence of temperature stress itself, as a number of different stress conditions are known to activate the GAAC pathway [72,74,84,85].

To determine the extent to which reduced tRNA levels activated the GAAC pathway, we examined GAAC induction of *S*. *pombe trm8Δ* strains after overproduction of tY(GUA) and tP(AGG), using the same samples we used to show that overproduction of tY(GUA) suppressed the *trm8Δ* temperature sensitivity (Figs 2D and S6). As expected, relative *lys4*^*+*^ mRNA levels were increased in *trm8Δ* [vector] strains grown at 38.5°C, compared to this strain at 30°C (4.2 vs 0.57, 7.4-fold) or to WT at 38.5°C (7.0 fold), indicating GAAC activation (Fig 5D). Notably, relative *lys4*^*+*^ mRNA levels were reduced 4.0-fold in the *trm8Δ* [tY(GUA)] strain compared to the corresponding *trm8Δ* [vector] strain (from 4.2 to 1.04), and were not reduced in the *trm8Δ* [tP(AGG)] strain. Based on these results, we conclude that reduced function of tY(GUA) is the primary cause of GAAC activation and temperature sensitivity of *trm8Δ* mutants at 38.5°C. As charging of tY(GUA) was distinctly but marginally reduced at 38.5°C relative to WT (S18 Fig), we cannot determine if the GAAC activation was due to reduced levels of tY(GUA), or to a combination of reduced levels and charging [69,76,80,83,86].

To determine the effects of Trm8 and Gcn2 on processing and transcription, we examined expression of the four pre-tY(GUA) species (1–1, 1–2, 1–3, and 2, as described [59]) in the WT, *trm8Δ*, and *trm8Δ gcn2-1* strains, using appropriate intron-specific probes. With a probe specific for the pre-tY(GUA)-2 species, we found that at all temperatures *trm8Δ* mutants accumulated significantly more of the 3' end-extended and the end-matured pre-tY(GUA) species than the WT strains (S19 Fig). Similarly, with the pre-tY(GUA)-1-3 probe, we observed accumulation of the end-matured pre-tY(GUA) in *trm8Δ* mutants at all temperatures. These results suggest a processing defect due to lack of m^7^G for these pre-tY(GUA) species. We also found that levels of the primary transcript, corresponding to the largest pre-tY(GUA) species, were slightly elevated in *trm8Δ gcn2-1* mutants relative to *trm8Δ* mutants, at both 37.5°C and 38.5°C, but not at 36.5°C (S19A and S19B Fig). This result suggests that tRNA transcription might play some role in restoring tY(GUA) levels in a *trm8Δ gcn2-1* mutant at 37.5°C and 38.5°C, although it is not clear yet if this effect accounts for all of the suppression.

### In *S*. *cerevisiae*, mutation of the GAAC pathway exacerbates the effects of the RTD pathway

**Fig 6:**
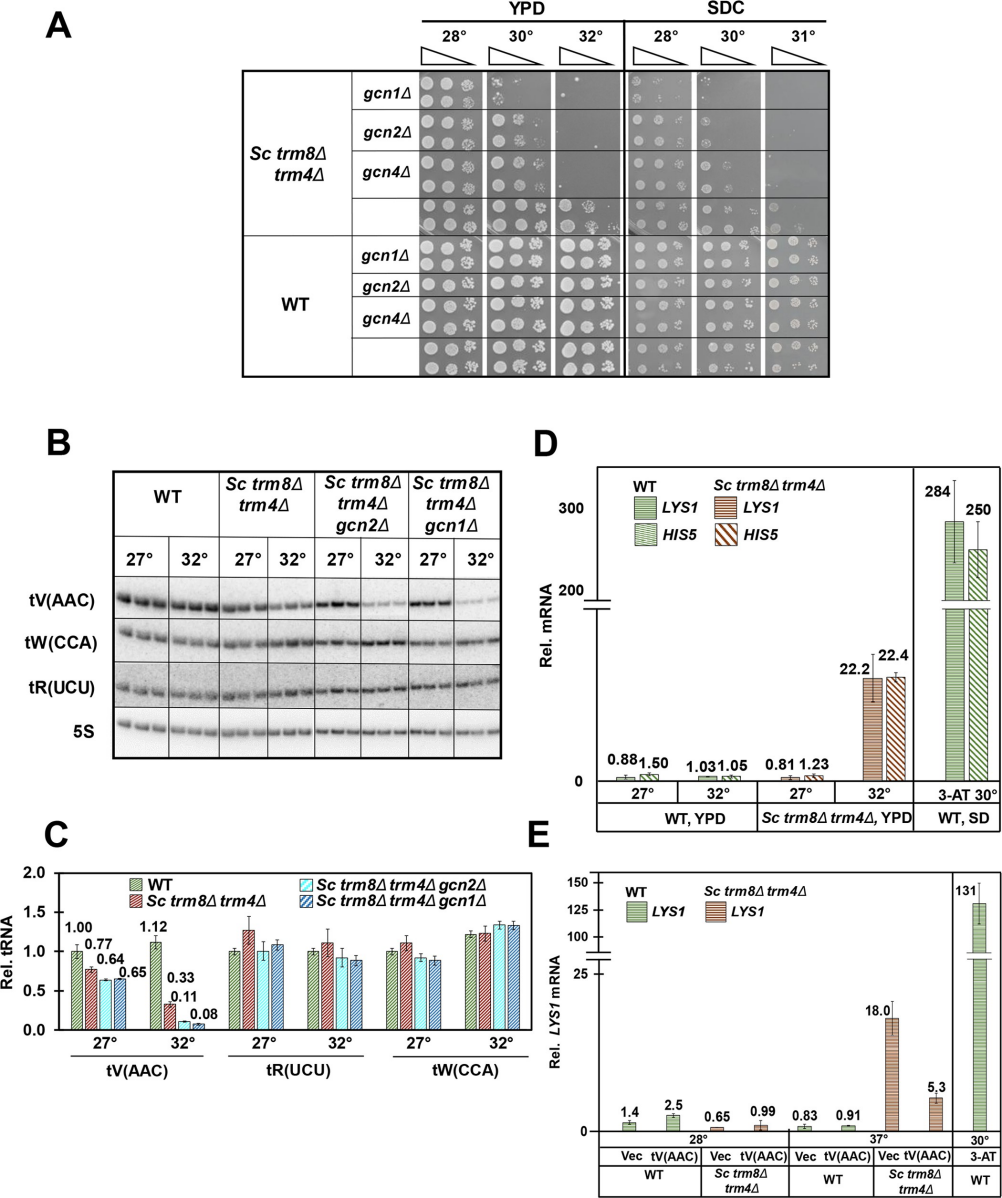
Mutations in the GAAC pathway exacerbate the temperature sensitivity of *S*. *cerevisiae trm8Δ trm4Δ* mutants as well as tV(ACC) decay. **(*A*) Deletion of *GCN1*, *GCN2*, or *GCN4* exacerbated the temperature sensitivity of *S*. *cerevisae trm8Δ trm4Δ* mutants in YPD and SDC media.** Strains were grown overnight in YPD media at 27°C and analyzed for growth on YPD and SDC plates at different temperatures. ***(B*) Deletion of *GCN1* or *GCN2* exacerbated tV(AAC) decay of *trm8Δ trm4Δ* mutants at 32°C.** Strains were grown in YPD media 27°C, shifted to 32°C and harvested after 4 hours, and then bulk RNA was isolated and analyzed by northern blotting. ***(C)* Quantification of tRNA tV(AAC), tR(UCU), and tW(CCA) levels from the northern in Fig 6B.** The bar chart depicts levels of tRNA species at 27°C or 32°C, relative to levels of that tRNA in the WT strain at 27°C (each value itself first normalized to levels of the control 5S rRNA). tRNA levels are indicated by diagonal hatching for WT (green); *S*. *cerevisiae* (*Sc*) *trm8Δ trm4Δ* (brown); *Sc trm8Δ trm4Δ gcn2Δ* (light blue); and *Sc trm8Δ trm4Δ gcn1Δ* (dark blue). ***(D) trm8Δ trm4Δ* mutants induced the GAAC pathway at 32°C.** Bulk RNA from the growth done for Fig 6B was used for RT-qPCR analysis of levels of *LYS1* and *HIS5* mRNA, normalized to *ACT1*. mRNA levels are indicated by horizontal lines for *LYS1* and hatching for *HIS5*, WT (green); *Sc trm8Δ trm4Δ* (brown). Right side: Relative levels of *LYS1* mRNA of WT cells grown at 30°C in SD-His media, and then treated with 10 mM 3-AT for 1 hour, evaluated in parallel to other samples. ***(E)* tV(AAC) overproduction repressed the GAAC induction of *trm8Δ trm4Δ* mutants at 36°C.** Strains with plasmids as indicated were grown in SD-Ura media 27°C and shifted to 36°C for 1 hour, and then RNA was isolated and relative *LYS1* mRNA levels were analyzed by RT-qPCR as described in Fig 6D. Right side: GAAC induction of WT cells grown and induced with 3-AT as described in Fig 6D.

To investigate the evolutionary implications of the GAAC pathway on RTD, we examined the consequences of deletion of GAAC components in *S*. *cerevisiae trm8Δ trm4Δ* mutants, which are highly temperature sensitive due to substantial decay of tV(AAC) by the RTD pathway, compared to the modest RTD-dependent temperature sensitivity and the limited tV(AAC) decay of *S*. *cerevisiae trm8Δ* mutants [18,39]. In contrast to our results in *S*. *pombe trm8Δ* GAAC mutants, deletion of *GCN1 or GCN2* exacerbated the temperature sensitivity of *S*. *cerevisiae trm8Δ trm4Δ* mutants in both rich (YPD) and minimal complete (SDC) media, and this exacerbated temperature sensitivity was also observed upon deletion of the GAAC transcription factor *GCN4* (Fig 6A). Moreover, we found that a *met22Δ* mutation, known to prevent RTD, reversed the enhanced temperature sensitivity of a *trm8Δ trm4Δ gcn2Δ* strain relative to a *trm8Δ trm4Δ* mutant (S20 Fig). Furthermore, a similar exacerbated temperature sensitivity due to mutation of the GAAC pathway was also observed in other *S*. *cerevisiae* modification mutants known to be subject to RTD [22,39], including *trm8Δ*, *trm1Δ*, *tan1Δ*, and *tan1Δ trm44Δ* mutants (S21 Fig).

To determine if the exacerbated growth defect of *S*. *cerevisiae trm8Δ trm4Δ* GAAC mutants was due to exacerbated loss of tV(AAC), we analyzed tRNA levels after a four-hour temperature shift from permissive to non-permissive temperature (27°C to 32°C). Consistent with the exacerbated temperature sensitivity caused by the *gcn1Δ* and *gcn2Δ* mutations, tV(AAC) levels were further reduced in both *trm8Δ trm4Δ gcn1Δ* and *trm8Δ trm4Δ gcn2Δ* mutants at 32°C, compared to the *trm8Δ trm4Δ* mutant (12% and 17% vs 43%, relative to the values at 27°C) (Fig 6B and 6C). Using a pre-tV(AAC) probe specific for 7 of the 14 tV(AAC) genes in *S*. *cerevisiae*, we found that levels of the primary pre-tV(AAC) transcript were modestly reduced at 32°C in the *trm8Δ trm4Δ gcn1Δ* mutant, relative to the *trm8Δ trm4Δ* mutant (S22 Fig). This result suggests that reduced pre-tV(AAC) transcription could be responsible for some of the exacerbated loss of tV(AAC) in the *trm8Δ trm4Δ gcn1Δ* mutant and for its exacerbated temperature sensitivity, although the magnitude of the change may not account for all of the additional loss of tV(AAC).

### The temperature sensitivity and tV(AAC) decay of *S*. *cerevisiae trm8Δ trm4Δ* mutants coincides with GAAC activation

**Fig 7:**
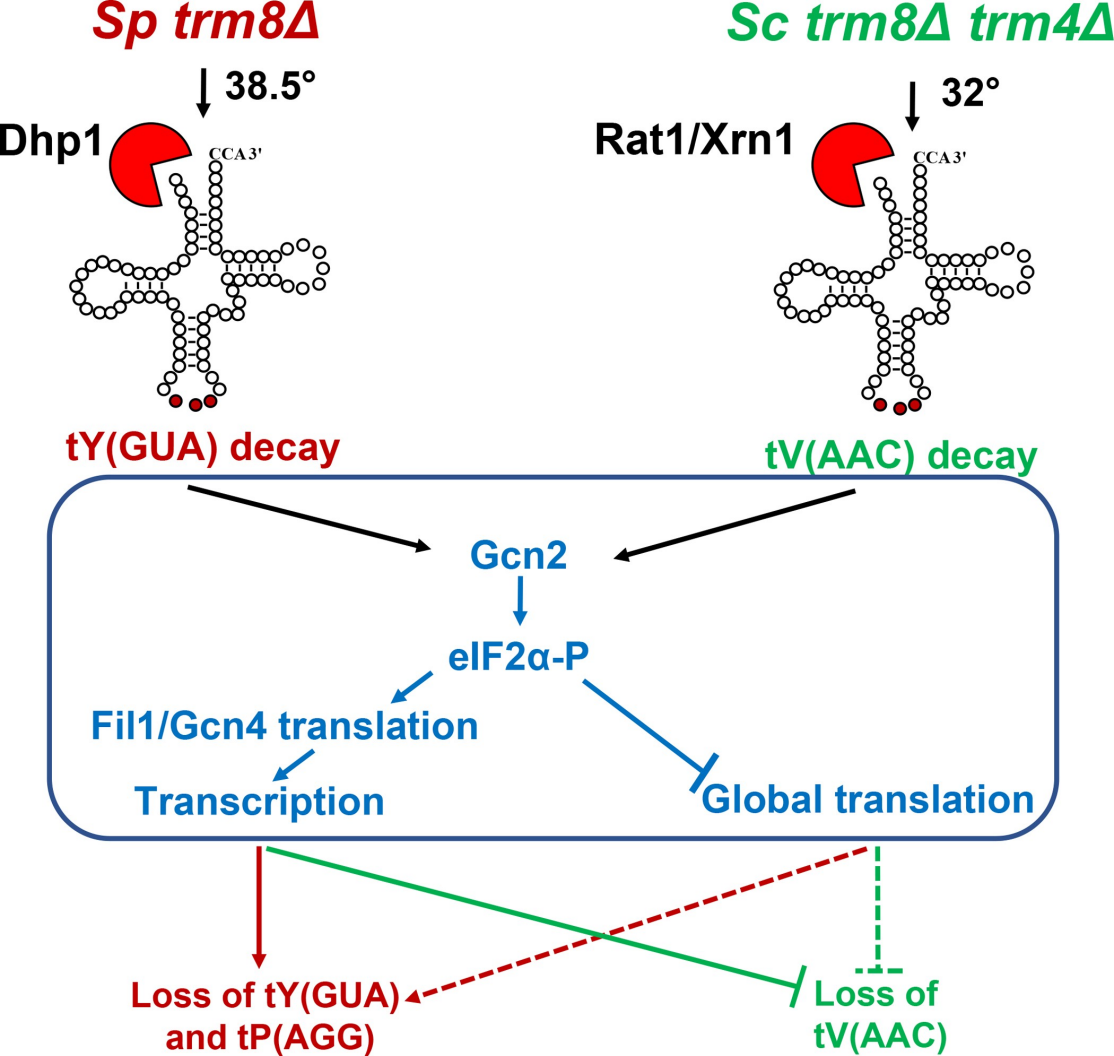
A model illustrating the interplay of the RTD pathway and GAAC induction in *S*. *pombe* and *S*. *cerevisiae*. Left: *S*. *pombe trm8Δ* mutants (red) trigger RTD of tY(GUA), leading to GAAC induction and further loss of tY(GUA) and tP(AGG). The further reduced levels of tRNA resulting from GAAC induction is in part due to transcription upregulation of Fil1 target genes (solid lines), and may also be due in part to the global reduction in translation (dotted lines). Right: *S*. *cerevisiae trm8Δ trm4Δ* mutants (green) trigger RTD of tV(AAC), leading to GAAC induction. This results in inhibition of further loss of tV(AAC), which is due in part to the the transcription upregulation of Gcn4 target genes (solid lines), and may also be due to global reduction in translation (dotted lines).

Since GAAC mutations exacerbated the RTD growth defect and enhanced the decay of an *S*. *cerevisiae trm8Δ trm4Δ* mutant at 32°C, it seemed likely that the GAAC pathway was activated in the *trm8Δ trm4Δ* mutant. To evaluate GAAC activation, we measured mRNA levels of the Gcn4 target genes *LYS1* and *HIS5* after the 4 hour temperature shift of *trm8Δ trm4Δ* mutants to 32°C, using the same RNA as in the northern analysis of decay (Figs 6B and 6C and S22). RT-qPCR analysis showed that *trm8Δ trm4Δ* mutants had a large increase in relative *LYS1* mRNA levels at 32°C, compared to 27°C (22.2 vs 0.81, 27.4-fold), or to WT cells at either 27°C or 32°C (Fig 6D), showing that the GAAC activation was specific to the *trm8Δ trm4Δ* mutant at 32°C. We observed a similar activation of the *GCN4* target *HIS5* in the *trm8Δ trm4Δ* mutant at 32°C. This GAAC activation was correlated with a modest but distinct increase in uncharged tV(AAC) commensurate with the reduced tV(AAC) levels (S23 Fig). Furthermore, we found that overproduction of tV(AAC) in *trm8Δ trm4Δ* mutants suppressed induction of the GAAC pathway (Fig 6E), showing that GAAC activation in *trm8Δ trm4Δ* mutants was due to the reduced function of tV(AAC). Thus, in both *S*. *cerevisiae trm8Δ trm4Δ* mutants and *S pombe trm8Δ* mutants, degradation of a single biologically relevant tRNA is the cause of GAAC induction, which then either promotes further loss of tRNA in *S*. *pombe*, or restores tRNA in *S*. *cerevisiae* (Fig 7).

## Discussion

The results described here provide strong evidence that the RTD pathway is conserved between the distantly related species *S*. *cerevisiae* and *S*. *pombe*. We have shown that the temperature sensitivity of *S*. *pombe trm8Δ* mutants is due to reduced levels of tY(GUA) and to some extent tP(AGG), and is efficiently suppressed by mutations in the 5'-3' exonuclease Dhp1 that concomitantly restore the levels of these tRNAs, strongly suggesting decay of tY(GUA) and tP(AGG) by the RTD pathway. As RTD is triggered in *S*. *cerevisiae* strains lacking m^2,2^G_26_ or ac^4^C_12_, as well as in strains lacking m^7^G_46_, [22,39], we speculate that the RTD pathway will also act in *S*. *pombe* strains lacking other body modifications. Furthermore, given the large evolutionary distance between *S*. *cerevisiae* and *S*. *pombe*, we speculate that the RTD pathway is conserved throughout eukaryotes. The existence of a mammalian RTD pathway could explain the reduced levels of specific tRNA species in mouse strains lacking m^5^C in their tRNAs [33,87] and in mouse embryonic stem cells lacking m^7^G in their tRNAs [46], and might explain other phenotypes associated with mutations in *METTL1* or *WDR4* [29,30,46,88].

It is puzzling that although tP(AGG) is substantially more degraded than tY(GUA) in *S*. *pombe trm8Δ* mutants at elevated temperatures, the temperature sensitivity of the mutants in both rich and minimal media is primarily due to decay of tY(GUA). One possible explanation of this result is that tY(GUA) levels might be more limiting in the cell than tP(AGG) levels at elevated temperature, relative to the number of their respective cognate codons requiring decoding. This type of argument was advanced as a possible explanation for why the growth defects of i^6^A-lacking *S*. *pombe tit1Δ* mutants were rescued by increased expression of tY(GUA), but not by any of the other four Tit1 tRNA substrates [89]. A second, and less likely, interpretation is that tP(AGG) decoding might be compensated by other tRNA^Pro^ isodecoders specific for the CCN codon box. This explanation is based on the finding that in *S*. *cerevisiae*, deletion of the two tP(AGG) genes is viable, implying that the 10 tP(UGG) isodecoders can decode all four proline CCN codons [90]. However, as *S*. *pombe* has six tP(AGG) genes and only two tP(UGG) genes (as well as one tP(CGG)), it seems unlikely that the loss of almost all of the tP(AGG) in *trm8Δ* mutants can be efficiently compensated by the small number of tP(UGG) species (assuming that tRNA expression from each gene is comparable).

It is not immediately clear why tP(AGG) and tY(GUA) are the specific tRNAs subject to RTD in *S*. *pombe trm8Δ* mutants. Based on current understanding of RTD determinants in *S*. *cerevisiae*, RTD substrate specificity is determined by stability of the stacked acceptor and T-stem, with contributions to stability from the tertiary fold that are reduced in modification mutants, and some contributions from the other two stems, but not the anticodon loop [18,22,39–42]. tP(AGG) may be an RTD substrate because it is predicted to have a less stable acceptor and T-stem than most Trm8 substrates (S1 Table) [91]. Furthermore, the destabilizing C_4_-A_69_ mismatch in the middle of the tP(AGG) acceptor stem might be expected to lead to increased local breathing at the 5' end, which is likely important for recognition by the 5'-3' exonucleases Xrn1 and Rat1, as the Xrn1 active site binds the three most 5' nucleotides [92]. However, it is more difficult to rationalize why tY(GUA) is a substrate for RTD in *trm8Δ* mutants, as its acceptor and T-stem are predicted to be moderately stable among Trm8 substrates. However, tY(GUA) does have a destabilizing N_27_-N_43_ pair (C_27_-U_43_ for 3 isodecoders, and U_27_-U_43_ for one isodecoder), which might reduce the stability of the tertiary fold by affecting stability of the adjacent tertiary 26–44 interaction [93–95].

It is not yet clear how Dhp1 degrades tY(GUA) in *S*. *pombe trm8Δ* mutants. In *S*. *cerevisiae*, Rat1 is nuclear [96] and catalyzes a substantial amount of the decay of mature tV(AAC) in *trm8Δ trm4Δ* mutants [39], suggesting that the retrograde transport pathway is required to deliver the tV(AAC) substrate to the nucleus [5,97–100]. However, we do not know the exact species of tY(GUA) that is degraded by Dhp1 in *S*. *pombe trm8Δ* mutants. If mature tY(GUA) is the actual Dhp1 substrate, it is almost certainly subject to retrograde transport back to the nucleus for the subsequent decay, because Dhp1 is known to be nuclear [64,101] and *S*. *pombe* pre-tRNA splicing initiates in the cytoplasm on the mitochondrial surface [102]. Such a retrograde transport mechanism for hypomodified tY(GUA) lacking m^7^G_46_ in *S*. *pombe trm8Δ* mutants would be similar to that shown for hypomodified tRNAs lacking m^2,2^G_26_ in *S*. *cerevisiae trm1Δ* mutants [100]. However, it is also possible that Dhp1 acts to degrade unspliced pre-tY(GUA) that accumulates in *trm8Δ* mutants, analagous to the recently described Met22-dependent pre-tRNA decay pathway [103].

It seems likely that the 5-FU sensitivity of *S*. *pombe trm8Δ* mutants is due to decay of multiple tRNA species in the presence of the drug, caused by the reduced levels of Ψ and m^5^U [48–50], in addition to the lack of m^7^G. This interpretation is consistent with the lack of suppression of the 5-FU sensitivity of *trm8Δ* mutants by tY(GUA) and tP(AGG), and its almost complete suppression by *dhp1* mutations, and is consistent with the enhanced 5-FU sensitivity of a number of tRNA body modification mutants in *S*. *cerevisiae* [61].

Our finding that loss of function of tRNA due to tRNA decay is itself the trigger for induction of the GAAC response in both *S*. *pombe trm8Δ* mutants and *S*. *cerevisiae trm8Δ trm4Δ* mutants suggests an intimate relationship between reduced tRNA function and GAAC activation. The loss of functional tRNA below some presumed threshold level is the proximal cause of GAAC induction, because in each organism the GAAC pathway is activated at the lowest temperature at which tRNA decay and a growth defect is observed, and in each organism overproduction of the physiologically relevant tRNA represses GAAC induction. The GAAC pathway has previously been implicated in the biology of a number of anticodon loop modifications [83,104,105]. Robust constitutive GAAC induction is observed in *S*. *cerevisiae* and *S*. *pombe trm7Δ* mutants (lacking Nm_32_ and Nm_34_) and *S*. *cerevisiae pus3Δ* mutants (lacking Ψ_38_ and Ψ_39_) [83,105], each of which has a constitutive growth defect [106,107], and GAAC induction is known to be Gcn2-dependent in *S*. *cerevisiae trm7Δ* mutants [83]. By contrast, *S*. *cerevisiae* mutants lacking either the mcm^5^U or the s^2^U moiety of mcm^5^s^2^U induce the GAAC pathway independently of Gcn2 at 30°C [104] and are temperature sensitive at 37°C [108]. Our finding that *S*. *pombe trm8Δ* and *S*. *cerevisiae trm8Δ trm4Δ* mutants each trigger Gcn2-dependent GAAC induction only at the temperature that the growth defect is observed is consistent with Gcn2-dependent GAAC induction in *S*. *cerevisiae* anticodon loop modification mutants with a constitutive growth defect.

It is striking that the induction of the GAAC response due to tRNA decay in *S*. *pombe trm8Δ* mutants and *S*. *cerevisiae trm8Δ trm4Δ* mutants has opposite consequences in each organism. In *S*. *pombe trm8Δ* mutants, activation of the GAAC response exacerbates the growth defect, as mutation of any of four components (*gcn1*, *gcn2*, *tif221*, *or fil1*) protects against loss of tRNA. Activation of the GAAC pathway is also part of the reason that *S*. *cerevisiae trm7Δ* mutants grow poorly [83], and defects in the integrated stress response pathway (ISR) in humans are implicated in disease phenotypes [109–111]. By contrast, in *S*. *cerevisiae trm8Δ trm4Δ* mutants, activation of the GAAC response rescues the growth defect, as deletion of any of three GAAC components *(gcn1Δ*, *gcn2Δ*, or *gcn4Δ*) exacerbates the growth defect. Furthermore, this result extends to multiple *S*. *cerevisiae* modification mutants with an RTD phenotype, since a *gcn2Δ* mutation also exacerbated the growth defects of *trm8Δ*, *trm1Δ*, *tan1Δ*, and *tan1Δ trm44Δ* mutants. Although the rescue of RTD in *S*. *cerevisiae* by GAAC activation is opposite to the exacerbating effect of GAAC activation in *S*. *pombe trm8Δ* mutants, it is consistent with a concerted stress response. Moreover, the finding that GAAC effects on RTD extended to *fil1Δ/gcn4Δ* mutations in *S*. *pombe* and *S*. *cerevisiae* has mechanistic implications. Deletion of *GCN1* or *GCN2* each prevent sensing of tRNA status, and the consequent eIF2α phosphorylation, reduced translation initiation, reduced global translation, and massive downstream transcription activation. However, as *fil1*^*+*^/Gcn4 is downstream of the sensing machinery, but upstream of the transcription activation, we infer that the GAAC effects on RTD in *S*. *pombe* and *S*. *cerevisiae* are in part due to lack of transcription activation by *fil1*^*+*^/Gcn4.

The opposite effects of GAAC activation on growth and RTD in *S*. *pombe trm8Δ* mutants and in several *S*. *cerevisiae* RTD mutants is likely due to a differential GAAC response. Although in each organism the GAAC pathway is known to regulate the transcription of more than 500 genes [75,77,79,82], there are distinct differences in the GAAC response in the two species. For example, it is known that the GAAC response to amino acid starvation results in repression of methionine synthesis genes in *S*. *pombe* but induction of these genes in *S*. *cerevisiae* [79]. As a *met22Δ* mutation is known to inhibit RTD in *S*. *cerevisiae* [22,39], this opposite GAAC activation effect on methionine genes in the two organisms is in the wrong direction to explain the opposite RTD effects of the GAAC pathway. Other possible explanations for the differential effects of the GAAC pathway on RTD include differential regulation of the synthesis or biochemical activity of RTD regulators such as EF1A, aminoacyl tRNA synthetases, pol III transcription [22,112], or 5'-3' exonucleases [39,42], as well as changes in any number of indirect effectors affecting overall levels or availability of tRNA and/or nucleases. In addition, the overall stress response pathways are substantially different between *S*. *cerevisiae* and *S*. *pombe*. In *S*. *cerevisiae*, Gcn2 is the sole eIF2α kinase regulating stress responses [78,113,114], whereas in *S*. *pombe* three different eIF2α kinases (Gcn2, Hri1 and Hri2) [115] each respond to a diverse set of stress treatments [72,74,84,85,109]. The differences in kinases affecting eIF2α phosphorylation implies substantial differences between the two species in regulation of all sorts of combinations of stress response, which might be occurring at elevated temperature when tRNA decay is occurring [116].

The results outlined here underscore that GAAC activation occurs in *S*. *cerevisiae* and *S*. *pombe trm8Δ* modification mutants precisely at the point of observed growth stress due to tRNA decay, albeit with different effects in *S*. *pombe trm8Δ* mutants and *S*. *cerevisiae trm8Δ trm4Δ* mutants. These results, coupled with the constitutive GAAC activation in *S*. *pombe* and *S*. *cerevisiae trm7Δ* mutants and *S*. *cerevisiae pus3Δ* mutants [83,105], fuel speculation that the GAAC response will also be activated in mammals and other eukaryotes with tRNA modification mutations or other mutations that result in reduced tRNA function. Given that GAAC activation at the onset of reduced tRNA function regulates RTD in opposite ways in *S*. *pombe* and *S*. *cerevisiae*, it would be interesting to determine the GAAC effect on tRNA decay and tRNA levels in mammalian systems. Based on the observation that GAAC activation in mice attenuates the growth defects caused by the combination of reduced tRNA^Arg(UCU)^ levels and a defect in the ribosome recycling component GTPBP2 [86], we speculate that GAAC activation by reduced tRNA function in mammals will likewise attenuate RTD and promote survival.

As Trm8 is phosphorylated and likely inactivated by treatment of HEK293 cells with insulin-like growth factor-1 [117], it seems plausible that if RTD is conserved in mammals, m^7^G modification dynamics could be used to regulate tRNA levels physiologically. There is growing evidence that levels of a number of modifications are under dynamic control in different conditions [118,119]. There is also evidence that regulation of tRNA expression plays an important role in differentiation and proliferation, and is also a characteristic of breast cancer [120–124]. For example, tRNA^Arg^ iso-acceptors have different expression in differentiated vs proliferating cells [123] and tR(UCU) iso-decoders show tissue specific regulation of expression [125]. It remains to be determined if regulated changes in expression, phosphorylation, or biochemical activity of METTL1 or WDR4 result in altered m^7^G levels and consequent changes in tRNA levels that physiologically regulate expression.

## Materials and methods

### Yeast strains

*S*. *pombe* haploid WT and two independent *S*. *pombe trm8Δ*::*kanMX* strains were derived from SP286 (*ade6-M210/ade6-M216*, *leu1-32/leu1-32*, *ura4-D18/ura4-D18 h+/h+*)[126], and were obtained from Dr. Jeffrey Pleiss. *S*. *pombe trm8Δ*::*kanMX* strains were also generated from haploid WT strains by PCR amplification of *trm8Δ*::*kanMX* DNA, followed by linear transformation using lithium acetate [127]. *S*. *cerevisiae* deletion strains are shown in S3 Table, and were constructed by linear transformation with PCR amplified DNA from the appropriate knockout strain [128]. All strains were confirmed before use by PCR amplification.

### Plasmids

Plasmids used in this study are listed in S4 Table. AB553-1 was constructed by insertion of a NotI restriction site between the P_*nmt1*_ promoter and the PstI site of the pREP3X plasmid. The *S*. *pombe* plasmid expressing *S*. *pombe* P_*trm8+*_ *trm8*^*+*^ (ETD 67–1) was constructed by inserting PCR amplified DNA genomic DNA (including 1000 bp upstream and 1000 bp downstream) into the NotI and XhoI sites of AB 553–1, removing the P_*nmt1*_ promoter. Plasmids expressing *S*. *pombe* P_*dhp1*_ *dhp1*^*+*^ or *S*. *pombe* tRNA genes were constructed using the same approach, including ~ 300 bp upstream and 300 bp downstream for the tRNA genes. *S*. *pombe* plasmids expressing P_nmt1**_ *gcn2*^*+*^ (low strength, no message in thiamine) or P_nmt1*_ *tif221*^*+*^ (medium strength, no message in thiamine) were constructed by PCR amplification of the respective coding sequence from *S*. *pombe* WT genomic DNA (including introns), and insertion into the XhoI and BamHI sites of the pREP81X or pREP41X vectors respectively.

### Yeast media and growth conditions

*S*. *pombe* strains were grown at desired temperatures in rich (YES) media (containing 0.5% yeast extract, 3% glucose, and supplements of 225 mg/l of adenine, uracil, leucine, histidine and lysine), or Edinburgh minimal media (EMM) containing glucose and the same supplements, as well as similar amounts of relevant auxotrophic requirements. Minimal complete (EMM-C) media was supplemented with 225 mg/l of all amino acids, adenine, and uracil, as well as 100 mg/l of para-amino benzoic acid and inositol, and 1125 mg/l of leucine for Leu^-^ auxotrophs [79]. For temperature shift experiments, cells were grown in YES or EMMC media at 30°C to OD_600_ ~ 0.5, diluted to ~ 0.1 OD in pre-warmed media at the desired temperature, grown to OD ~ 0.5, harvested at 4°C, washed with ice cold water, frozen on dry ice, and stored at -80°C. To select spontaneous suppressors of *S*. *pombe trm8Δ* mutants, cells were grown overnight in YES media at 30°C and ~10^7^ cells were plated on YES media plates at 38°C and 39°C. *S*. *cerevisiae* strains were grown in rich (YPD) media (containing 1% yeast extract, 2% peptone, 2% dextrose, and 80 mg/L adenine hemisulfate), or minimal complete (SDC) media [129] as indicated, and temperature shift experiments were performed as described for *S*. *pombe*. All experiments with measurements were performed in biological triplicate, unless otherwise noted.

### Bulk RNA preparation and northern blot analysis

For northern analysis, 2 or 3 biological replicates were grown in parallel, and then bulk RNA was isolated from ~ 3–5 OD pellets using glass beads and phenol [130] (for *S*. *pombe*) or hot phenol (for *S*. *cerevisiae*), resolved on a 10% polyacrylamide (19:1), 7M urea, 1X TBE gel, transferred to Amersham Hybond-N^+^ membrane, and analyzed by hybridization to 5’ ^32^P-labeled DNA probes (S5 Table) as described [18]. For analyzing tRNA charging levels of both *S*. *pombe* and *S*. *cerevisiae*, RNA was prepared under acidic conditions (pH 4.5), resolved on a 6.5% polyacrylamide (19:1), 8 M urea, 0.1 M sodium acetate (pH 5.0) gel at 4°C, and analyzed as described. [18].

### Quantitative RT-PCR analysis

Strains were grown in triplicate to log phase and bulk RNA was prepared from 2–5 OD pellets using acid washed glass beads and phenol. Then, RNA was treated with RQ1 RNase-free DNase (Promega), reverse transcribed with Superscript II Reverse Transcriptase, and quantitative PCR was performed on the cDNA as previously described [131].

### Isolation and purification of tRNA

*S*. *pombe* WT and *trm8Δ* mutant strains were grown to ~ 0.5 OD in YES media at 30°C. Then bulk low molecular weight RNA was extracted from ~ 300 OD of pellets by using hot phenol, and tRNAs were purified using 5'-biotinylated oligonucleotides complementary to the corresponding tRNAs (S6 Table) as previously described [132].

### HPLC analysis of nucleosides of purified tRNA

Purified tRNAs (~ 1.25 μg) were digested to nucleosides by treatment with P1 nuclease, followed by phosphotase, as previously described [132], and nucleosides were analyzed by HPLC at pH 7.0 as previously described [133].

### Whole genome sequencing

Whole genome sequencing was performed by the University of Rochester Genomics Center at a read depth of 20–110 per genome nucleotide.

### Crude extracts and western blot analysis

Crude extracts of *S*. *pombe* WT and *trm8Δ* mutants were prepared by lysis with glass beads as described [79]. Then 25 μg of crude extract proteins were resolved on 4–20% SDS-PAGE gels (Criterion TGX, Bio-Rad), transferred to a 0.2 μm nitrocellulose membrane (Bio-Rad), and probed with antibodies as described [134], using anti-phosphorylated eIF2α (Cat. # 44-728G, Thermofisher; diluted 1:6000) and anti-α-tubulin (Cat. # T-5168 Sigma, diluted 1:6000).

## Supporting information

S1 Fig *S*. *pombe trm8Δ* mutants have a temperature sensitive growth defect in liquid YES media. Strains were grown in YES media at 30°C, shifted to the indicated temperatures, and then growth was monitored for 8 hours before harvest as described in Materials and Methods, and tRNA analysis as done in Fig 2A and 2B. WT, green; *Sp trm8Δ*, brown.
(PDF)

S2 Fig *S*. *pombe trm8Δ* mutants had reduced 5S rRNA and 5.8S rRNA levels at higher temperatures. The northern blot shown in Fig 2A was used to analyze the non-Trm8 substrate tL(UAA), 5S rRNA, and 5.8S rRNA. The bar chart depicts levels of RNA species at each temperature, relative to their levels in the WT strain at 30°C (each value itself first normalized to levels of the control non-Trm8 substrate tG(GCC)). 30°C, green; 36.5°C, yellow; 37.5°C, orange; 38.5°C. red.
(PDF)

S3 Fig tP(AGG) of *S*. *pombe trm8Δ* mutants has no detectable m^7^G. *trm8Δ* mutants and WT cells were grown in YES media at 30°C and tP(AGG) was purified and analyzed for modifications as in Fig 1A.
(PDF)

S4 Fig Northern analysis of all Trm8 substrates (except tP(AGG), tY(GUA), and tT(AGU)) in *S*. *pombe trm8Δ* and WT cells after shift from 30°C to 36.5°C, 37.5°C, and 38.5°C. The northern blot shown in Fig 2A was continued to analyze levels of all other predicted Trm8 substrate tRNAs, as well as the non-Trm8 substrate tL(UAA), 5S RNA, and 5.8S RNA, in WT and *trm8Δ* mutants at different temperatures.
(PDF)

S5 Fig **A. Among 21 predicted Trm8 substrate tRNAs, only tP(AGG) and tY(GUA) had reduced levels in S. pombe trm8Δ mutants at elevated temperatures.** tRNA levels were quantified, relative to tG(GCC), as described in Fig 2B. Note that data from Fig 2B is also included here for completeness. **B. Analysis of Trm8 substrate tRNAs in WT cells at elevated temperatures.**
(PDF)

S6 Fig **A. Overproduction of tY(GUA) and tP(AGG) resulted in increased levels of the corresponding tRNAs in *S*. *pombe trm8****Δ* **mutants and WT cells.** Strains with plasmids as indicated were grown in EMMC-Leu media at 30°C and shifted to 38.5°C for 8 hours, and then RNA was isolated and analyzed by northern blotting as in Fig 2A. **B. Quantification of tRNA levels in *S*. *pombe trm8****Δ* **mutants and WT cells overproducing tY(GUA) or tP(AGG).** Quantification was done as in Fig 2B.
(PDF)

S7 Fig Overproduction of both tY(GUA) and tP(AGG) fully restored growth of *S*. *pombe trm8Δ* mutants in YES + glycerol media, but not in YES media containing 5-FU. Strains grown for Fig 2D were analyzed for growth on plates containing YES media with 3% glycerol (instead of 3% glucose) and YES media with 5-FU (30 μg/ml).
(PDF)

S8 Fig *dhp1* mutations restored growth of *S*. *pombe trm8Δ* mutants in YES + 5-FU media. Strains grown for Fig 3A were analyzed for growth on YES + 5-FU (30 μg/ml) plates.
(PDF)

S9 Fig Expression of P_*dhp1*_ *dhp1*^*+*^ restored temperature sensitive growth in the *S*. *pombe trm8 dhp1-1* mutant. WT, *trm8Δ*, and *trm8Δ dhp1-1* cells expressing P_*dhp1*_ *dhp1*^*+*^ or a vector were grown overnight in EMMC-Leu media at 30°C, and analyzed for growth.
(PDF)

S10 Fig **A. *S*. *pombe trm8Δ dhp1-3* and *trm8Δ dhp1-4* mutants also restored tY(GUA) and tP(AGG) tRNA levels at 38.5°C.** Strains were grown in YES media at 30°C and shifted to 38.5°C for 8 hours, and RNA was isolated and analyzed by northern blotting as in Fig 2A. **B. Quantification of tRNA levels in different *S*. *pombe trm8Δ dhp1* mutants.** tRNA levels were quantified as in Fig 2B. tT(AGU), brown; tP(AGG), purple; tY(GUA), gray.
(PDF)

S11 Fig Mutations in the GAAC pathway did not rescue the 5-FU sensitivity of *S*. *pombe trm8Δ* mutants, and conferred enhanced 3-AT sensitivity. Strains grown for Fig 4A were analyzed for growth on plates containing YES media + 5-FU (30 μg/ml) and EMMC-His media + 10 mM 3-AT.
(PDF)

S12 Fig Reconstructed *S*. *pombe trm8Δ gcn2Δ* mutants had the same growth properties as the original *trm8Δ gcn2-1* strain. Strains were analyzed for growth on YES media, EMMC-His or EMMC-His media containing 10 mM 3-AT, as described in Fig 1B. Reconstructed *S*. *pombe trm8Δ* mutant was labeled as V3.
(PDF)

S13 Fig **A. Northern analysis of WT, *S*. *pombe trm8Δ*, *trm8Δ gcn2-2*, *trm8Δ tif221-1*, and *trm8Δ gcn2-3* cells.** Strains were grown in YES media at 30°C and shifted to 38.5°C for 8 hours, and RNA was isolated and analyzed by northern blotting as in Fig 2A. **B. Quantification of tRNA levels.** tRNA levels were quantified as in Fig 2B. n = 2 for all strains.
(PDF)

S14 Fig Deletion of *fil1*^*+*^ partially restored growth in *S*. *pombe trm8Δ* mutants in EMMC-His media. Strains were grown overnight in YES media at 30°C and analyzed for growth.
(PDF)

S15 Fig The temperature sensitivity of *S*. *pombe trm8Δ* mutants observed at 38.5°C in YES liquid media was suppressed in a *trm8Δ gcn2-1* mutant. Strains were grown in YES media at 30°C, shifted to 36.5°C, 37.5°C, and 38.5°C as indicated, and then growth was monitored for 8 hours before harvest as described in Materials and Methods, and analysis of mRNAs and tRNAs in Figs 5A–5C and S16 and S19.
(PDF)

S16 Fig *S*. *pombe trm8Δ* mutants induced *aro8*^*+*^ mRNA expression at 38.5°C, but not at 37.5°C. Bulk RNA from the growth in S15 Fig was used for the RT-qPCR analysis of *aro8*^*+*^*(SPAC56E4*.*03)* mRNA levels, as in Fig 5A.
(PDF)

S17 Fig **A. *S*. *pombe trm8Δ* mutants had increased levels of phophorylated eIF2α at 38.5°C** WT and *S*. *pombe trm8Δ* strains were grown in YES media at 30°C, shifted to 38.5°C for 8 hours and cells were harvested. Then crude extracts were prepared and analyzed by western blotting as described in Materials and Methods, using anti-phosphorylated eIF2α and anti-α-tubulin. Controls: WT and *gcn2Δ* mutants were grown at 30°C in EMMC-His media and, where indicated, treated with 20 mM 3-AT. Then extracts were prepared and evaluated by blotting in parallel to the experimental samples. **B. Increased levels of phophorylated eIF2α in *S*. *pombe trm8Δ* mutants at 38.5°C were associated with increased expression of *lys4***^***+***^ **and *aro8***^***+***^ **mRNAs**. Bulk RNA was prepared from the growth done for S17A Fig, and levels of *aro8*^*+*^*(SPAC56E4*.*03)* and *lys4*^*+*^ mRNAs were quantified relative to *act1*^*+*^, using RT-qPCR, as in Fig 5A.
(PDF)

S18 Fig **A. Analysis of charging levels of tY(GUA) or tP(AGG) in *S*. *pombe trm8Δ* mutants at 38.5°C.** Strains were grown in YES media at 30°C and shifted to 38.5°C, and samples were harvested after 8 hours. Then bulk RNA was isolated and resolved by denaturing PAGE under acidic conditions (to preserve tRNA charging), transferred, and then analyzed by hybridization as described in Materials and Methods. Control samples (WT and *S*. *pombe trm8Δ* mutants) were treated with 1 mM EDTA and 0.1 M Tris-HCl (pH 9.0) for 30 min at 37°C to de-acylate the tRNA. b, base treated bulk RNA; Upper arrows, charged tRNA species; lower arrows with dashed lines, uncharged tRNA species. **B. Quantification of tY(GUA) or tP(AGG) charging and tRNA levels.** The percent charging was calculated as the ratio of aminoacylated species to the total for each tRNA. Relative levels of tP(AGG) and tY(GUA) were quantified as in Fig 2B, relative to the non-Trm8 substrate tL(UAA).
(PDF)

S19 Fig **A. Northern analysis of pre-tY(GUA) levels in *S*. *pombe* WT, *trm8Δ*, and *trm8Δ gcn2-1* mutants.** The northern blot shown in Fig 5B was continued to analyze levels of pre-tY(GUA) species in WT and *trm8Δ*, and *trm8Δ gcn2-1* mutants at different temperatures, using appropriate gene-specific probes (S5 Table) for the introns of the different tY(GUA) genes [59]. Cartoons at the right indicate exons, heavy bars; 5' leaders, 3' trailers, and introns, light bars. The primary pre-tY(GUA) transcript has 5' leader, 3' trailer, and intron, and the end-matured pre-tY(GUA) has only the intron. **B. Quantification of pre-tY(GUA) transcript levels in *S*. *pombe trm8Δ*, and *trm8Δ gcn2-1* mutants, from northern in S19A Fig.** The primary pre-tY(GUA) transcript levels were normalized to levels of tG(GCC).
(PDF)

S20 Fig Deletion of *MET22* restored growth of *S*. *cerevisiae trm8Δ trm4Δ gcn2Δ* mutants at elevated temperatures. Strains were grown overnight in YPD media 28°C and analyzed for growth on YPD plates.
(PDF)

S21 Fig **A. Deletion of *GCN2* exacerbated the temperature sensitivity of *S*. *cerevisiae trm8Δ* and *trm1Δ* mutants in SDC media.** Strains were grown overnight in YPD media at 28°C and analyzed for growth on SDC plates. **B. Deletion of *GCN2* exacerbated the temperature sensitivity of *S*. *cerevisiae tan1Δ* and *tan1Δ trm44Δ* mutants in SDC media.** Strains were grown overnight in YPD media 28°C and analyzed for growth on SDC plates.
(PDF)

S22 Fig **A. Northern analysis of pre-tV(AAC) levels in WT, *S*. *cerevisiae trm8Δ trm4Δ*, and *trm8Δ trm4Δ gcn1Δ* cells after shift from 28°C to 32°C.** Bulk RNA from the growth done for Fig 6B and 6C was used for the northern analysis. **B. Quantification of the levels of the primary pre-tV(AAC) transcript.** pre-tV(AAC) levels were determined by hybridization with oligomer TDZ 415, specific for seven of the fourteen pre-tV(AAC) species, and then quantification of the upper band, corresponding to the primary transcript, with 5' leader and 3' trailer. Levels were normalized to 5S rRNA.
(PDF)

S23 Fig **A. Analysis of tRNA charging levels in *S*. *cerevisiae trm8Δ trm4Δ* mutants after shift to 32°C.** Cell pellets from the growth for Fig 6B were used to isolate acidic RNA and analyzed by acidic northern as described in S18 Fig. **B. Quantification of tRNA charging and tRNA levels.** The percent aminoacylation of tV(AAC) and tK(UUU) was calculated as described in S18 Fig, and relative tRNA levels were quantified as in Fig 6C.
(PDF)

S1 Table Variable loop sequences and the folding free energies of the acceptor stem/Tstem loop of predicted Trm8 substrate tRNAs of *S*. *pombe trm8Δ* mutants. (PDF)

S2 Table GAAC mutations identified in *S*. *pombe trm8Δ* suppressors. (PDF)

S3 Table *S*. *cerevisiae* strains used in this study. (PDF)

S4 Table Plasmids used in this study. (PDF)

S5 Table Oligomers used for northern analysis. (PDF)

S6 Table Oligomers used for tRNA purifications. (PDF)

## References

[1] Phizicky EM, Hopper AK. tRNA biology charges to the front. Genes Dev. 2010;24(17):1832–60. 10.1101/gad.1956510 20810645 PMC2932967

[2] Ramos J, Fu D. The emerging impact of tRNA modifications in the brain and nervous system. Biochim Biophys Acta Gene Regul Mech. 2019;1862(3):412–28. 10.1016/j.bbagrm.2018.11.007 30529455

[3] Boccaletto P, Machnicka MA, Purta E, Piatkowski P, Baginski B, Wirecki TK, et al MODOMICS: a database of RNA modification pathways. 2017 update. Nucleic Acids Res. 2018;46(D1):D303–D7. 10.1093/nar/gkx1030 29106616 PMC5753262

[4] Pereira M, Francisco S, Varanda AS, Santos M, Santos MAS, Soares AR. Impact of tRNA Modifications and tRNA-Modifying Enzymes on Proteostasis and Human Disease. Int J Mol Sci. 2018;19(12). 10.3390/ijms19123738 PMC6321623 30477220

[5] Hopper AK. Transfer RNA post-transcriptional processing, turnover, and subcellular dynamics in the yeast *Saccharomyces cerevisiae*. Genetics. 2013;194(1):43–67. 10.1534/genetics.112.147470 23633143 PMC3632480

[6] Urbonavicius J, Qian O, Durand JMB, Hagervall TG, Bjork GR. Improvement of reading frame maintenance is a common function for several tRNA modifications. EMBO J. 2001;20(17):4863–73. 10.1093/emboj/20.17.4863 11532950 PMC125605

[7] Lecointe F, Namy O, Hatin I, Simos G, Rousset JP, Grosjean H. Lack of pseudouridine 38/39 in the anticodon arm of yeast cytoplasmic tRNA decreases in vivo recoding efficiency. J Biol Chem. 2002;277(34):30445–53. 10.1074/jbc.M203456200 12058040

[8] Waas WF, de Crecy-Lagard V, Schimmel P. Discovery of a gene family critical to wyosine base formation in a subset of phenylalanine-specific transfer RNAs. J Biol Chem. 2005;280(45):37616–22. 10.1074/jbc.M506939200 16162496

[9] El Yacoubi B, Hatin I, Deutsch C, Kahveci T, Rousset JP, Iwata-Reuyl D, et al A role for the universal Kae1/Qri7/YgjD (COG0533) family in tRNA modification. EMBO J. 2011;30(5):882–93. 10.1038/emboj.2010.363 21285948 PMC3049207

[10] Gerber AP, Keller W. An adenosine deaminase that generates inosine at the wobble position of tRNAs. Science. 1999;286(5442):1146–9. 10.1126/science.286.5442.1146 10550050

[11] Murphy FVt, Ramakrishnan V. Structure of a purine-purine wobble base pair in the decoding center of the ribosome. Nat Struct Mol Biol. 2004;11(12):1251–2. 10.1038/nsmb866 15558050

[12] Bjork GR, Huang B, Persson OP, Bystrom AS. A conserved modified wobble nucleoside (mcm5s2U) in lysyl-tRNA is required for viability in yeast. RNA. 2007;13(8):1245–55. 10.1261/rna.558707 17592039 PMC1924908

[13] Weixlbaumer A, Murphy FVt, Dziergowska A, Malkiewicz A, Vendeix FA, Agris PF, et al Mechanism for expanding the decoding capacity of transfer RNAs by modification of uridines. Nat Struct Mol Biol. 2007;14(6):498–502. 10.1038/nsmb1242 17496902 PMC2816034

[14] Muramatsu T, Nishikawa K, Nemoto F, Kuchino Y, Nishimura S, Miyazawa T, et al Codon and amino-acid specificities of a transfer RNA are both converted by a single post-transcriptional modification. Nature. 1988;336(6195):179–81. 10.1038/336179a0 3054566

[15] Putz J, Florentz C, Benseler F, Giege R. A single methyl group prevents the mischarging of a tRNA. Nat Struct Biol. 1994;1(9):580–2. 10.1038/nsb0994-580 7634096

[16] Helm M, Giege R, Florentz C. A Watson-Crick base-pair-disrupting methyl group (m1A9) is sufficient for cloverleaf folding of human mitochondrial tRNALys. Biochemistry. 1999;38(40):13338–46. 10.1021/bi991061g 10529209

[17] Kadaba S, Krueger A, Trice T, Krecic AM, Hinnebusch AG, Anderson J. Nuclear surveillance and degradation of hypomodified initiator tRNAMet in *S*. *cerevisiae*. Genes Dev. 2004;18(11):1227–40. 10.1101/gad.1183804 15145828 PMC420349

[18] Alexandrov A, Chernyakov I, Gu W, Hiley SL, Hughes TR, Grayhack EJ, et al Rapid tRNA decay can result from lack of nonessential modifications. Mol Cell. 2006;21(1):87–96. 10.1016/j.molcel.2005.10.036 16387656

[19] Anderson J, Phan L, Cuesta R, Carlson BA, Pak M, Asano K, et al The essential Gcd10p-Gcd14p nuclear complex is required for 1-methyladenosine modification and maturation of initiator methionyl-tRNA. Genes Dev. 1998;12(23):3650–62. 10.1101/gad.12.23.3650 9851972 PMC317256

[20] Alexandrov A, Grayhack EJ, Phizicky EM. tRNA m7G methyltransferase Trm8p/Trm82p: evidence linking activity to a growth phenotype and implicating Trm82p in maintaining levels of active Trm8p. RNA. 2005;11(5):821–30. 10.1261/rna.2030705 15811913 PMC1370766

[21] Kotelawala L, Grayhack EJ, Phizicky EM. Identification of yeast tRNA Um(44) 2'-O-methyltransferase (Trm44) and demonstration of a Trm44 role in sustaining levels of specific tRNA(Ser) species. RNA. 2008;14(1):158–69. 10.1261/rna.811008 18025252 PMC2151035

[22] Dewe JM, Whipple JM, Chernyakov I, Jaramillo LN, Phizicky EM. The yeast rapid tRNA decay pathway competes with elongation factor 1A for substrate tRNAs and acts on tRNAs lacking one or more of several modifications. RNA. 2012;18(10):1886–96. 10.1261/rna.033654.112 22895820 PMC3446711

[23] Gillis D, Krishnamohan A, Yaacov B, Shaag A, Jackman JE, Elpeleg O. TRMT10A dysfunction is associated with abnormalities in glucose homeostasis, short stature and microcephaly. J Med Genet. 2014;51(9):581–6. 10.1136/jmedgenet-2014-102282 25053765

[24] Cosentino C, Toivonen S, Diaz Villamil E, Atta M, Ravanat JL, Demine S, et al Pancreatic beta-cell tRNA hypomethylation and fragmentation link TRMT10A deficiency with diabetes. Nucleic Acids Res. 2018;46(19):10302–18. 10.1093/nar/gky839 30247717 PMC6212784

[25] Najmabadi H, Hu H, Garshasbi M, Zemojtel T, Abedini SS, Chen W, et al Deep sequencing reveals 50 novel genes for recessive cognitive disorders. Nature. 2011;478(7367):57–63. 10.1038/nature10423 21937992

[26] Davarniya B, Hu H, Kahrizi K, Musante L, Fattahi Z, Hosseini M, et al The Role of a Novel TRMT1 Gene Mutation and Rare GRM1 Gene Defect in Intellectual Disability in Two Azeri Families. PloS One. 2015;10(8):e0129631 10.1371/journal.pone.0129631 26308914 PMC4550366

[27] Zhang K, Lentini JM, Prevost CT, Hashem MO, Alkuraya FS, Fu D. An intellectual disability-associated missense variant in *TRMT1* impairs tRNA modification and reconstitution of enzymatic activity. Human Mutation. 2020;41(3):600–7. 10.1002/humu.23976 31898845 PMC7981843

[28] Dewe JM, Fuller BL, Lentini JM, Kellner SM, Fu D. TRMT1-Catalyzed tRNA Modifications Are Required for Redox Homeostasis To Ensure Proper Cellular Proliferation and Oxidative Stress Survival. Mol Cell Biol. 2017;37(21). 10.1128/MCB.00214-17 PMC5640816 28784718

[29] Shaheen R, Abdel-Salam GM, Guy MP, Alomar R, Abdel-Hamid MS, Afifi HH, et al Mutation in WDR4 impairs tRNA m(7)G46 methylation and causes a distinct form of microcephalic primordial dwarfism. Genome Biology. 2015;16(1):210. 26416026 10.1186/s13059-015-0779-x PMC4587777

[30] Chen X, Gao Y, Yang L, Wu B, Dong X, Liu B, et al Speech and language delay in a patient with *WDR4* mutations. Eur J Med Genet. 2018;61(8):468–72. 10.1016/j.ejmg.2018.03.007 29597095

[31] Trimouille A, Lasseaux E, Barat P, Deiller C, Drunat S, Rooryck C, et al Further delineation of the phenotype caused by biallelic variants in the *WDR4* gene. Clinical Genetics. 2018;93(2):374–7. 10.1111/cge.13074 28617965

[32] Martinez FJ, Lee JH, Lee JE, Blanco S, Nickerson E, Gabriel S, et al Whole exome sequencing identifies a splicing mutation in *NSUN2* as a cause of a Dubowitz-like syndrome. J Med Genet. 2012;49(6):380–5. 10.1136/jmedgenet-2011-100686 22577224 PMC4771841

[33] Tuorto F, Liebers R, Musch T, Schaefer M, Hofmann S, Kellner S, et al RNA cytosine methylation by Dnmt2 and NSun2 promotes tRNA stability and protein synthesis. Nat Struct Mol Biol. 2012;19(9):900–5. 10.1038/nsmb.2357 22885326

[34] Abbasi-Moheb L, Mertel S, Gonsior M, Nouri-Vahid L, Kahrizi K, Cirak S, et al Mutations in *NSUN2* cause autosomal-recessive intellectual disability. Am J Hum Genet. 2012;90(5):847–55. 10.1016/j.ajhg.2012.03.021 22541559 PMC3376487

[35] Kadaba S, Wang X, Anderson JT. Nuclear RNA surveillance in Saccharomyces cerevisiae: Trf4p-dependent polyadenylation of nascent hypomethylated tRNA and an aberrant form of 5S rRNA. RNA. 2006;12(3):508–21. 10.1261/rna.2305406 16431988 PMC1383588

[36] LaCava J, Houseley J, Saveanu C, Petfalski E, Thompson E, Jacquier A, et al RNA degradation by the exosome is promoted by a nuclear polyadenylation complex. Cell. 2005;121(5):713–24. 10.1016/j.cell.2005.04.029 15935758

[37] Vanacova S, Wolf J, Martin G, Blank D, Dettwiler S, Friedlein A, et al A new yeast poly(A) polymerase complex involved in RNA quality control. PLoS Biol. 2005;3(6):e189 10.1371/journal.pbio.0030189 15828860 PMC1079787

[38] Gudipati RK, Xu Z, Lebreton A, Seraphin B, Steinmetz LM, Jacquier A, et al Extensive degradation of RNA precursors by the exosome in wild-type cells. Mol Cell. 2012;48(3):409–21. 10.1016/j.molcel.2012.08.018 23000176 PMC3496076

[39] Chernyakov I, Whipple JM, Kotelawala L, Grayhack EJ, Phizicky EM. Degradation of several hypomodified mature tRNA species in Saccharomyces cerevisiae is mediated by Met22 and the 5'-3' exonucleases Rat1 and Xrn1. Genes Dev. 2008;22(10):1369–80. 10.1101/gad.1654308 18443146 PMC2377191

[40] Whipple JM, Lane EA, Chernyakov I, D'Silva S, Phizicky EM. The yeast rapid tRNA decay pathway primarily monitors the structural integrity of the acceptor and T-stems of mature tRNA. Genes Dev. 2011;25(11):1173–84. 10.1101/gad.2050711 21632824 PMC3110955

[41] Guy MP, Young DL, Payea MJ, Zhang X, Kon Y, Dean KM, et al Identification of the determinants of tRNA function and susceptibility to rapid tRNA decay by high-throughput in vivo analysis. Genes Dev. 2014;28(15):1721–32. 10.1101/gad.245936.114 25085423 PMC4117946

[42] Payea MJ, Sloma MF, Kon Y, Young DL, Guy MP, Zhang X, et al Widespread temperature sensitivity and tRNA decay due to mutations in a yeast tRNA. RNA. 2018;24(3):410–22. 10.1261/rna.064642.117 29259051 PMC5824359

[43] Murguia JR, Belles JM, Serrano R. The yeast HAL2 nucleotidase is an in vivo target of salt toxicity. J Biol Chem. 1996;271(46):29029–33. 10.1074/jbc.271.46.29029 8910555

[44] Dichtl B, Stevens A, Tollervey D. Lithium toxicity in yeast is due to the inhibition of RNA processing enzymes. EMBO J. 1997;16(23):7184–95. 10.1093/emboj/16.23.7184 9384595 PMC1170319

[45] Yun JS, Yoon JH, Choi YJ, Son YJ, Kim S, Tong L, et al Molecular mechanism for the inhibition of DXO by adenosine 3',5'-bisphosphate. Biochem Biophys Res Comm. 2018;504(1):89–95. 10.1016/j.bbrc.2018.08.135 30180947 PMC6145842

[46] Lin S, Liu Q, Lelyveld VS, Choe J, Szostak JW, Gregory RI. Mettl1/Wdr4-Mediated m(7)G tRNA Methylome Is Required for Normal mRNA Translation and Embryonic Stem Cell Self-Renewal and Differentiation. Mol Cell. 2018;71(2):244–55 e5. 10.1016/j.molcel.2018.06.001 PMC6086580 29983320

[47] Okamoto M, Fujiwara M, Hori M, Okada K, Yazama F, Konishi H, et al tRNA Modifying Enzymes, NSUN2 and METTL1, Determine Sensitivity to 5-Fluorouracil in HeLa Cells. PLoS Genetics. 2014;10(9):e1004639 10.1371/journal.pgen.1004639 25233213 PMC4169382

[48] Frendewey DA, Kladianos DM, Moore VG, Kaiser II. Loss of tRNA 5-methyluridine methyltransferase and pseudouridine synthetase activities in 5-fluorouracil and 1-(tetrahydro-2-furanyl)-5-fluorouracil (ftorafur)-treated Escherichia coli. Biochim Biophys Acta. 1982;697(1):31–40. 10.1016/0167-4781(82)90042-2 6805514

[49] Santi DV, Hardy LW. Catalytic mechanism and inhibition of tRNA (uracil-5-)methyltransferase: evidence for covalent catalysis. Biochemistry. 1987;26(26):8599–606. 10.1021/bi00400a016 3327525

[50] Huang L, Pookanjanatavip M, Gu X, Santi DV. A conserved aspartate of tRNA pseudouridine synthase is essential for activity and a probable nucleophilic catalyst. Biochemistry. 1998;37(1):344–51. 10.1021/bi971874+ 9425056

[51] Watanabe K, Miyagawa R, Tomikawa C, Mizuno R, Takahashi A, Hori H, et al Degradation of initiator tRNAMet by Xrn1/2 via its accumulation in the nucleus of heat-treated HeLa cells. Nucleic Acids Res. 2013;41(8):4671–85. 10.1093/nar/gkt153 23471000 PMC3632136

[52] Parfrey LW, Lahr DJ, Knoll AH, Katz LA. Estimating the timing of early eukaryotic diversification with multigene molecular clocks. Proc Natl Acad Sci U S A. 2011;108(33):13624–9. 10.1073/pnas.1110633108 21810989 PMC3158185

[53] Alexandrov A, Martzen MR, Phizicky EM. Two proteins that form a complex are required for 7-methylguanosine modification of yeast tRNA. RNA. 2002;8(10):1253–66. 10.1017/s1355838202024019 12403464 PMC1370335

[54] Leulliot N, Chaillet M, Durand D, Ulryck N, Blondeau K, van Tilbeurgh H. Structure of the yeast tRNA m7G methylation complex. Structure. 2008;16(1):52–61. 10.1016/j.str.2007.10.025 18184583

[55] Pandolfini L, Barbieri I, Bannister AJ, Hendrick A, Andrews B, Webster N, et al METTL1 Promotes let-7 MicroRNA Processing via m7G Methylation. Mol Cell. 2019;74(6):1278–90 e9. 10.1016/j.molcel.2019.03.040 PMC6591002 31031083

[56] Zhang LS, Liu C, Ma H, Dai Q, Sun HL, Luo G, et al Transcriptome-wide Mapping of Internal N(7)-Methylguanosine Methylome in Mammalian mRNA. Mol Cell. 2019;74(6):1304–16 e8. 10.1016/j.molcel.2019.03.036 PMC6588483 31031084

[57] McCutchan T, Silverman S, Kohli J, Soll D. Nucleotide sequence of phenylalanine transfer RNA from *Schizosaccharomyces pombe*: implications for transfer RNA recognition by yeast phenylalanyl-tRNA synthetase. Biochemistry. 1978;17(9):1622–8. 10.1021/bi00602a007 247991

[58] Vogeli G. The nucleotide sequence of tRNA tyrosine from the fission yeast *Schizosaccharomyces pombe*. Nucleic Acids Res. 1979;7(4):1059–65. 10.1093/nar/7.4.1059 116193 PMC342282

[59] Chan PP, Lowe TM. GtRNAdb 2.0: an expanded database of transfer RNA genes identified in complete and draft genomes. Nucleic Acids Res. 2016;44(D1):D184–9. 10.1093/nar/gkv1309 26673694 PMC4702915

[60] Matsumoto K, Toyooka T, Tomikawa C, Ochi A, Takano Y, Takayanagi N, et al RNA recognition mechanism of eukaryote tRNA (m7G46) methyltransferase (Trm8-Trm82 complex). FEBS Lett. 2007;581(8):1599–604. 10.1016/j.febslet.2007.03.023 17382321

[61] Gustavsson M, Ronne H. Evidence that tRNA modifying enzymes are important in vivo targets for 5-fluorouracil in yeast. RNA. 2008;14(4):666–74. 10.1261/rna.966208 18314501 PMC2271368

[62] Mojardin L, Botet J, Moreno S, Salas M. Chromosome segregation and organization are targets of 5'-Fluorouracil in eukaryotic cells. Cell Cycle. 2015;14(2):206–18. 10.4161/15384101.2014.974425 25483073 PMC4352961

[63] Sugano S, Shobuike T, Takeda T, Sugino A, Ikeda H. Molecular analysis of the *dhp1+* gene of *Schizosaccharomyces pombe*: an essential gene that has homology to the *DST2* and *RAT1* genes of *Saccharomyces cerevisiae*. Mol Gen Genet. 1994;243(1):1–8. 10.1007/BF00283869 8190062

[64] Shobuike T, Tatebayashi K, Tani T, Sugano S, Ikeda H. The dhp1(+) gene, encoding a putative nuclear 5'—>3' exoribonuclease, is required for proper chromosome segregation in fission yeast. Nucleic Acids Res. 2001;29(6):1326–33. 10.1093/nar/29.6.1326 11238999 PMC29750

[65] Xiang S, Cooper-Morgan A, Jiao X, Kiledjian M, Manley JL, Tong L. Structure and function of the 5'—>3' exoribonuclease Rat1 and its activating partner Rai1. Nature. 2009;458(7239):784–8. 10.1038/nature07731 19194460 PMC2739979

[66] Dever TE, Feng L, Wek RC, Cigan AM, Donahue TF, Hinnebusch AG. Phosphorylation of initiation factor 2 alpha by protein kinase GCN2 mediates gene-specific translational control of GCN4 in yeast. Cell. 1992;68(3):585–96. 10.1016/0092-8674(92)90193-g 1739968

[67] Sattlegger E, Hinnebusch AG. Separate domains in GCN1 for binding protein kinase GCN2 and ribosomes are required for GCN2 activation in amino acid-starved cells. EMBO J. 2000;19(23):6622–33. 10.1093/emboj/19.23.6622 11101534 PMC305848

[68] Pavitt GD, Yang W, Hinnebusch AG. Homologous segments in three subunits of the guanine nucleotide exchange factor eIF2B mediate translational regulation by phosphorylation of eIF2. Mol Cell Biol. 1997;17(3):1298–313. 10.1128/mcb.17.3.1298 9032257 PMC231855

[69] Castilho BA, Shanmugam R, Silva RC, Ramesh R, Himme BM, Sattlegger E. Keeping the eIF2 alpha kinase Gcn2 in check. Biochim Biophys Acta. 2014;1843(9):1948–68. 10.1016/j.bbamcr.2014.04.006 24732012

[70] Marton MJ, Vazquez de Aldana CR, Qiu H, Chakraburtty K, Hinnebusch AG. Evidence that GCN1 and GCN20, translational regulators of GCN4, function on elongating ribosomes in activation of eIF2alpha kinase GCN2. Mol Cell Biol. 1997;17(8):4474–89. 10.1128/mcb.17.8.4474 9234705 PMC232301

[71] Sood R, Porter AC, Olsen DA, Cavener DR, Wek RC. A mammalian homologue of GCN2 protein kinase important for translational control by phosphorylation of eukaryotic initiation factor-2alpha. Genetics. 2000;154(2):787–801. 10655230 10.1093/genetics/154.2.787 PMC1460965

[72] Zhan K, Vattem KM, Bauer BN, Dever TE, Chen JJ, Wek RC. Phosphorylation of eukaryotic initiation factor 2 by heme-regulated inhibitor kinase-related protein kinases in *Schizosaccharomyces pombe* is important for fesistance to environmental stresses. Mol Cell Biol. 2002;22(20):7134–46. 10.1128/mcb.22.20.7134-7146.2002 12242291 PMC139816

[73] Elsby R, Heiber JF, Reid P, Kimball SR, Pavitt GD, Barber GN. The alpha subunit of eukaryotic initiation factor 2B (eIF2B) is required for eIF2-mediated translational suppression of vesicular stomatitis virus. J Virol. 2011;85(19):9716–25. 10.1128/JVI.05146-11 21795329 PMC3196436

[74] Anda S, Zach R, Grallert B. Activation of Gcn2 in response to different stresses. PloS One. 2017;12(8):e0182143 10.1371/journal.pone.0182143 28771613 PMC5542535

[75] Hinnebusch AG. Evidence for translational regulation of the activator of general amino acid control in yeast. Proc Natl Acad Sci U S A. 1984;81(20):6442–46. 10.1073/pnas.81.20.6442 6387704 PMC391940

[76] Dong J, Qiu H, Garcia-Barrio M, Anderson J, Hinnebusch AG. Uncharged tRNA activates GCN2 by displacing the protein kinase moiety from a bipartite tRNA-binding domain. Mol Cell. 2000;6(2):269–79. 10.1016/s1097-2765(00)00028-9 10983975

[77] Natarajan K, Meyer MR, Jackson BM, Slade D, Roberts C, Hinnebusch AG, et al Transcriptional profiling shows that Gcn4p is a master regulator of gene expression during amino acid starvation in yeast. Mol Cell Biol. 2001;21(13):4347–68. 10.1128/MCB.21.13.4347-4368.2001 11390663 PMC87095

[78] Hinnebusch AG, Natarajan K. Gcn4p, a master regulator of gene expression, is controlled at multiple levels by diverse signals of starvation and stress. Eukaryot Cell. 2002;1(1):22–32. 10.1128/ec.01.1.22-32.2002 12455968 PMC118051

[79] Udagawa T, Nemoto N, Wilkinson CR, Narashimhan J, Jiang L, Watt S, et al Int6/eIF3e promotes general translation and Atf1 abundance to modulate Sty1 MAPK-dependent stress response in fission yeast. J Biol Chem. 2008;283(32):22063–75. 10.1074/jbc.M710017200 18502752 PMC2494926

[80] Hinnebusch AG. Translational regulation of GCN4 and the general amino acid control of yeast. Annu Rev Microbiol. 2005;59:407–50. 10.1146/annurev.micro.59.031805.133833 16153175

[81] Moehle CM, Hinnebusch AG. Association of RAP1 binding sites with stringent control of ribosomal protein gene transcription in *Saccharomyces cerevisiae*. Mol Cell Biol. 1991;11(5):2723–35. 10.1128/mcb.11.5.2723 2017175 PMC360042

[82] Duncan CDS, Rodriguez-Lopez M, Ruis P, Bahler J, Mata J. General amino acid control in fission yeast is regulated by a nonconserved transcription factor, with functions analogous to Gcn4/Atf4. Proc Natl Acad Sci U S A. 2018;115(8):E1829–E38. 10.1073/pnas.1713991115 29432178 PMC5828588

[83] Han L, Guy MP, Kon Y, Phizicky EM. Lack of 2'-O-methylation in the tRNA anticodon loop of two phylogenetically distant yeast species activates the general amino acid control pathway. PLoS Genetics. 2018;14(3):e1007288 10.1371/journal.pgen.1007288 29596413 PMC5892943

[84] Dunand-Sauthier I, Walker CA, Narasimhan J, Pearce AK, Wek RC, Humphrey TC. Stress-activated protein kinase pathway functions to support protein synthesis and translational adaptation in response to environmental stress in fission yeast. Eukaryot Cell. 2005;4(11):1785–93. 10.1128/EC.4.11.1785-1793.2005 16278445 PMC1287851

[85] Martin R, Berlanga JJ, de Haro C. New roles of the fission yeast eIF2alpha kinases Hri1 and Gcn2 in response to nutritional stress. J Cell Sci. 2013;126(Pt 14):3010–20. 10.1242/jcs.118067 23687372

[86] Ishimura R, Nagy G, Dotu I, Chuang JH, Ackerman SL. Activation of GCN2 kinase by ribosome stalling links translation elongation with translation initiation. Elife. 2016;5. 10.7554/eLife.14295 PMC4917338 27085088

[87] Hussain S, Tuorto F, Menon S, Blanco S, Cox C, Flores JV, et al The mouse cytosine-5 RNA methyltransferase NSun2 is a component of the chromatoid body and required for testis differentiation. Mol Cell Biol. 2013;33(8):1561–70. 10.1128/MCB.01523-12 23401851 PMC3624257

[88] Braun DA, Shril S, Sinha A, Schneider R, Tan W, Ashraf S, et al Mutations in *WDR4* as a new cause of Galloway-Mowat syndrome. Am J Med Genet A. 2018;176(11):2460–5. 10.1002/ajmg.a.40489 30079490 PMC6289609

[89] Lamichhane TN, Arimbasseri AG, Rijal K, Iben JR, Wei FY, Tomizawa K, et al Lack of tRNA-i6A modification causes mitochondrial-like metabolic deficiency in *S*. *pombe* by limiting activity of cytosolic tRNATyr, not mito-tRNA. RNA. 2016;22(4):583–96. 10.1261/rna.054064.115 26857223 PMC4793213

[90] Johansson MJ, Esberg A, Huang B, Bjork GR, Bystrom AS. Eukaryotic wobble uridine modifications promote a functionally redundant decoding system. Mol Cell Biol. 2008;28(10):3301–12. 10.1128/MCB.01542-07 18332122 PMC2423140

[91] Reuter JS, Mathews DH. RNAstructure: software for RNA secondary structure prediction and analysis. BMC Bioinformatics. 2010;11:129 10.1186/1471-2105-11-129 20230624 PMC2984261

[92] Jinek M, Coyle SM, Doudna JA. Coupled 5' nucleotide recognition and processivity in Xrn1-mediated mRNA decay. Mol Cell. 2011;41(5):600–8. 10.1016/j.molcel.2011.02.004 21362555 PMC3090138

[93] Robertus JD, Ladner JE, Finch JT, Rhodes D, Brown RS, Clark BF, et al Structure of yeast phenylalanine tRNA at 3 A resolution. Nature. 1974;250(467):546–51. 10.1038/250546a0 4602655

[94] Kim SH, Suddath FL, Quigley GJ, McPherson A, Sussman JL, Wang AH, et al Three-dimensional tertiary structure of yeast phenylalanine transfer RNA. Science. 1974;185(4149):435–40. 10.1126/science.185.4149.435 4601792

[95] Westhof E, Dumas P, Moras D. Crystallographic refinement of yeast aspartic acid transfer RNA. J Mol Biol. 1985;184(1):119–45. 10.1016/0022-2836(85)90048-8 3897553

[96] Johnson AW. Rat1p and Xrn1p are functionally interchangeable exoribonucleases that are restricted to and required in the nucleus and cytoplasm, respectively. Mol Cell Biol. 1997;17(10):6122–30. 10.1128/mcb.17.10.6122 9315672 PMC232462

[97] Shaheen HH, Hopper AK. Retrograde movement of tRNAs from the cytoplasm to the nucleus in *Saccharomyces cerevisiae*. Proc Natl Acad Sci U S A. 2005;102(32):11290–5. 10.1073/pnas.0503836102 16040803 PMC1183567

[98] Shaheen HH, Horetsky RL, Kimball SR, Murthi A, Jefferson LS, Hopper AK. Retrograde nuclear accumulation of cytoplasmic tRNA in rat hepatoma cells in response to amino acid deprivation. Proc Natl Acad Sci U S A. 2007;104(21):8845–50. 10.1073/pnas.0700765104 17502605 PMC1868590

[99] Takano A, Endo T, Yoshihisa T. tRNA Actively Shuttles Between the Nucleus and Cytosol in Yeast. Science. 2005;309:140–2. 10.1126/science.1113346 15905365

[100] Kramer EB, Hopper AK. Retrograde transfer RNA nuclear import provides a new level of tRNA quality control in *Saccharomyces cerevisiae*. Proc Natl Acad Sci U S A. 2013;110(52):21042–7. 10.1073/pnas.1316579110 24297920 PMC3876269

[101] Chalamcharla VR, Folco HD, Dhakshnamoorthy J, Grewal SI. Conserved factor Dhp1/Rat1/Xrn2 triggers premature transcription termination and nucleates heterochromatin to promote gene silencing. Proc Natl Acad Sci U S A. 2015;112(51):15548–55. 10.1073/pnas.1522127112 26631744 PMC4697380

[102] Wan Y, Hopper AK. From powerhouse to processing plant: conserved roles of mitochondrial outer membrane proteins in tRNA splicing. Genes Dev. 2018;32(19–20):1309–14. 10.1101/gad.316257.118 30228203 PMC6169838

[103] Payea MJ, Hauke AC, De Zoysa T, Phizicky EM. Mutations in the anticodon stem of tRNA cause accumulation and Met22-dependent decay of pre-tRNA in yeast. RNA. 2020;26(1):29–43. 10.1261/rna.073155.119 31619505 PMC6913130

[104] Zinshteyn B, Gilbert WV. Loss of a conserved tRNA anticodon modification perturbs cellular signaling. PLoS Genetics. 2013;9(8):e1003675 10.1371/journal.pgen.1003675 23935536 PMC3731203

[105] Chou HJ, Donnard E, Gustafsson HT, Garber M, Rando OJ. Transcriptome-wide Analysis of Roles for tRNA Modifications in Translational Regulation. Mol Cell. 2017;68(5):978–92 e4. 10.1016/j.molcel.2017.11.002 PMC5728682 29198561

[106] Pintard L, Lecointe F, Bujnicki JM, Bonnerot C, Grosjean H, Lapeyre B. Trm7p catalyses the formation of two 2'-*O*-methylriboses in yeast tRNA anticodon loop. EMBO J. 2002;21(7):1811–20. 10.1093/emboj/21.7.1811 11927565 PMC125368

[107] Han L, Kon Y, Phizicky EM. Functional importance of Psi38 and Psi39 in distinct tRNAs, amplified for tRNAGln(UUG) by unexpected temperature sensitivity of the s2U modification in yeast. RNA. 2015;21(2):188–201. 10.1261/rna.048173.114 25505024 PMC4338347

[108] Xu F, Bystrom AS, Johansson MJO. SSD1 suppresses phenotypes induced by the lack of Elongator-dependent tRNA modifications. PLoS Genetics. 2019;15(8):e1008117 10.1371/journal.pgen.1008117 31465447 PMC6738719

[109] Wek RC. Role of eIF2alpha Kinases in Translational Control and Adaptation to Cellular Stress. Cold Spring Harb Perspect Biol. 2018;10(7). 10.1101/cshperspect.a032870 PMC6028073 29440070

[110] Rashidi A, Miska J, Lee-Chang C, Kanojia D, Panek WK, Lopez-Rosas A, et al GCN2 is essential for CD8(+) T cell survival and function in murine models of malignant glioma. Cancer Immunol Immunother. 2020;69(1):81–94. 10.1007/s00262-019-02441-6 31844909 PMC6952559

[111] Manaud G, Nossent EJ, Lambert M, Ghigna MR, Boet A, Vinhas MC, et al Comparison of Human and Experimental Pulmonary Veno-Occlusive Disease. Am J Respir Cell Mol Biol. 2020. 10.1165/rcmb.2019-0015OC 32209028

[112] Turowski TW, Karkusiewicz I, Kowal J, Boguta M. Maf1-mediated repression of RNA polymerase III transcription inhibits tRNA degradation via RTD pathway. RNA. 2012;18(10):1823–32. 10.1261/rna.033597.112 22919049 PMC3446706

[113] Yang R, Wek SA, Wek RC. Glucose limitation induces GCN4 translation by activation of Gcn2 protein kinase. Mol Cell Biol. 2000;20(8):2706–17. 10.1128/mcb.20.8.2706-2717.2000 10733573 PMC85486

[114] Narasimhan J, Staschke KA, Wek RC. Dimerization is required for activation of eIF2 kinase Gcn2 in response to diverse environmental stress conditions. J Biol Chem. 2004;279(22):22820–32. 10.1074/jbc.M402228200 15010461

[115] Zhan K, Narasimhan J, Wek RC. Differential activation of eIF2 kinases in response to cellular stresses in *Schizosaccharomyces pombe*. Genetics. 2004;168(4):1867–75. 10.1534/genetics.104.031443 15611163 PMC1448706

[116] Chen D, Toone WM, Mata J, Lyne R, Burns G, Kivinen K, et al Global transcriptional responses of fission yeast to environmental stress. Mol Biol Cell. 2003;14(1):214–29. 10.1091/mbc.e02-08-0499 12529438 PMC140239

[117] Cartlidge RA, Knebel A, Peggie M, Alexandrov A, Phizicky EM, Cohen P. The tRNA methylase METTL1 is phosphorylated and inactivated by PKB and RSK in vitro and in cells. EMBO J. 2005;24(9):1696–705. 10.1038/sj.emboj.7600648 15861136 PMC1142581

[118] Huber SM, Leonardi A, Dedon PC, Begley TJ. The Versatile Roles of the tRNA Epitranscriptome during Cellular Responses to Toxic Exposures and Environmental Stress. Toxics. 2019;7(1). 10.3390/toxics7010017 PMC6468425 30934574

[119] Gu C, Begley TJ, Dedon PC. tRNA modifications regulate translation during cellular stress. FEBS Lett. 2014;588(23):4287–96. 10.1016/j.febslet.2014.09.038 25304425 PMC4403629

[120] Torres AG, Reina O, Stephan-Otto Attolini C, Ribas de Pouplana L. Differential expression of human tRNA genes drives the abundance of tRNA-derived fragments. Proc Natl Acad Sci U S A. 2019;116(17):8451–6. 10.1073/pnas.1821120116 30962382 PMC6486751

[121] Dittmar KA, Goodenbour JM, Pan T. Tissue-specific differences in human transfer RNA expression. PLoS Genetics. 2006;2(12):e221 10.1371/journal.pgen.0020221 17194224 PMC1713254

[122] Pavon-Eternod M, Gomes S, Geslain R, Dai Q, Rosner MR, Pan T. tRNA over-expression in breast cancer and functional consequences. Nucleic Acids Res. 2009;37(21):7268–80. 10.1093/nar/gkp787 19783824 PMC2790902

[123] Gingold H, Tehler D, Christoffersen NR, Nielsen MM, Asmar F, Kooistra SM, et al A dual program for translation regulation in cellular proliferation and differentiation. Cell. 2014;158(6):1281–92. 10.1016/j.cell.2014.08.011 25215487

[124] Pavon-Eternod M, Gomes S, Rosner MR, Pan T. Overexpression of initiator methionine tRNA leads to global reprogramming of tRNA expression and increased proliferation in human epithelial cells. RNA. 2013;19(4):461–6. 10.1261/rna.037507.112 23431330 PMC3677255

[125] Ishimura R, Nagy G, Dotu I, Zhou H, Yang XL, Schimmel P, et al RNA function. Ribosome stalling induced by mutation of a CNS-specific tRNA causes neurodegeneration. Science. 2014;345(6195):455–9. 10.1126/science.1249749 25061210 PMC4281038

[126] Kim DU, Hayles J, Kim D, Wood V, Park HO, Won M, et al Analysis of a genome-wide set of gene deletions in the fission yeast *Schizosaccharomyces pombe*. Nature Biotechnol. 2010;28(6):617–23. 20473289 10.1038/nbt.1628 PMC3962850

[127] Bahler J, Wu JQ, Longtine MS, Shah NG, McKenzie A 3rd, Steever AB, et al Heterologous modules for efficient and versatile PCR-based gene targeting in *Schizosaccharomyces pombe*. Yeast. 1998;14(10):943–51. 10.1002/(SICI)1097-0061(199807)14:10<943::AID-YEA292>3.0.CO;2-Y 9717240

[128] Giaever G, Chu AM, Ni L, Connelly C, Riles L, Veronneau S, et al Functional profiling of the *Saccharomyces cerevisiae* genome. Nature. 2002;418(6896):387–91. 10.1038/nature00935 12140549

[129] Sherman F. Getting started with yeast. Methods Enzymol. 1991;194:3–21. 10.1016/0076-6879(91)94004-v 2005794

[130] Elder RT, Loh EY, Davis RW. RNA from the yeast transposable element Ty1 has both ends in the direct repeats, a structure similar to retrovirus RNA. Proc Natl Acad Sci U S A. 1983;80(9):2432–6. 10.1073/pnas.80.9.2432 6189122 PMC393839

[131] Preston MA, D'Silva S, Kon Y, Phizicky EM. tRNAHis 5-methylcytidine levels increase in response to several growth arrest conditions in *Saccharomyces cerevisiae*. RNA. 2013;19(2):243–56. 10.1261/rna.035808.112 23249748 PMC3543094

[132] Jackman JE, Montange RK, Malik HS, Phizicky EM. Identification of the yeast gene encoding the tRNA m1G methyltransferase responsible for modification at position 9. RNA. 2003;9(5):574–85. 10.1261/rna.5070303 12702816 PMC1370423

[133] Guy MP, Podyma BM, Preston MA, Shaheen HH, Krivos KL, Limbach PA, et al Yeast Trm7 interacts with distinct proteins for critical modifications of the tRNAPhe anticodon loop. RNA. 2012;18(10):1921–33. 10.1261/rna.035287.112 22912484 PMC3446714

[134] Lee SJ, Ramesh R, de Boor V, Gebler JM, Silva RC, Sattlegger E. Cost-effective and rapid lysis of *Saccharomyces cerevisiae* cells for quantitative western blot analysis of proteins, including phosphorylated eIF2alpha. Yeast. 2017;34(9):371–82. 10.1002/yea.3239 28568773

[135] Corpet F. Multiple sequence alignment with hierarchical clustering. Nucl Acids Res. 1988;16:10881–90. 10.1093/nar/16.22.10881 2849754 PMC338945

