# Supplementary figures and images for: Hypomodified tRNA in evolutionarily distant yeasts can trigger rapid tRNA decay to activate the general amino acid control response, but with different consequences
Source: PLoS Genet. 2020 Aug 25;16(8):e1008893. doi: 10.1371/journal.pgen.1008893 (PMC7473580; doi:10.1371/journal.pgen.1008893)

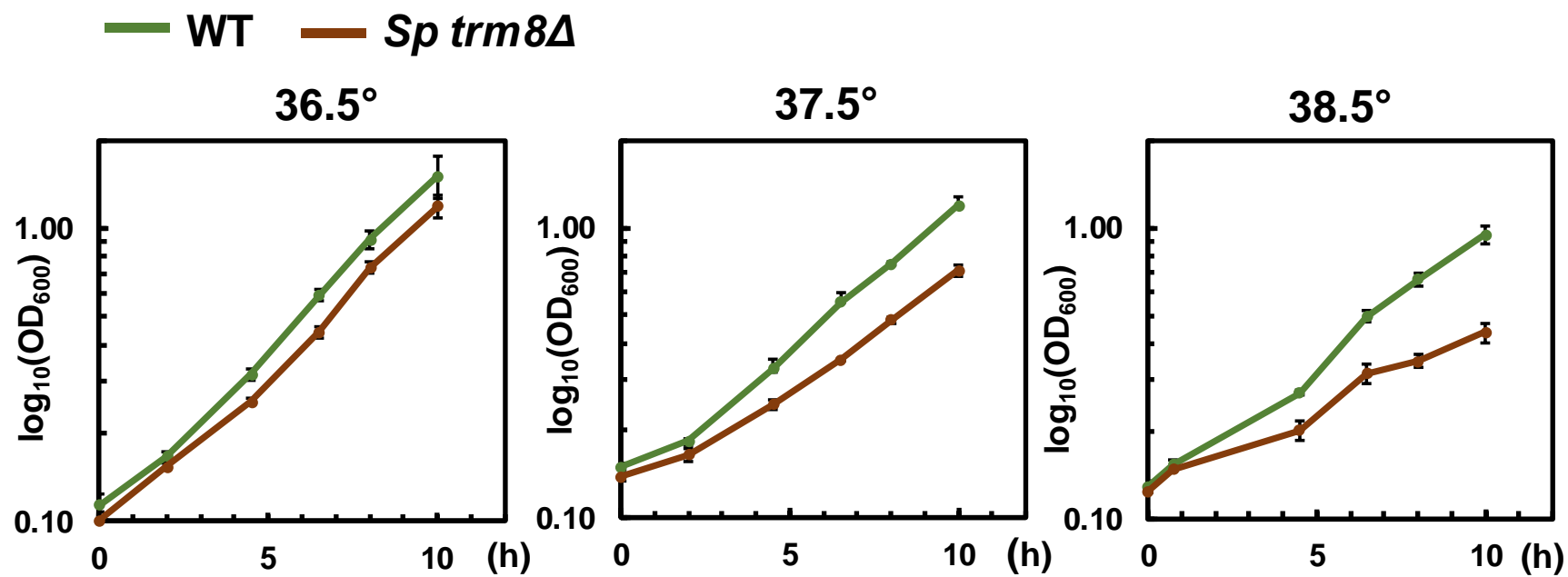

Supplement: S1 Fig — Strains were grown in YES media at 30°C, shifted to the indicated temperatures, and then growth was monitored for 8 hours before harvest as described in Materials and Methods, and tRNA analysis as done in Fig 2A and 2B. WT, green; Sp trm8Δ, brown. (PDF) [file pgen.1008893.s001.pdf]

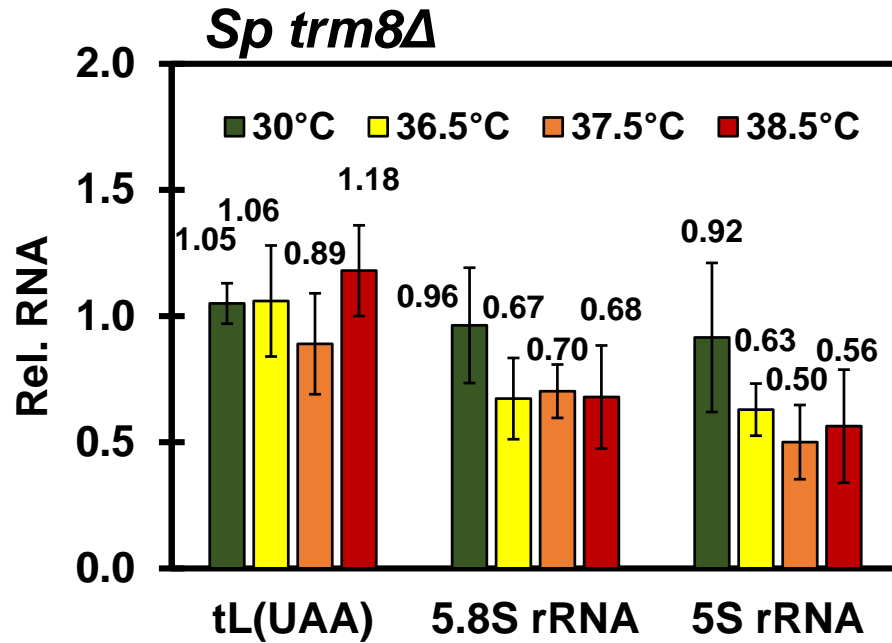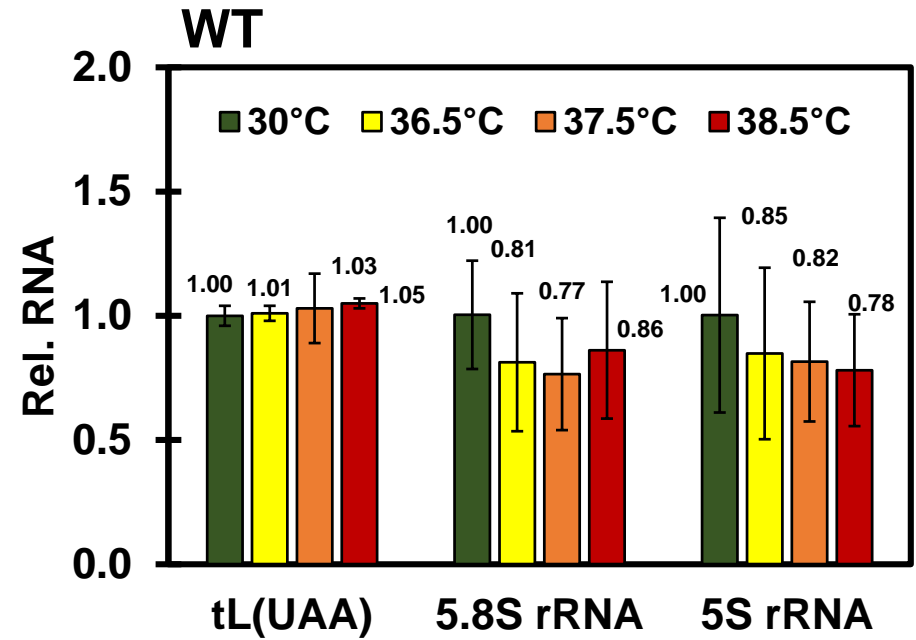

Supplement: S2 Fig — The northern blot shown in Fig 2A was used to analyze the non-Trm8 substrate tL(UAA), 5S rRNA, and 5.8S rRNA. The bar chart depicts levels of RNA species at each temperature, relative to their levels in the WT strain at 30°C (each value itself first normalized to levels of the control non-Trm8 substrate tG(GCC)). 30°C, green; 36.5°C, yellow; 37.5°C, orange; 38.5°C. red. (PDF) [file pgen.1008893.s002.pdf]

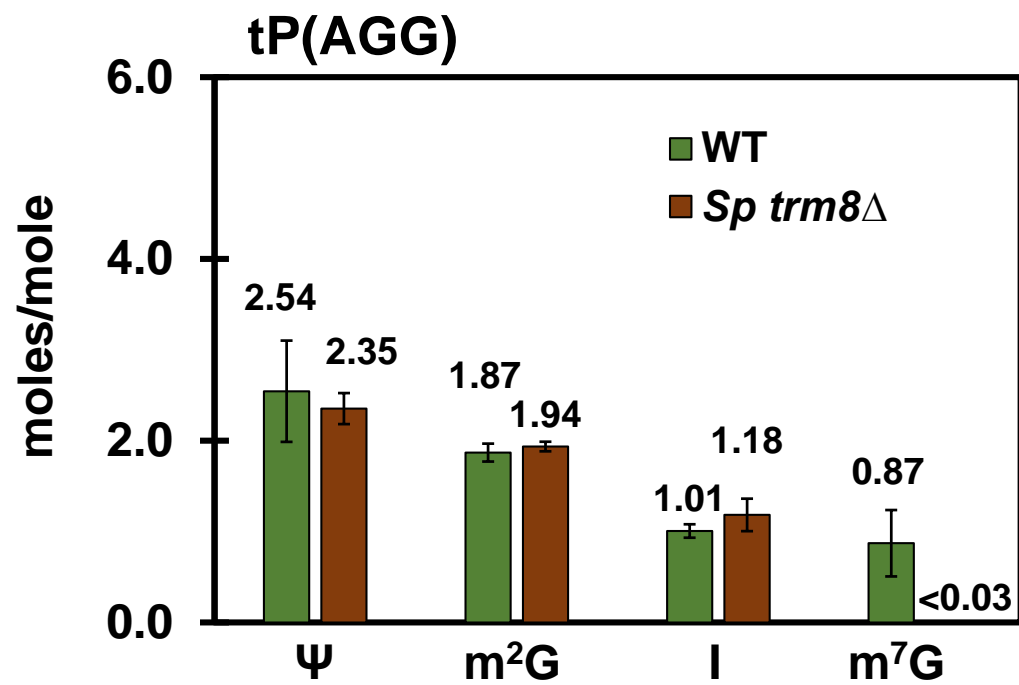

Supplement: S3 Fig — trm8Δ mutants and WT cells were grown in YES media at 30°C and tP(AGG) was purified and analyzed for modifications as in Fig 1A. (PDF) [file pgen.1008893.s003.pdf]

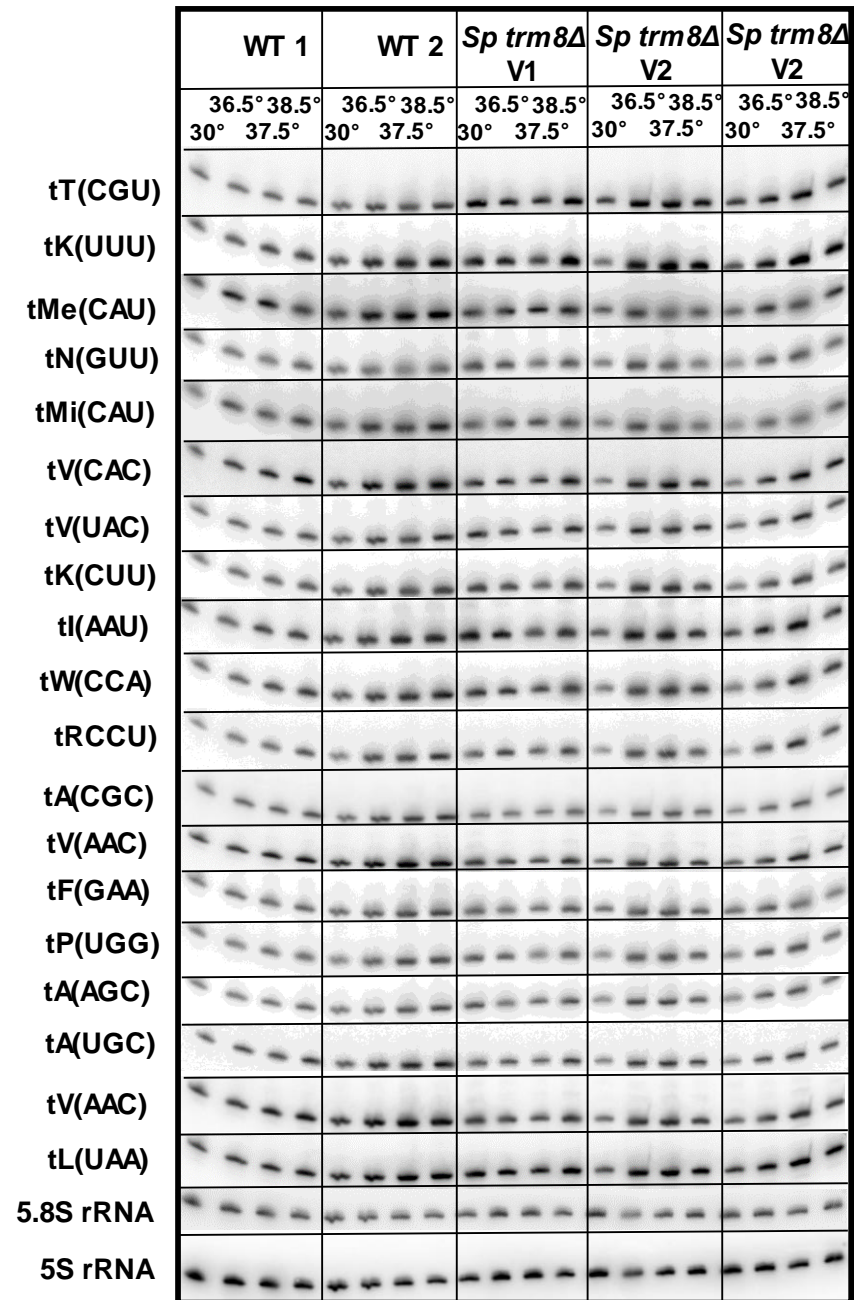

Supplement: S4 Fig — The northern blot shown in Fig 2A was continued to analyze levels of all other predicted Trm8 substrate tRNAs, as well as the non-Trm8 substrate tL(UAA), 5S RNA, and 5.8S RNA, in WT and trm8Δ mutants at different temperatures. (PDF) [file pgen.1008893.s004.pdf]

A.

*Sp trm8Δ*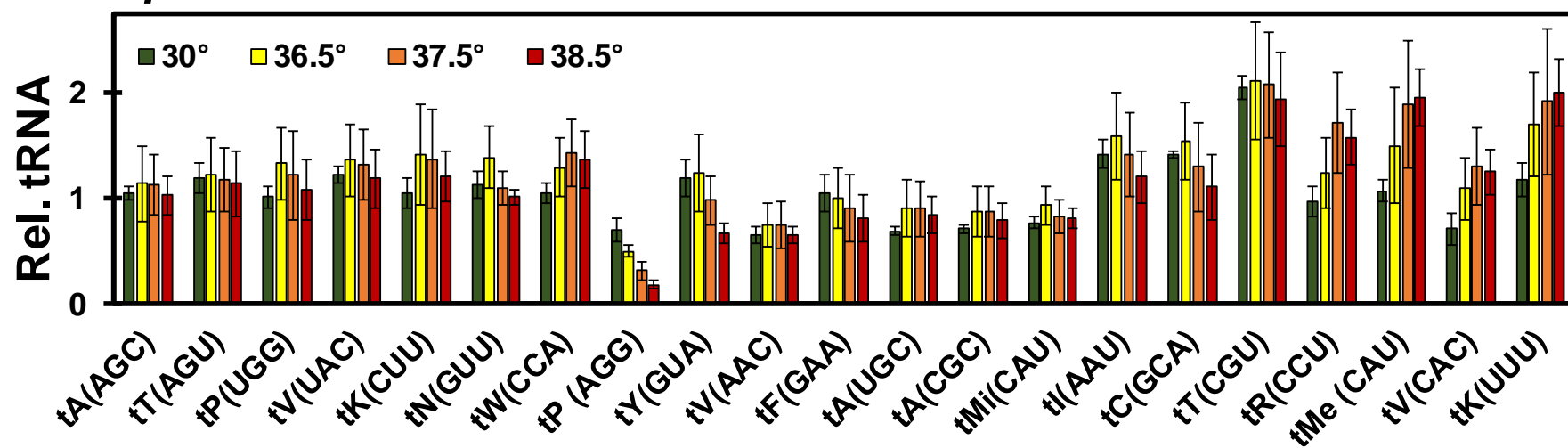

B.

WT

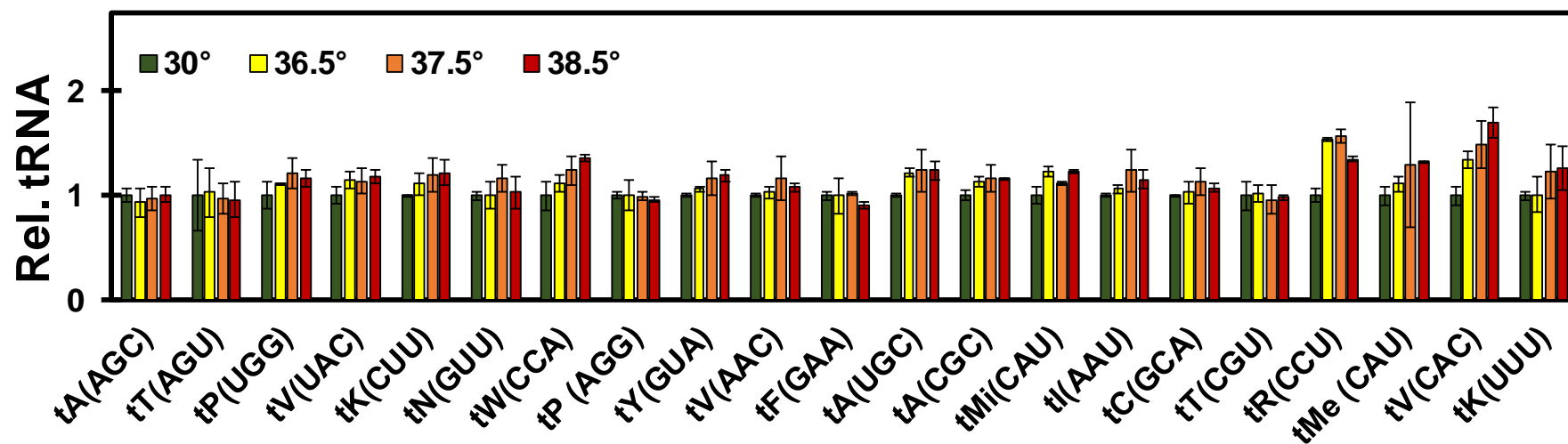

Supplement: S5 Fig — A. Among 21 predicted Trm8 substrate tRNAs, only tP(AGG) and tY(GUA) had reduced levels in S. pombe trm8Δ mutants at elevated temperatures. tRNA levels were quantified, relative to tG(GCC), as described in Fig 2B. Note that data from Fig 2B is also included here for completeness. B. Analysis of Trm8 substrate tRNAs in WT cells at elevated temperatures. (PDF) [file pgen.1008893.s005.pdf]

A.

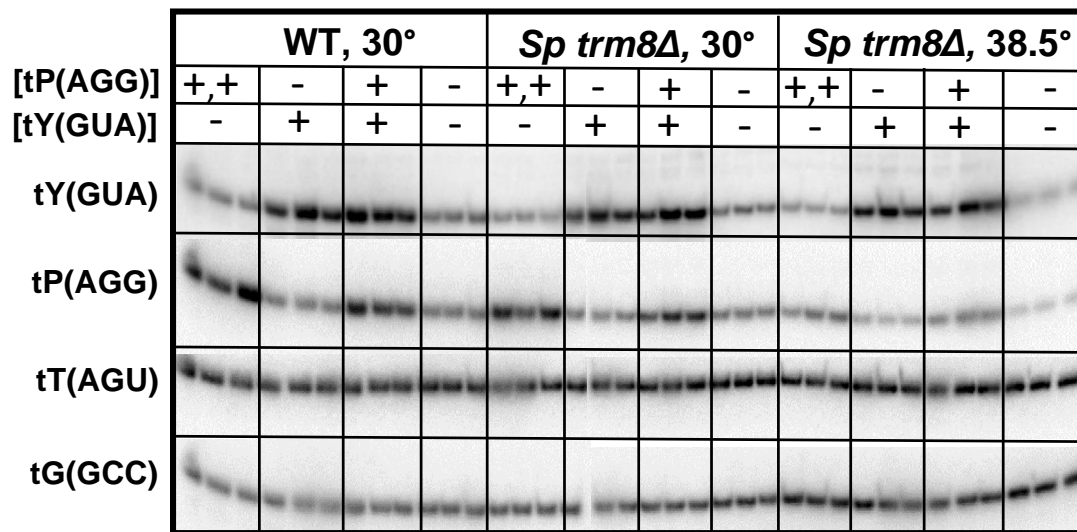

B.

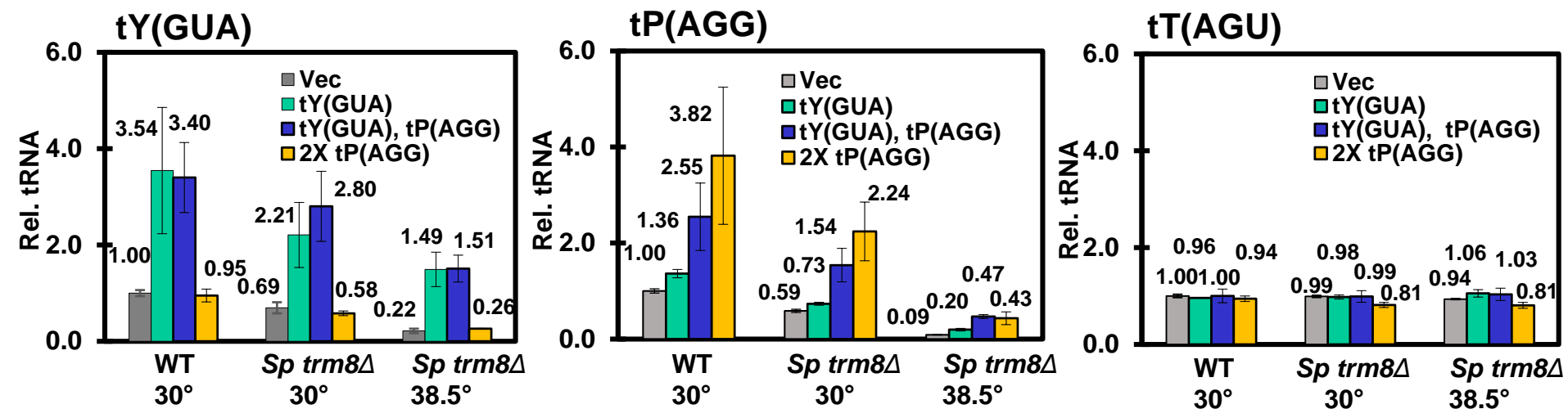

Supplement: S6 Fig — A. Overproduction of tY(GUA) and tP(AGG) resulted in increased levels of the corresponding tRNAs in S. pombe trm8Δ mutants and WT cells. Strains with plasmids as indicated were grown in EMMC-Leu media at 30°C and shifted to 38.5°C for 8 hours, and then RNA was isolated and analyzed by northern blotting as in Fig 2A. B. Quantification of tRNA levels in S. pombe trm8Δ mutants and WT cells overproducing tY(GUA) or tP(AGG). Quantification was done as in Fig 2B. (PDF) [file pgen.1008893.s006.pdf]

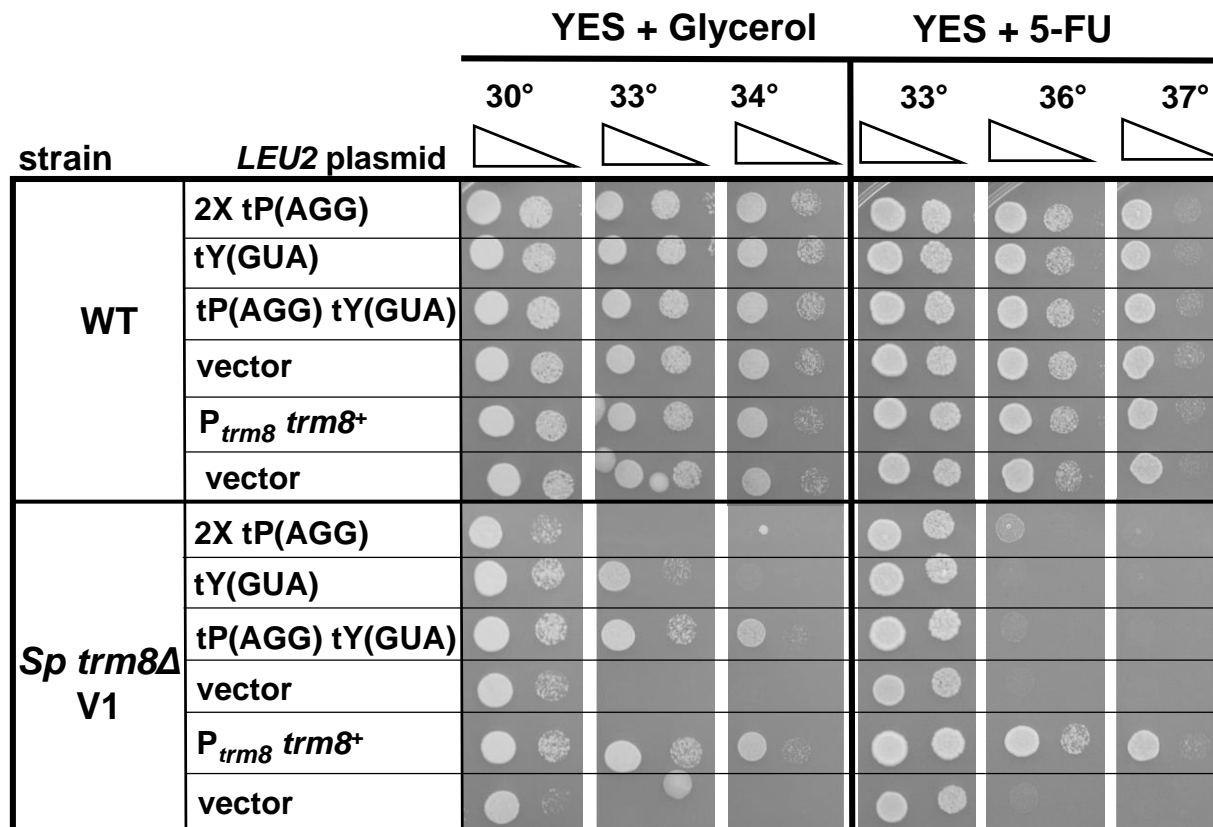

Supplement: S7 Fig — Strains grown for Fig 2D were analyzed for growth on plates containing YES media with 3% glycerol (instead of 3% glucose) and YES media with 5-FU (30 μg/ml). (PDF) [file pgen.1008893.s007.pdf]

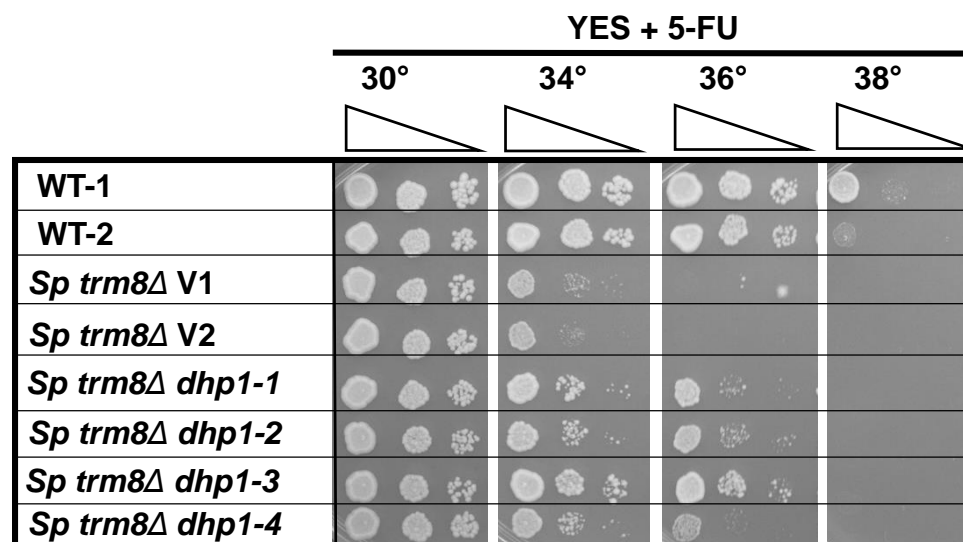

Supplement: S8 Fig — Strains grown for Fig 3A were analyzed for growth on YES + 5-FU (30 μg/ml) plates. (PDF) [file pgen.1008893.s008.pdf]

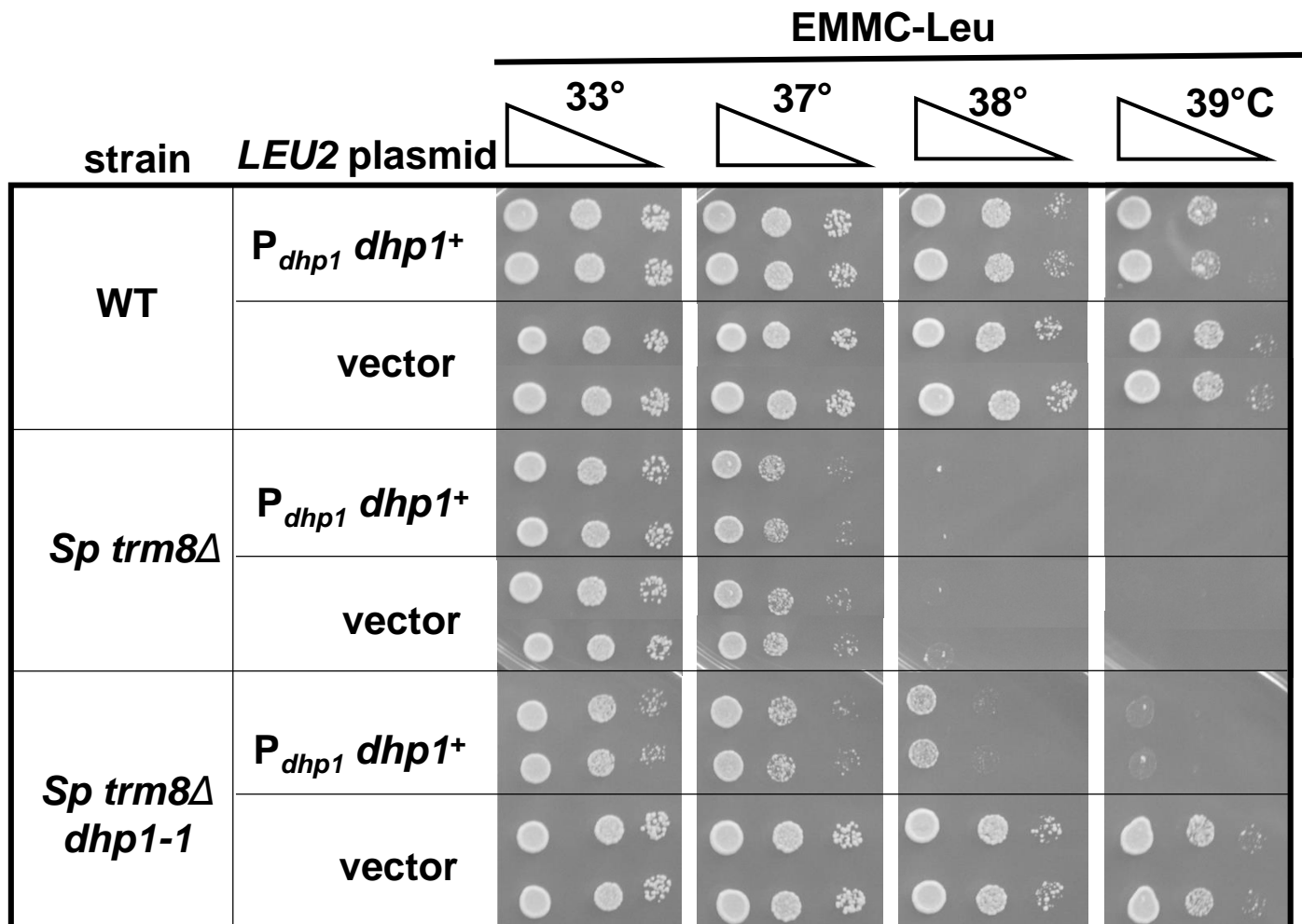

Supplement: S9 Fig — WT, trm8Δ, and trm8Δ dhp1-1 cells expressing Pdhp1 dhp1+ or a vector were grown overnight in EMMC-Leu media at 30°C, and analyzed for growth. (PDF) [file pgen.1008893.s009.pdf]

A.

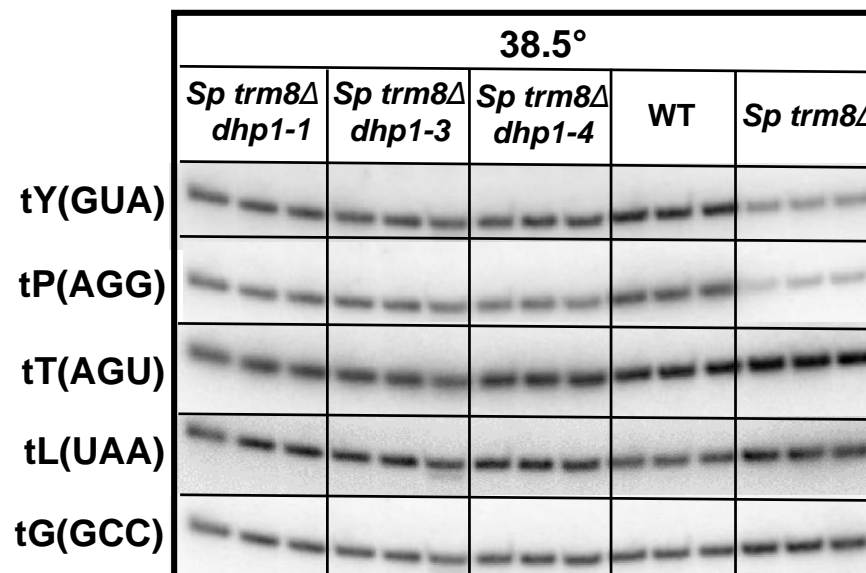

B.

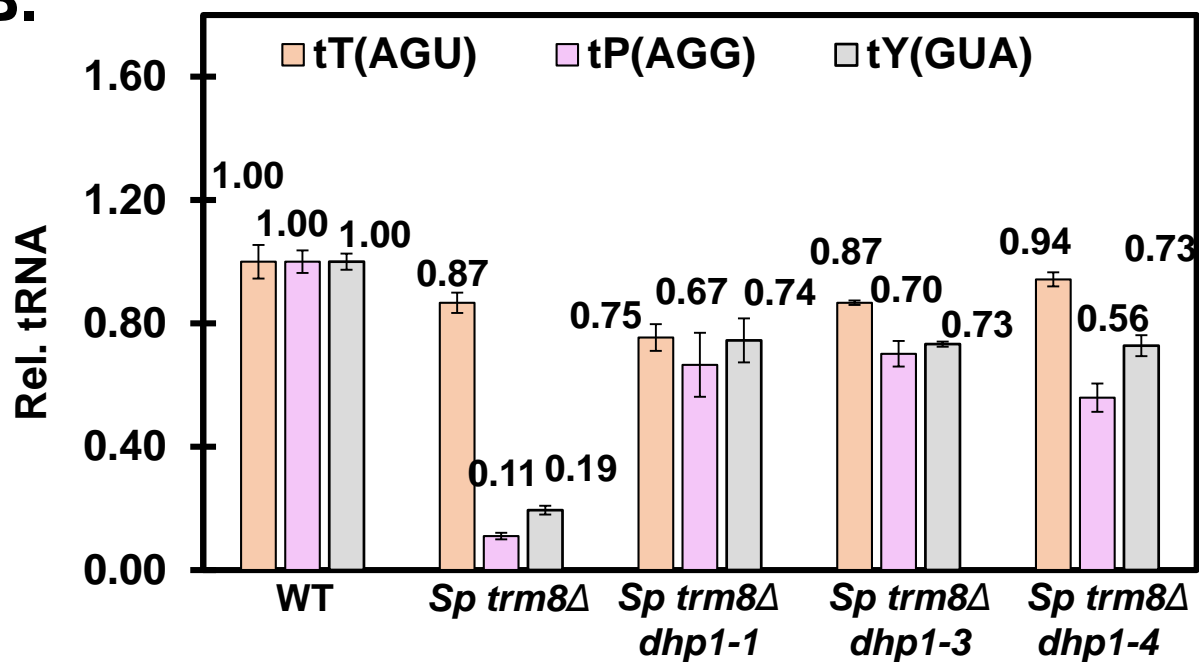

Supplement: S10 Fig — A. S. pombe trm8Δ dhp1-3 and trm8Δ dhp1-4 mutants also restored tY(GUA) and tP(AGG) tRNA levels at 38.5°C. Strains were grown in YES media at 30°C and shifted to 38.5°C for 8 hours, and RNA was isolated and analyzed by northern blotting as in Fig 2A. B. Quantification of tRNA levels in different S. pombe trm8Δ dhp1 mutants. tRNA levels were quantified as in Fig 2B. tT(AGU), brown; tP(AGG), purple; tY(GUA), gray. (PDF) [file pgen.1008893.s010.pdf]

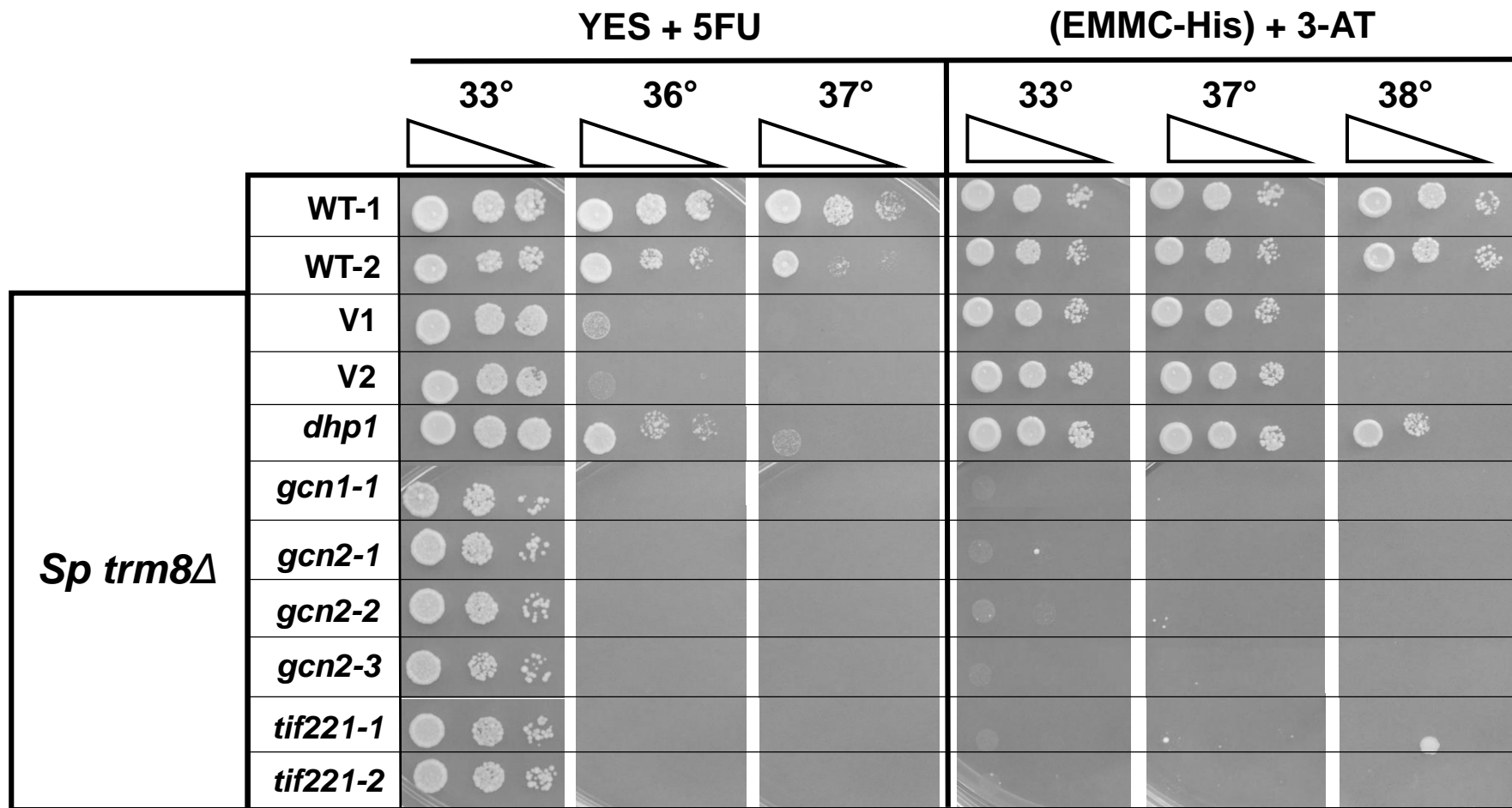

Supplement: S11 Fig — Strains grown for Fig 4A were analyzed for growth on plates containing YES media + 5-FU (30 μg/ml) and EMMC-His media + 10 mM 3-AT. (PDF) [file pgen.1008893.s011.pdf]

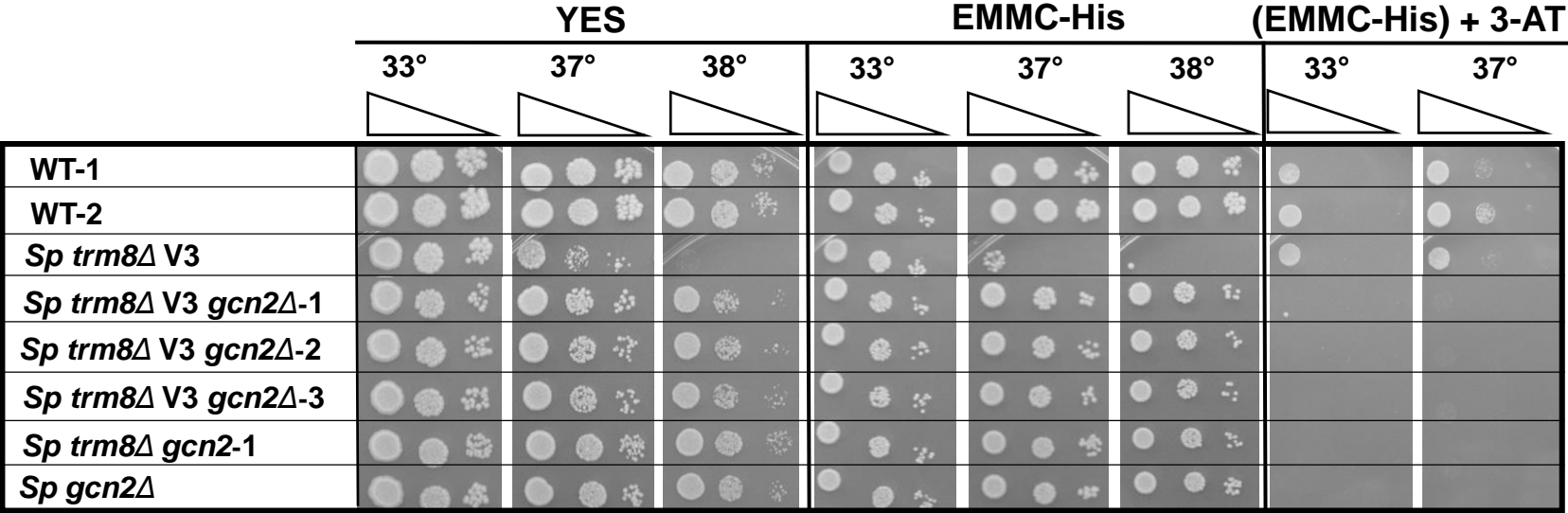

Supplement: S12 Fig — Strains were analyzed for growth on YES media, EMMC-His or EMMC-His media containing 10 mM 3-AT, as described in Fig 1B. Reconstructed S. pombe trm8Δ mutant was labeled as V3. (PDF) [file pgen.1008893.s012.pdf]

A.

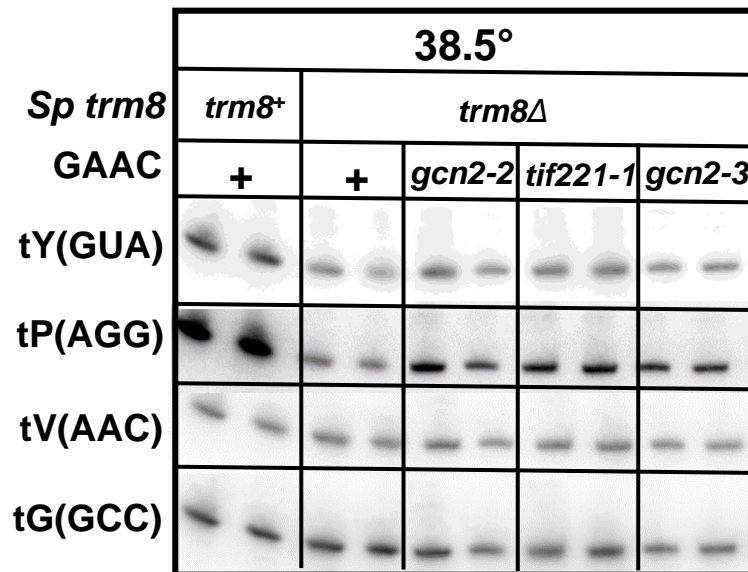

B.

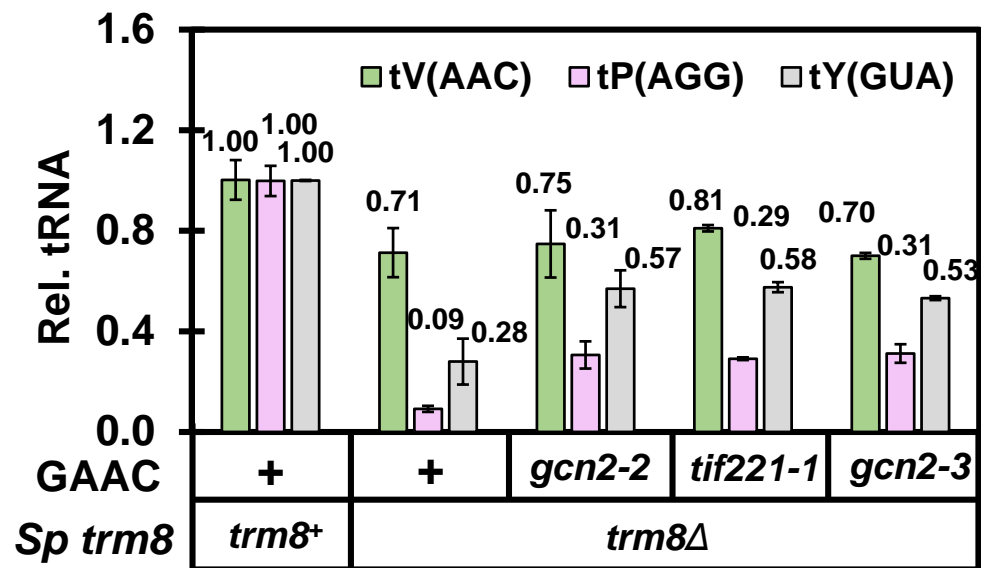

Supplement: S13 Fig — A. Northern analysis of WT, S. pombe trm8Δ, trm8Δ gcn2-2, trm8Δ tif221-1, and trm8Δ gcn2-3 cells. Strains were grown in YES media at 30°C and shifted to 38.5°C for 8 hours, and RNA was isolated and analyzed by northern blotting as in Fig 2A. B. Quantification of tRNA levels. tRNA levels were quantified as in Fig 2B. n = 2 for all strains. (PDF) [file pgen.1008893.s013.pdf]

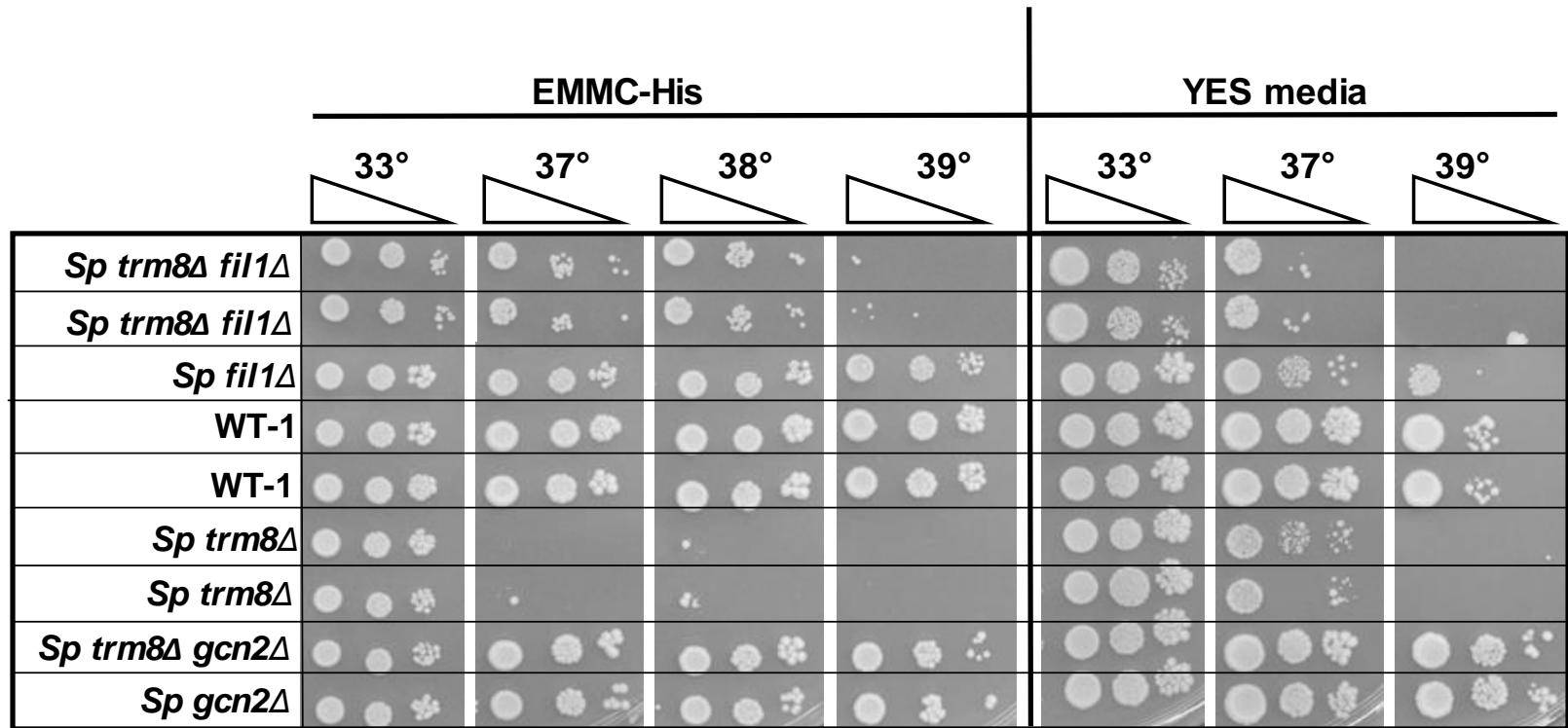

Supplement: S14 Fig — Strains were grown overnight in YES media at 30°C and analyzed for growth. (PDF) [file pgen.1008893.s014.pdf]

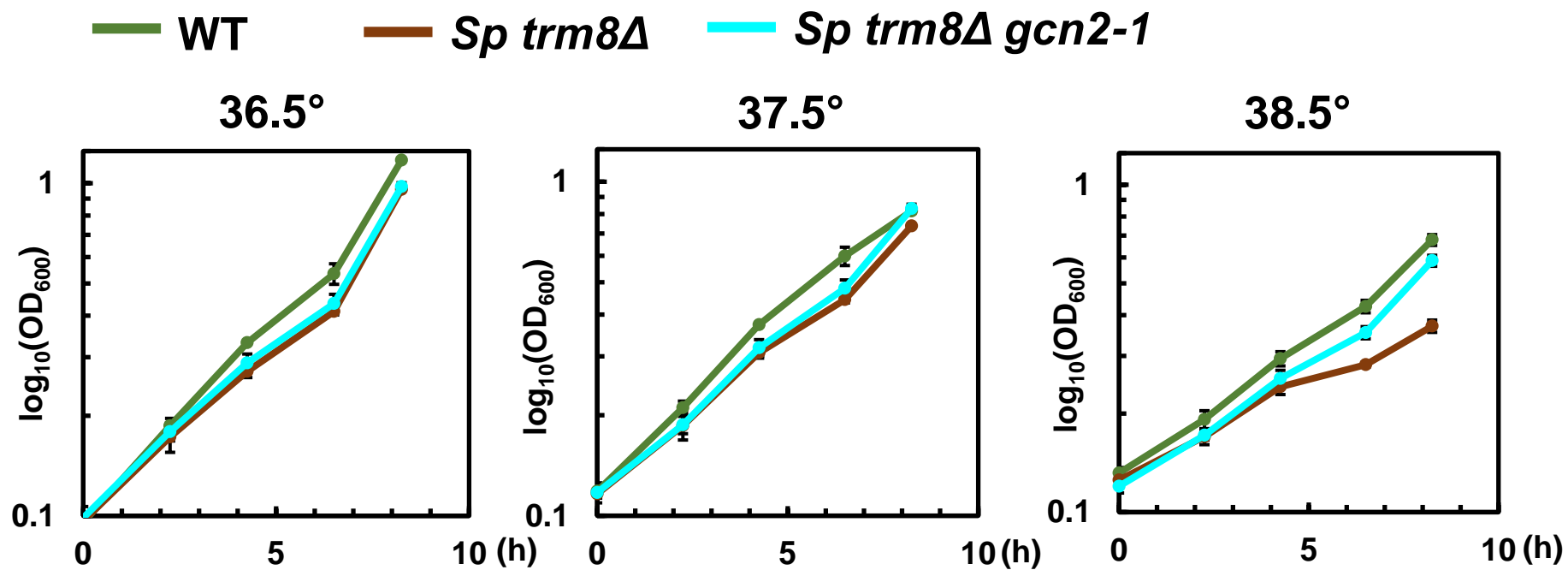

Supplement: S15 Fig — Strains were grown in YES media at 30°C, shifted to 36.5°C, 37.5°C, and 38.5°C as indicated, and then growth was monitored for 8 hours before harvest as described in Materials and Methods, and analysis of mRNAs and tRNAs in Figs 5A–5C and S16 and S19. (PDF) [file pgen.1008893.s015.pdf]

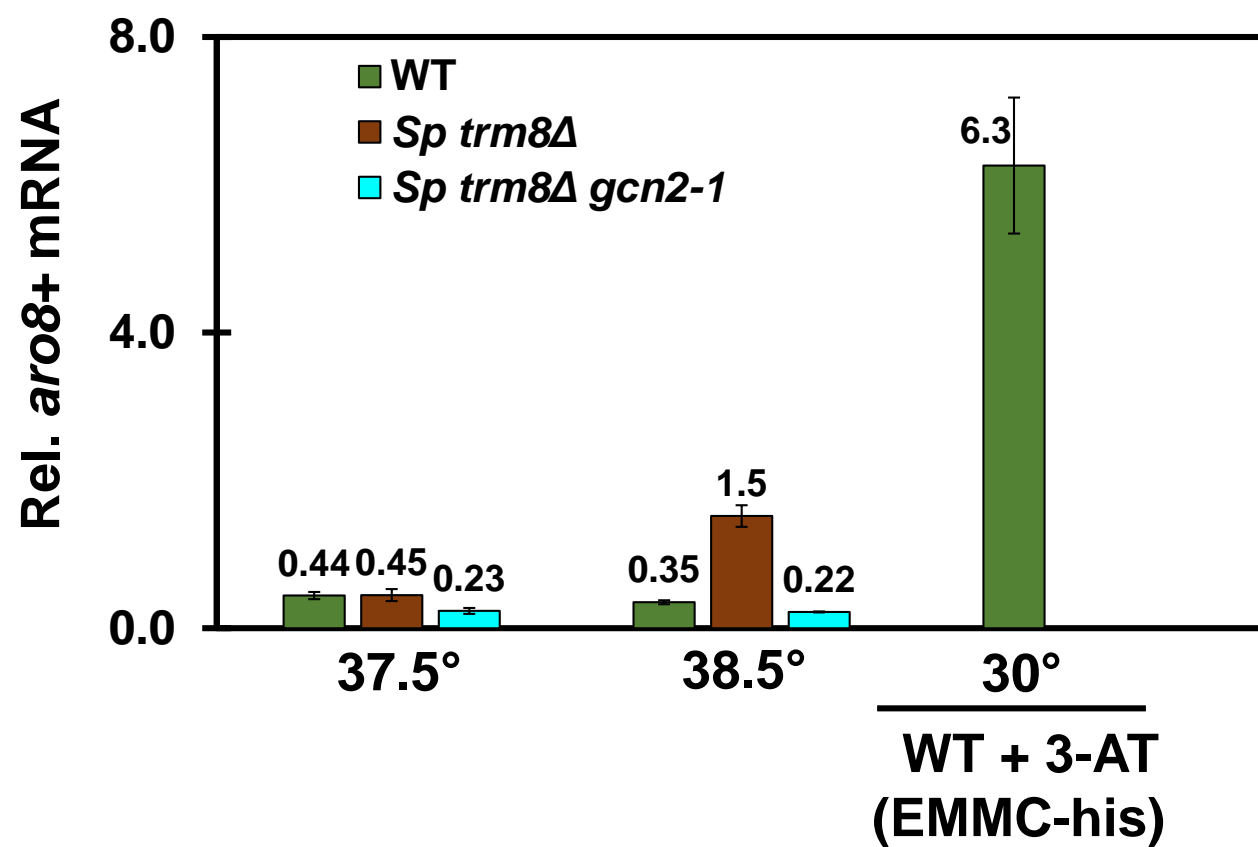

Supplement: S16 Fig — Bulk RNA from the growth in S15 Fig was used for the RT-qPCR analysis of aro8+(SPAC56E4.03) mRNA levels, as in Fig 5A. (PDF) [file pgen.1008893.s016.pdf]

A.

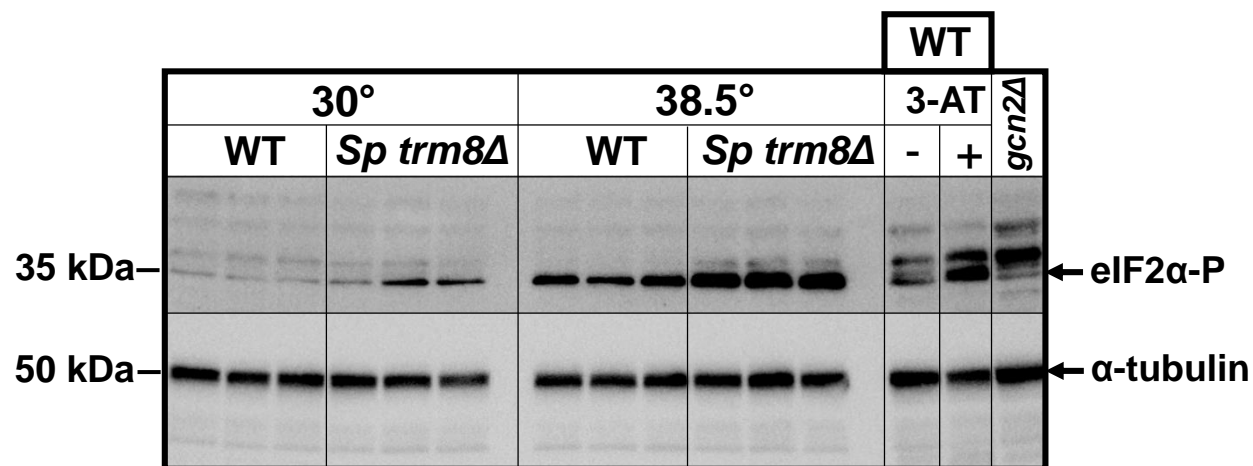

B.

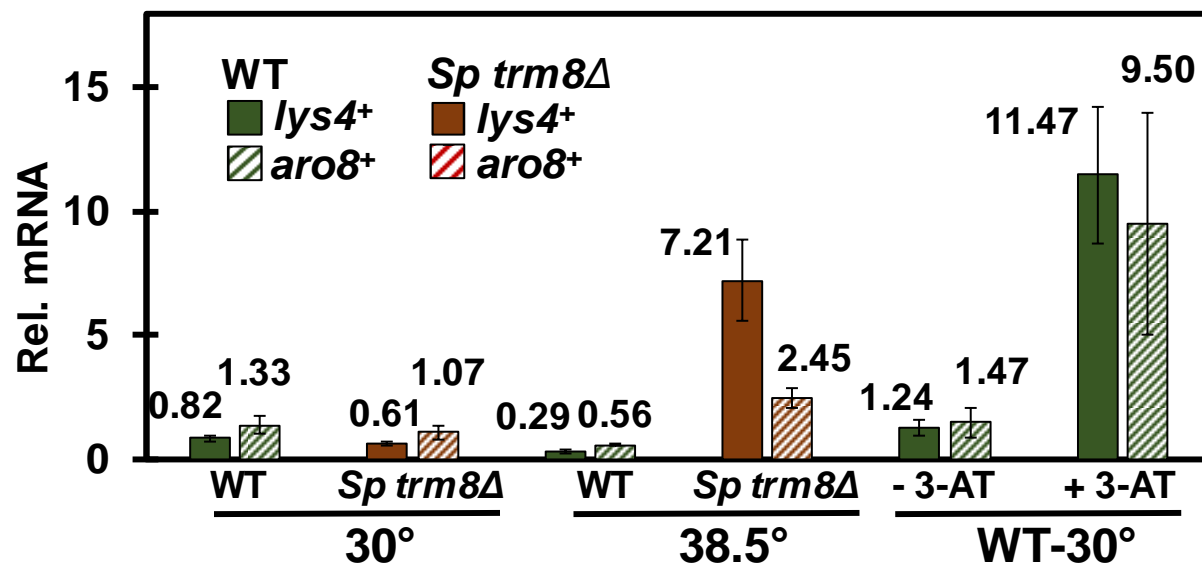

Supplement: S17 Fig — A. S. pombe trm8Δ mutants had increased levels of phophorylated eIF2α at 38.5°C WT and S. pombe trm8Δ strains were grown in YES media at 30°C, shifted to 38.5°C for 8 hours and cells were harvested. Then crude extracts were prepared and analyzed by western blotting as described in Materials and Methods, using anti-phosphorylated eIF2α and anti-α-tubulin. Controls: WT and gcn2Δ mutants were grown at 30°C in EMMC-His media and, where indicated, treated with 20 mM 3-AT. Then extracts were prepared and evaluated by blotting in parallel to the experimental samples. B. Increased levels of phophorylated eIF2α in S. pombe trm8Δ mutants at 38.5°C were associated with increased expression of lys4+ and aro8+ mRNAs. Bulk RNA was prepared from the growth done for S17A Fig, and levels of aro8+(SPAC56E4.03) and lys4+ mRNAs were quantified relative to act1+, using RT-qPCR, as in Fig 5A. (PDF) [file pgen.1008893.s017.pdf]

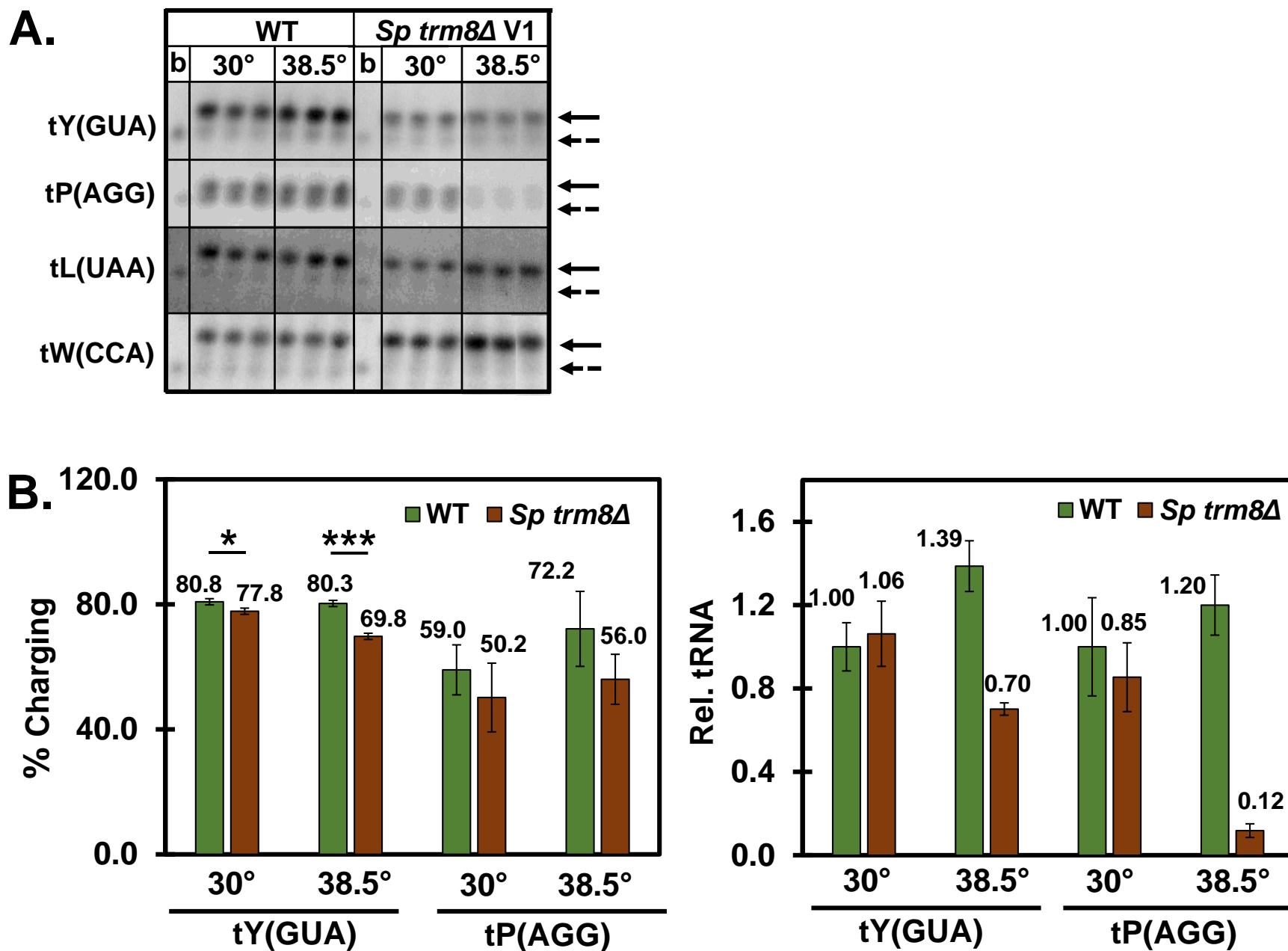

Supplement: S18 Fig — A. Analysis of charging levels of tY(GUA) or tP(AGG) in S. pombe trm8Δ mutants at 38.5°C. Strains were grown in YES media at 30°C and shifted to 38.5°C, and samples were harvested after 8 hours. Then bulk RNA was isolated and resolved by denaturing PAGE under acidic conditions (to preserve tRNA charging), transferred, and then analyzed by hybridization as described in Materials and Methods. Control samples (WT and S. pombe trm8Δ mutants) were treated with 1 mM EDTA and 0.1 M Tris-HCl (pH 9.0) for 30 min at 37°C to de-acylate the tRNA. b, base treated bulk RNA; Upper arrows, charged tRNA species; lower arrows with dashed lines, uncharged tRNA species. B. Quantification of tY(GUA) or tP(AGG) charging and tRNA levels. The percent charging was calculated as the ratio of aminoacylated species to the total for each tRNA. Relative levels of tP(AGG) and tY(GUA) were quantified as in Fig 2B, relative to the non-Trm8 substrate tL(UAA). (PDF) [file pgen.1008893.s018.pdf]

A.

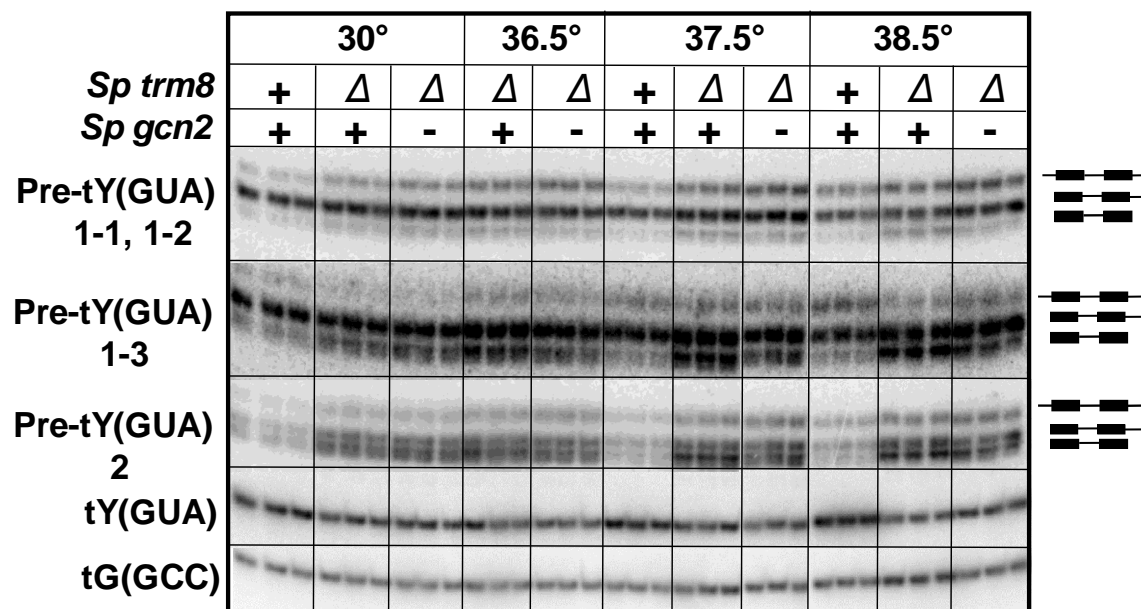

B.

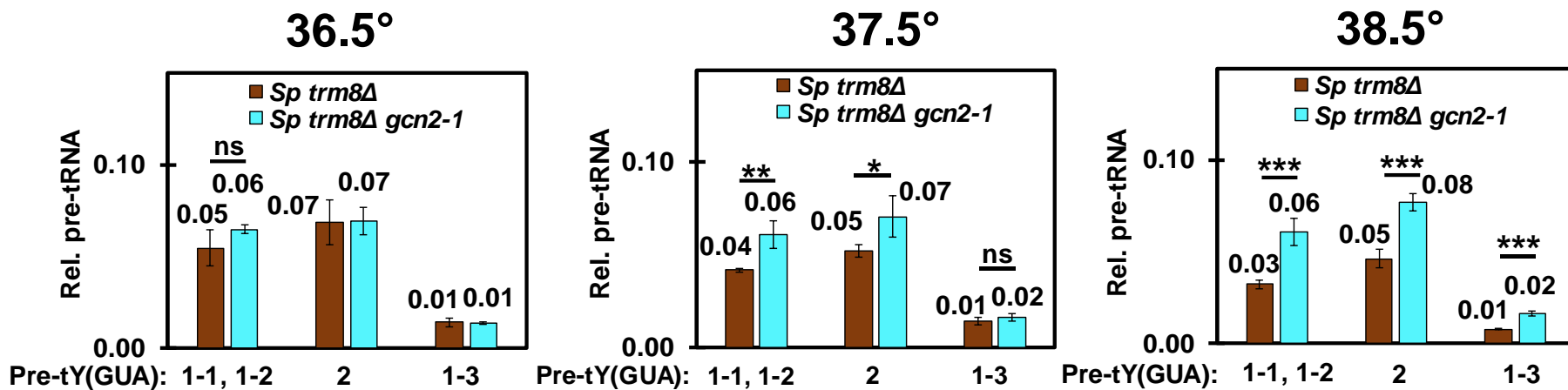

Supplement: S19 Fig — A. Northern analysis of pre-tY(GUA) levels in S. pombe WT, trm8Δ, and trm8Δ gcn2-1 mutants. The northern blot shown in Fig 5B was continued to analyze levels of pre-tY(GUA) species in WT and trm8Δ, and trm8Δ gcn2-1 mutants at different temperatures, using appropriate gene-specific probes (S5 Table) for the introns of the different tY(GUA) genes [59]. Cartoons at the right indicate exons, heavy bars; 5' leaders, 3' trailers, and introns, light bars. The primary pre-tY(GUA) transcript has 5' leader, 3' trailer, and intron, and the end-matured pre-tY(GUA) has only the intron. B. Quantification of pre-tY(GUA) transcript levels in S. pombe trm8Δ, and trm8Δ gcn2-1 mutants, from northern in S19A Fig. The primary pre-tY(GUA) transcript levels were normalized to levels of tG(GCC). (PDF) [file pgen.1008893.s019.pdf]

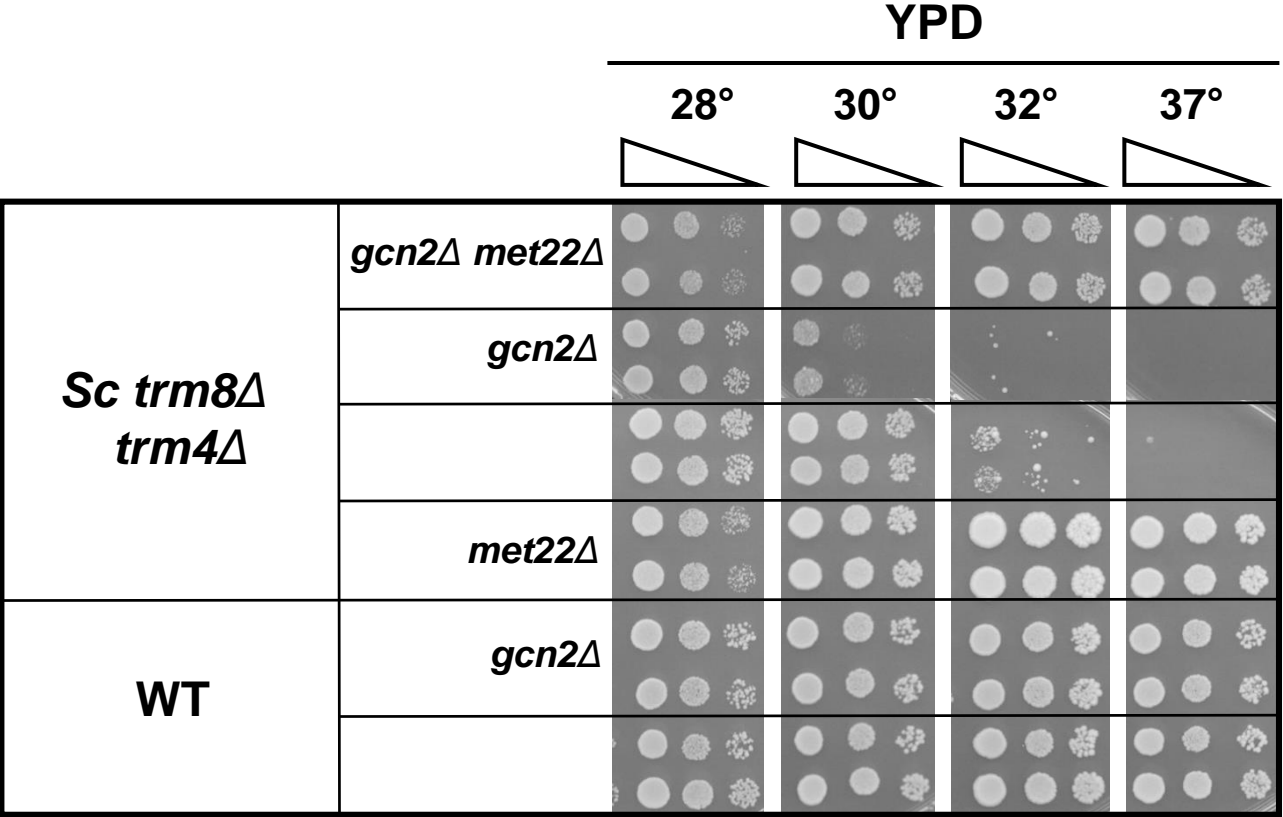

Supplement: S20 Fig — Strains were grown overnight in YPD media 28°C and analyzed for growth on YPD plates. (PDF) [file pgen.1008893.s020.pdf]

A.

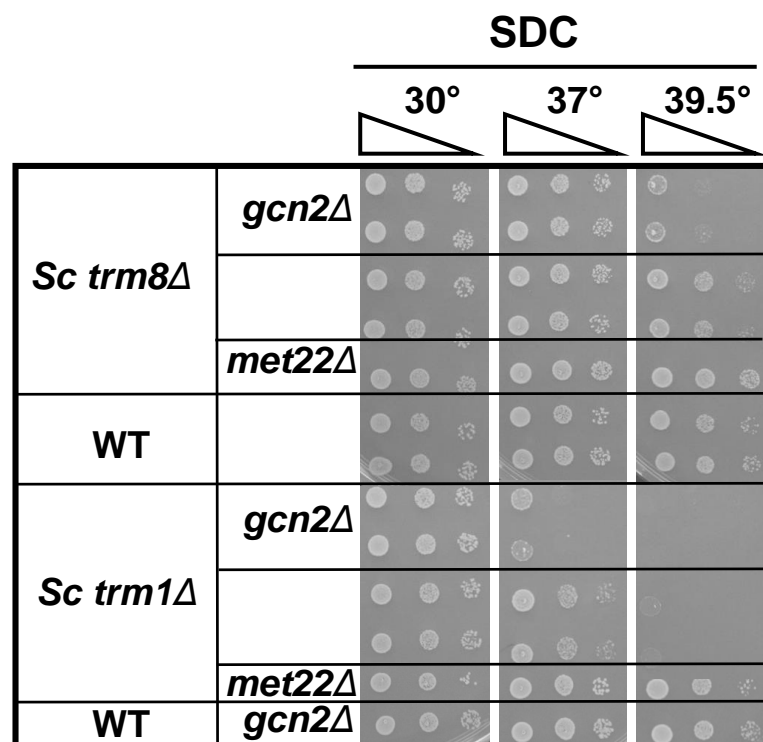

B.

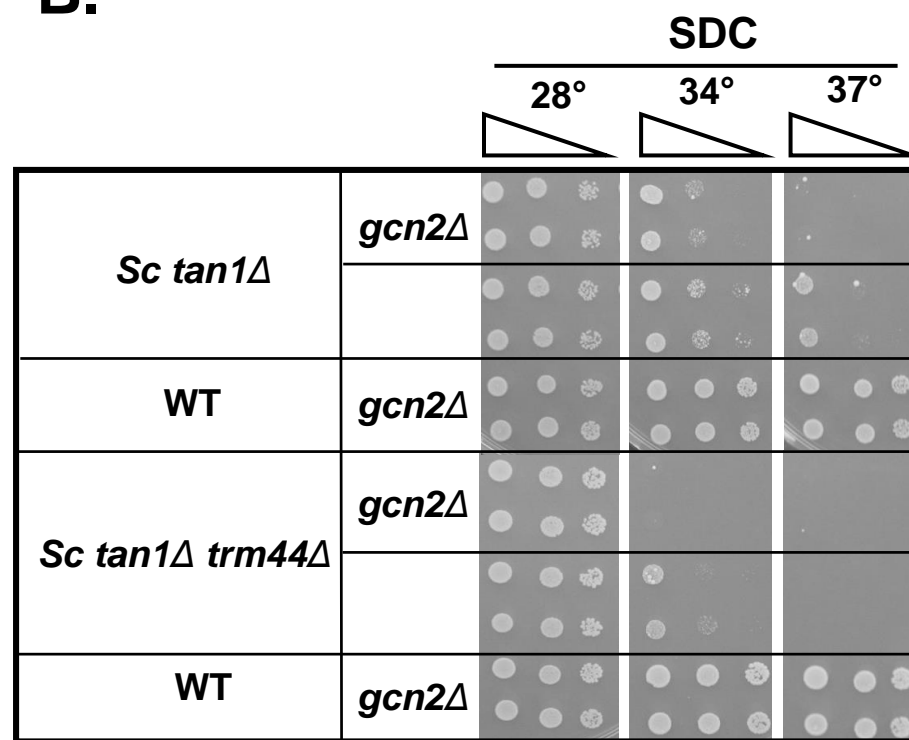

Supplement: S21 Fig — A. Deletion of GCN2 exacerbated the temperature sensitivity of S. cerevisiae trm8Δ and trm1Δ mutants in SDC media. Strains were grown overnight in YPD media at 28°C and analyzed for growth on SDC plates. B. Deletion of GCN2 exacerbated the temperature sensitivity of S. cerevisiae tan1Δ and tan1Δ trm44Δ mutants in SDC media. Strains were grown overnight in YPD media 28°C and analyzed for growth on SDC plates. (PDF) [file pgen.1008893.s021.pdf]

A.

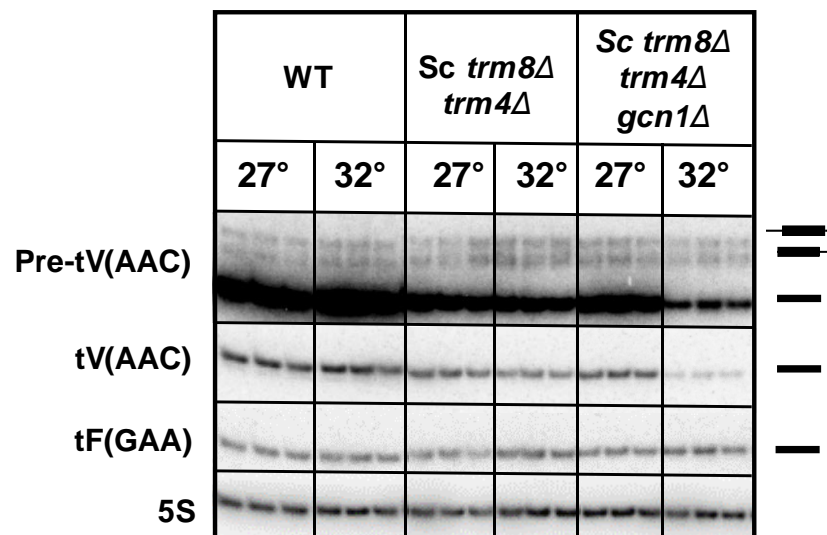

B.

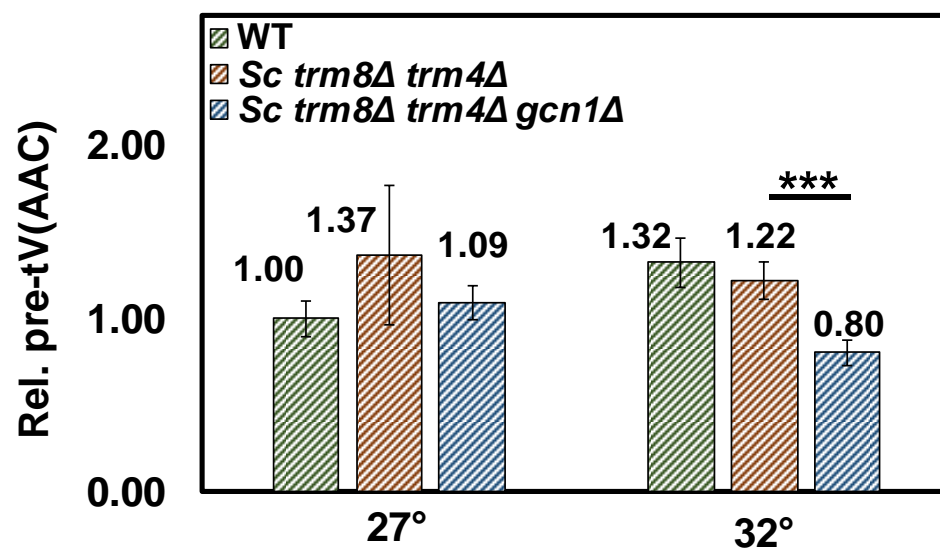

Supplement: S22 Fig — A. Northern analysis of pre-tV(AAC) levels in WT, S. cerevisiae trm8Δ trm4Δ, and trm8Δ trm4Δ gcn1Δ cells after shift from 28°C to 32°C. Bulk RNA from the growth done for Fig 6B and 6C was used for the northern analysis. B. Quantification of the levels of the primary pre-tV(AAC) transcript. pre-tV(AAC) levels were determined by hybridization with oligomer TDZ 415, specific for seven of the fourteen pre-tV(AAC) species, and then quantification of the upper band, corresponding to the primary transcript, with 5' leader and 3' trailer. Levels were normalized to 5S rRNA. (PDF) [file pgen.1008893.s022.pdf]

A.

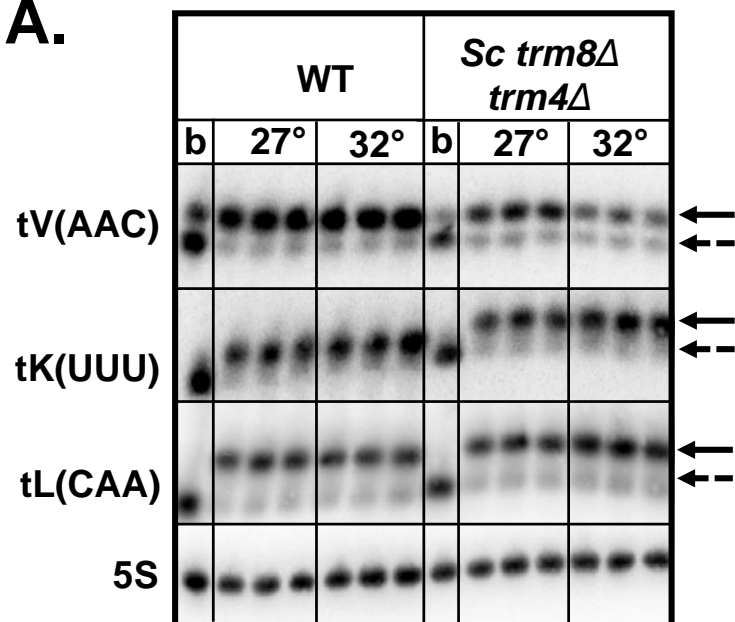

B.

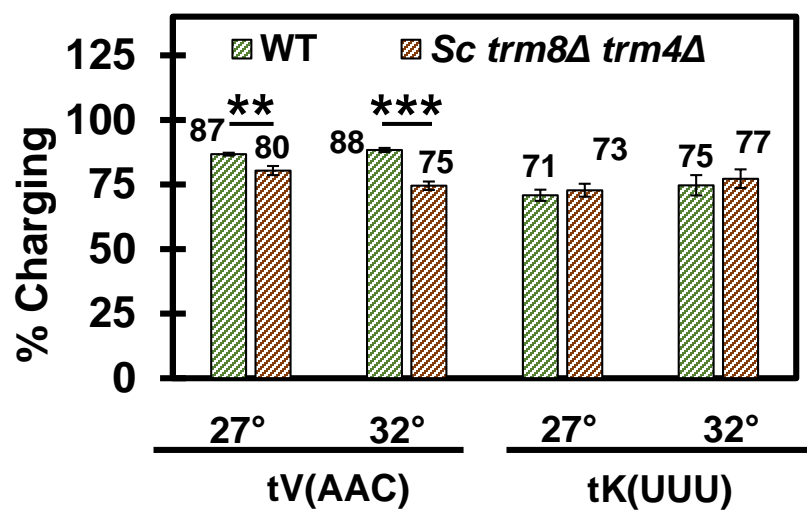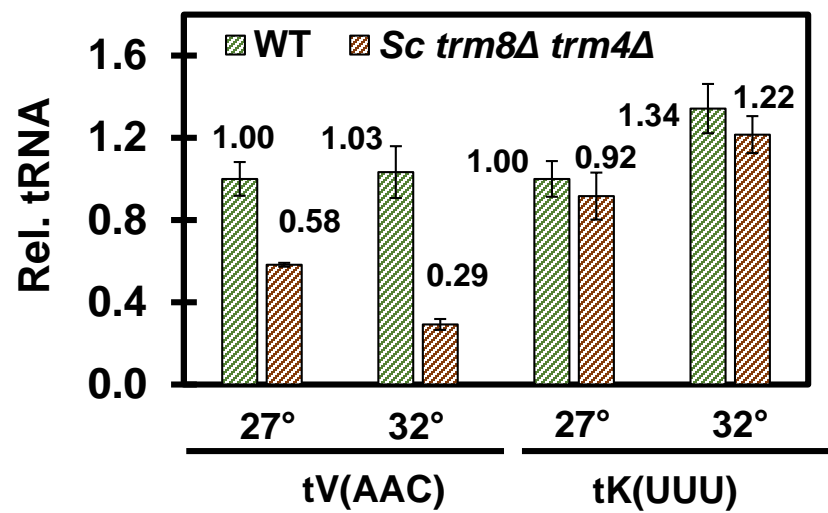

Supplement: S23 Fig — A. Analysis of tRNA charging levels in S. cerevisiae trm8Δ trm4Δ mutants after shift to 32°C. Cell pellets from the growth for Fig 6B were used to isolate acidic RNA and analyzed by acidic northern as described in S18 Fig. B. Quantification of tRNA charging and tRNA levels. The percent aminoacylation of tV(AAC) and tK(UUU) was calculated as described in S18 Fig, and relative tRNA levels were quantified as in Fig 6C. (PDF) [file pgen.1008893.s023.pdf]
