# Supplementary material for: Hypomodified tRNA in evolutionarily distant yeasts can trigger rapid tRNA decay to activate the general amino acid control response, but with different consequences
Source: PLoS Genet. 2020 Aug 25;16(8):e1008893. doi: 10.1371/journal.pgen.1008893 (PMC7473580; doi:10.1371/journal.pgen.1008893)
Supplement: S1 Table — (PDF) [file pgen.1008893.s024.pdf]

S1 Table. Variable loop sequences and the folding free energies of the acceptor stem/Tstem loop of predicted Trm8 substrate tRNAs of *S. pombe trm8Δ* mutants

tRNA	Variable loop sequence	ΔG^{o}_{37} (Kcal/mole) Acceptor/T stem loop
tC(GCA-1)	AGGUC	-16.0
tF(GAA-1)	UGGUC	-16.8
tT(CGU)	AGGUC	-16.8
tT(AGU)	AGGCC	-16.9
tK(UUU)	AGGUU	-17.0
tP (AGG)	UGGUC	-17.7
tA(AGC)	AGGUC	-17.7
tR(CCU)	AGGUU	-18.2
tC(GCA-2)	AGGUC	-18.3
tF(GAA-2)	UGGUC	-18.5
tV(AAC)	CGGUC	-19.9
tMe (CAU)	AGGUU	-20.2
tY(GUA)	UGGUC	-20.5
tP(UGG-1)	UGGUC	-20.6
tW(CCA)	AAGUC	-20.7
tV(UAC)	CGGUC	-20.9
tA(UGC)	UCGUC	-22.0
tK(CUU)	AGGUU	-22.2
tP(UGG-1)	UGGUC	-23.7
tI(AAU)	AUGUC	-24.0
tA(CGC)	AGGUC	-24.2
tN(GUU)	AGGUC	-25.2
tMi(CAU)	AGGUU	-25.5
tV(CAC)	AGGUC	-25.5
