# Supplementary material for: Hypomodified tRNA in evolutionarily distant yeasts can trigger rapid tRNA decay to activate the general amino acid control response, but with different consequences
Source: PLoS Genet. 2020 Aug 25;16(8):e1008893. doi: 10.1371/journal.pgen.1008893 (PMC7473580; doi:10.1371/journal.pgen.1008893)
Supplement: S2 Table — (PDF) [file pgen.1008893.s025.pdf]

S2 Table. GAAC mutations identified in *S. pombe trm8Δ* suppressors

Strain	Mutation
Sp trm8 gcn1-1	Inframe deletion in V2193-A2195
Sp trm8 gcn2-1	Start lost, G3A
Sp trm8 gcn2-2	Frameshift at R71
Sp trm8 gcn2-3	Stop at W982
Sp trm8 tif221-1	Stop at Q257
Sp trm8 tif221-2	Frameshift at L43
