# Supplementary material for: Hypomodified tRNA in evolutionarily distant yeasts can trigger rapid tRNA decay to activate the general amino acid control response, but with different consequences
Source: PLoS Genet. 2020 Aug 25;16(8):e1008893. doi: 10.1371/journal.pgen.1008893 (PMC7473580; doi:10.1371/journal.pgen.1008893)
Supplement: S3 Table — (PDF) [file pgen.1008893.s026.pdf]

S3 Table. ***S. cerevisiae*** **strains used in this study**

Strain name	Parent strain	Genotype	Source
YTD 386	AA 527	*trm8Δ::natMX trm4Δ::kanMX*	[1]
YTD 558	YTD 386	*trm8Δ::natMX trm4Δ::BleMX*	This study
YTD 559	YTD 558	*trm8Δ::natMX trm4Δ::BleMX gcn2Δ::KanMX*	This study
YTD 561	YTD 566	*gcn2Δ::KanMX*	This study
YTD 562	AA 413	*trm8Δ::NatMX*	This study
YTD 563	YLN 5	*trm1Δ::BleMX*	This study
YTD 564	ISC 188	*tan1Δ::KanMX*	This study
YTD 565	ISC 834	*tan1Δ::KanMX trm44Δ::natMX*	[2]
YTD 566	ISC 534	WT MATa	This study
YTD 567	YTD 558	*trm8Δ::natMX trm4Δ::BleMX gcn1Δ::KanMX*	This study
YTD 568	YTD 558	*trm8Δ::natMX trm4Δ::BleMX gcn4Δ::KanMX*	This study
YTD 569	YTD 562	*trm8Δ::NatMX gcn2Δ::KanMX*	This study
YTD 570	YTD 563	*trm1Δ::NatMX gcn2Δ::KanMX*	This study
YTD 571	YTD 564	*tan1Δ::BleMX*	This study
YTD 572	YTD 565	*tan1Δ::BleMX trm44Δ::natMX*	This study
YTD 573	YTD 566	*gcn1Δ::KanMX*	This study
YTD 574	YTD 566	*gcn4Δ::HygMX*	This study
YTD 575	YTD 559	*trm8Δ trm4Δ gcn2Δ met22Δ::HygMX*	This study
YTD 576	YTD 571	*tan1Δ::BleMX gcn2Δ::KanMX*	This study
YTD 577	YTD 572	*tan1Δ::BleMX trm44Δ::natMX gcn2Δ::KanMX*	This study

References

1. Alexandrov A, Chernyakov I, Gu W, Hiley SL, Hughes TR, Grayhack EJ, et al. Rapid tRNA decay can result from lack of nonessential modifications. Mol Cell. 2006;21(1):87-96.

2. Kotelawala L, Grayhack EJ, Phizicky EM. Identification of yeast tRNA Um(44) 2'-O-methyltransferase (Trm44) and demonstration of a Trm44 role in sustaining levels of specific tRNA(Ser) species. RNA. 2008;14(1):158-69.
