# Supplementary material for: Hypomodified tRNA in evolutionarily distant yeasts can trigger rapid tRNA decay to activate the general amino acid control response, but with different consequences
Source: PLoS Genet. 2020 Aug 25;16(8):e1008893. doi: 10.1371/journal.pgen.1008893 (PMC7473580; doi:10.1371/journal.pgen.1008893)
Supplement: S4 Table — (PDF) [file pgen.1008893.s027.pdf]

S4 Table. Plasmids used in this study

Plasmid	Parent	Description	Source
pREP3X		*LEU2* P_{nmt1}	[1]
pREP41X		*LEU2* P_{nmt1*}	[1]
pREP81X		*LEU2* P_{nmt1**}	[1]
AB 553-1	pREP3X	*LEU2* with a NotI between PstI and P_{nmt1}	This study
ETD 67-1	AB 553-1	*LEU2* P_{trm8} *trm8*$^{+}$	This study
ETD 70-1	AB 553-1	*LEU2 SPATRNAPRO.05 SPBTRNAPRO.05*	This study
ETD 113-1	AB 553-1	*LEU2 SPATRNATYR.01*	This study
ETD 115-1	AB 553-1	*LEU2 SPATRNATYR.01 SPBTRNAPRO.05*	This study
ETD 104-1	AB 553-1	*LEU2* P_{dhp1} *dhp1*$^{+}$	This study
ETD 109-1	pREP81X	*LEU2* $P_{nmt1***}$ *gcn2+*	This study
ETD 119-1	pREP41X	*LEU2* P_{nmt1**} *tif221+*	This study
ETD 126	EIC-17	tV(AAC)E2 URA3 2u	[2]

References

1. Forsburg SL. Comparison of *Schizosaccharomyces pombe* expression systems. Nucleic Acids Res. 1993;21(12):2955-6.

2. Chernyakov I, Whipple JM, Kotelawala L, Grayhack EJ, Phizicky EM. Degradation of several hypomodified mature tRNA species in *Saccharomyces cerevisiae* is mediated by Met22 and the 5'-3' exonucleases Rat1 and Xrn1. Genes Dev. 2008;22(10):1369-80.
