# Supplementary material for: Hypomodified tRNA in evolutionarily distant yeasts can trigger rapid tRNA decay to activate the general amino acid control response, but with different consequences
Source: PLoS Genet. 2020 Aug 25;16(8):e1008893. doi: 10.1371/journal.pgen.1008893 (PMC7473580; doi:10.1371/journal.pgen.1008893)
Supplement: S5 Table — (PDF) [file pgen.1008893.s028.pdf]

S5 Table. Oligomers used for northern analysis

Name	Target RNA[a]	Probe (5'-3')	Sequence
TDZ 114	tV(AAC)	73-54	TGATCGTGCCAAGACTCGAA
TDZ 115	tMi(CAU)	73-55	TTGCGCGGCCAGGTTTCGA
TDZ 123	tMe(CAU)	20-1	ACCGACTGAGCTACAGAAGC
TDZ 124	tK(CUU)	73-55	CTCCCAAGGCGAGACTCGA
TDZ 125	tK(UUU)	73-54	CTCCCACTGCGAGATTCGAA
TDZ 126	tP(AGG)	72-53	GGGCTGTTGTGGGAATCGAA
TDZ 128	tY(GUA)	73-52	TCTCCTGAGCCAGAATCGAACT
TDZ 129	tV(CAC)	23-1	TCATACCACTAGAACACGGGACT
TDZ 130	tV(UAC)	73-52	TGCATCTACCAGGGATCGAAC
TDZ 131	tI(AAU)	73-55	TGGTCACAGCCGGGTTCGA
TDZ 132	tN(GUU)	73-57	CGGTCAGGGAGGGACTC
TDZ 137	tA(AGC)	73-50	GGACAAGCCAGAACTCGAATCT
TDZ 138	tA(CGC)	73-54	TGGAGATGCCGGGAATCGAA
TDZ 140	tT(AGU)	72-54	GCTCCAGCAGTGACTCGAA
TDZ 141	tT(CGU)	54-34	ACACTGGACCTCCTGTTTACG
TDZ 143	tP(UGG)	27-4	AGTATGCTACCACTACACCACGAA
TDZ 155	5.8S rRNA	60-37	GCTGCGTTCTTCATCGATGCG
TDZ 177	tP(AGG)	27-5	GGTATCATACCACTAGACCAAAC
TDZ 182	tR(CCU)	39-17	CTCGTTAGGAATGAGATGCGCTA
TDZ 184	tC(GCA)	22-1	CTAACCACTGAGCTATGACCCC
TDZ 185	tW(CCA)	22-1	CTACCAACTGAGTTAAGGGGCC
TDZ 186	tA(UGC)	73-54	TGGACACGCTGGGGATTGAA
TDZ 364	5S rRNA	58-33	TAACTGCAGTGATCGGACGGG
TDZ 395	pre-tY(GUA):2	In10-26	ATCGTTTTAATTACAGCCGGATG
TDZ 396	pre-tY(GUA):1-1, 1-2	41-In-29	CGGTATCTTTATAATTACAGCCGG
TDZ 397	pre-tY(GUA):1-3	41-In-29	CGGTATCATCGAAATTACAGCCGG
LH 735	tL(UAA)	73-54	TGCGGCCAGAGAGGTTCGAA
LH 737	tG(GCC)	73-53	TGCTTTGGCCGGGAATCGAAC
Val(CCA)	*tV(AAC)*	*76-60*	*TGGTGATTTCGCCCAGGA*
tRNAPheloop	*tF(GAA)*	*64-45*	*TGTGGATCGAACACAGGACC*
TrpP1	*tW(CCA)*	*33-15*	*AGT CGA AAG CTC TAC CAT*
5S	*5S*	*20-1*	*TGGTAGATATGGCCGCAACC*
5'-tRNALys(UUU)	*tK(UUU)*	*18-1*	*CAACTGAGCTAACAAGGA*
LeuCAAP1	*tL(CAA)*	*62-e3*	*AGATTCGAACTCTTGCATCTT*
5'-tRNAArg(UCU)	*tR(UCU)*	*24-5*	*CGTTGCCATTACGCCACGC*
TDZ 415	*pre-tV(AAC)*	*+9-60*	*AAAAAATGATTTCGCCCAGG*

[a]Note that *S. cerevisiae* oligomers are indicated in italics
