# Supplementary material for: Hypomodified tRNA in evolutionarily distant yeasts can trigger rapid tRNA decay to activate the general amino acid control response, but with different consequences
Source: PLoS Genet. 2020 Aug 25;16(8):e1008893. doi: 10.1371/journal.pgen.1008893 (PMC7473580; doi:10.1371/journal.pgen.1008893)
Supplement: S6 Table — (PDF) [file pgen.1008893.s029.pdf]

S6 Table. Oligomers used for tRNA purifications

Name	Target RNA	Probe (5'-3')	Sequence
TDZ 342	tF(GAA)	76-53	biotin-TGGTGTCACAAACCGGGATCGAAC
TDZ 358	tY(GUA)	76-64	biotin-TGGTCTCCTGAGCCAGAATCGAACTA
TDZ 359	tP(AGG)	76-54	biotin-TGGGGGCTGTTGTGGGAATCGAA
